# Direct intramolecular double cross-dehydrogentive-coupling (CDC) cyclization of *N*-(2-pyridyl)amidines under metal-free conditions†

**DOI:** 10.1039/c9ra09265j

**Published:** 2019-12-19

**Authors:** Fengping Yi, Chao Fu, Qihui Sun, Huazhen Wei, Genfa Yu, Weiyin Yi

**Affiliations:** School of Perfume and Aroma Technology, Shanghai Institute of Technology Shanghai 201418 P. R. China yiwy@sit.edu.cn shyugenfa@163.com

## Abstract

A facile transition-metal-free protocol to form 2-iminoimidazo[1, 2-*a*]-pyridines bearing a –CHBr_2_ group and an aza-quaternary carbon center at the 3 position from *N*-(2-pyridyl)amidines substrates, in which the new heterocyclic skeletons constructed from amidines *via* radical reactions or nucleophilic substitution reactions are promoted only by CBr_4_ under mild conditions, is demonstrated. The reactions were realized by intramolecular CDC reaction involving C–N and C–C bond formation *via* the sequential C(sp^3^)–H bifunctionalization mode on the same carbon atom under mild conditions. Moreover, this work also provides an excellent and representative example for CBr_4_ as an efficient reagent to initiate radical reactions under initiator-free conditions or to give rise to nucleophilic substitution reactions only by base.

## Abstract 2

A metal-free protocol to form 2-iminoimidazo[1,2-*a*]-pyridines from *N*-(2-pyridyl)amidines, in which the constructed heterocyclic skeletons are promoted by CBr_4_*via* radical or nucleophilic substitution reactions under mild conditions, is demonstrated.

## Graphical abstract

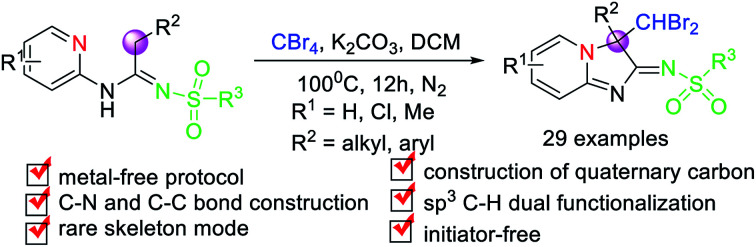

## Introduction

C–H functionalization, especially sp^3^ C–H bond functionalization, to efficiently construct C–X (X = C, N, O, S, *etc.*) bonds has been one of the most researched topics in the field of organic chemistry because the formation of C–X (X = C, N, O, S, *etc*) bonds is a fundamental organic reaction.^1^ Previous reports on C–H functionalization to build new C–X (X = C, N, O, S, *etc*) bonds required the prefunctionalization of the substrates, which caused unnecessary waste, great costs, and laborious experimental handling. Moreover, some cross-coupling reactions generally needed transition-metal catalysts and special ligands,^2^ which would also cause heavy metal contamination for underground water and soil. Therefore, it is still highly desirable to further develop new atom- and step-economic, greener approaches to construct C–X (X = C, N, O, S, *etc*) bonds by direct C–H functionalization. More recently, cross-dehydrogenative-coupling (CDC) reactions,^3^ especially transition-metal-free CDC reaction, which can introduce a substituent through the direct cleavage of a C–H bond under redox conditions without the introduction of a leaving group,^4^ have emerged as a valuable tool for this transformation and have also gained significant attention because this strategy presented a non-metallic, environmentally friendly, and concise way compared to other previous available methods. For example, the formation of C–X (X = C, N, O, S, *etc*) bonds can be achieved by the metal-free CDC reaction under only oxidants such as peroxides,^5^ quinones^6^ and hypervalent iodine reagents,^7^ O_2_,^8^ or KO^*t*^Bu/DMF.^9^ Though every above-mentioned metal-free CDC protocol has its own advantages, further exploitation of more simple, efficient and metal-free CDC approaches to forge C–X (X = C, N, O, S, *etc*) bonds using various novel mediators under mild conditions is still the goal pursuit by many scientific workers.

Carbon tetrabromide (CBr_4_), as a commercially available and cheap reagent, which has been utilized as a organocatalyst or a stoichiometric reagent for a variety of organic transformations, has attracted considerable attention from chemists.^10^ Some literature surveys showed that CBr_4_ was used to catalyze the deprotection of trialkylsilyl esters and *b*-(trimethylsilyl)ethoxymethyl ethers,^10*b*,11^ esterifications,^10*h*^ expoxide ring opening,^12^ the acetalization of aldehydes,^13^ the Friedel–Crafts alkylation indoles with carbonyl compounds,^14^ the carboxylation of indoles with CBr_4_/MeOH,^15^ and esterification of methyl aromatic,^16^*etc*. Furthermore, most importantly, it has been found that CBr_4_ also played an extremely important role in the formation of C–X (X = C, N, O, S, *etc*) bonds in the field of cross-dehydrogenation coupling (CDC) reactions to construct the physiological and biological active compounds *via* C–H functionalization under metal-free conditions.^17^ For example, Huang's group developed an efficient and facile CBr_4_-mediated CDC reaction to form the C–O bond and C–S bond under metal-free conditions.^17*a*,*b*^ Equally, the Huo and other groups have also demonstrated a series of the CBr_4_-promoted CDC and DOD reactions *via* C–C bond and C–N bond formation to construct successfully complex heterocyclic compounds such as imidazo[1, 2-*a*]pyridines, imidazo[1, 2-*a*]pyrimidines and imidazoles.^17*c*–*k*^ The advancements of these reactions clearly showed that CBr_4_ had great potential in organic synthesis. Therefore, it is an urgent mission for organic chemists to further develop its greater potential in organic chemistry at present.

**Scheme 1:**
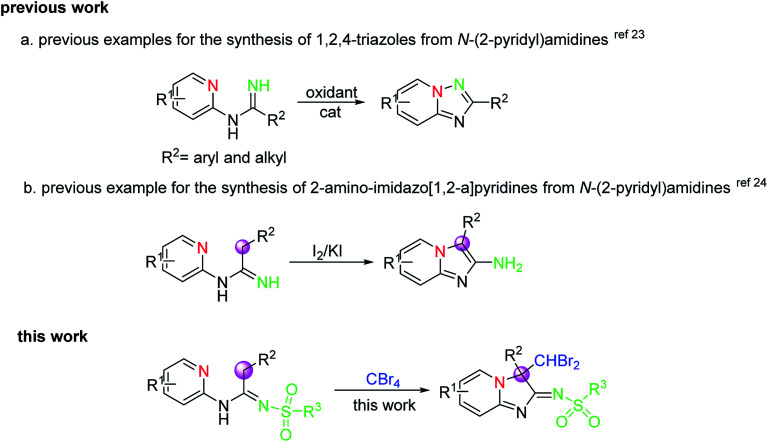
Previous works on the oxidative cyclization of *N*-(2-pyridyl)amidines and this work.

As an important class of organic synthons, amidines have been frequently applied in the synthesis of various heterocyclic ring systems such as quinazolines,^18^ quinazolinones,^19^ pyrimidines,^20^ triazoles,^21^ and benzimidazoles.^22^ Especially importantly, *N*-(2-pyridyl) amidines, as one of the most important *N*-aryl amidines, have also been employed for the formation of different biological active compounds bearing nitrogen-containing heterocyclic skeletons, including 1, 2, 4-triazoles and imidazo[1, 2-*a*]-pyridines, by the direct C–H functionalization for N–N and C–N bond formation in the presence of a catalyst and oxidant. For example, when a large variety of oxidants were used, including air,^23*a*,*g*^ PIFA (phenyliodinebis(trifluoroacetate)),^23*b*^ NaClO,^23*c*^ Pb(OAc)_4_,^23*e*,*g*^ and I_2_,^23*f*^ 1,2,4-triazoles with various substituents can be afforded by intramolecular oxidative N–N bond formation from *N*-(2-pyridyl)amidines substrates (Scheme 1a). Significantly, in 2016, Chang's group^24^ reported one example for the synthesis of 2-aminoimidazo[1, 2-*a*]-pyridines from *N*-(2-pyridyl)amidines *via* intramolecular oxidative C–N bond formation using I_2_/KI as reagent (Scheme 1b), in which the bonding mode of reaction was completely different from a previously reported one by Chang and co-workers^23*g*^ even under the same reaction conditions. These reports, as a consequence, indicated clearly that the results of the reaction could be greatly affected by the structure of the substrates and the reaction conditions. Although great progress has already been made in this area, there is still an urgent requirement to develop highly efficient and environmentally benign synthetic methods to construct the diverse core framework in structure *via* oxidative CDC strategies on account of the increasing demands of structural novelty and diversity in both biomedical research and drug discovery. Accordingly, in view of our continuous interest in amidines and ketenimines,^25^ we herein report a transition-metal-free oxidative CDC cyclization reaction of *N*-(2-pyridyl)amidines *via* the sequential dual C–H functionalization of the C(sp^3^)–H bond on the same carbon atom involving C–N and C–C bond formation, in which the new heterocyclic skeletons constructed from amidines *via* radical reactions or nucleophilic substitution reactions are promoted only by CBr_4_ under mild conditions.

## Results and discussion

The desire to create novel and diverse compounds in structure continues to activate us to investigate the reaction process of amidines by using CBr_4_. Firstly, the oxidative cyclic conditions for the CDC strategy were optimized. To our delight, the reaction of *N*-(2-pyridyl)amidine (1a) and CBr_4_ (2) was carried out at room temperature for 10 h in the presence of K_2_CO_3_ under N_2_, giving the corresponding imidazo[1, 2-*a*]-pyridine core-like product in 25% yield (Table 1, entry 1). 3a had been confirmed by ^1^H NMR, ^13^C NMR, and HRMS. Morever, we also observed that when the reaction temperature was elevated to 60 °C, the yield of 3a was also increased to 37% accordingly (Table 1, entry 2). To further increase the yield of 3a, the reactions were performed under different bases. As shown in Table 1 (Table 1, entries 2–7), it was found that K_2_CO_3_ turned out to be the best base in improving the yield of CDC reaction (entry 2, 37%). However, when TEA and DBU as bases were employed, two reactions furnished 3a only in trace amounts (entries 4 and 7). We, subsequently, attempted to perform the reaction in various commonly used solvents. It was shown that, in contrast to DCM, the use of other solvents was found to be less effective (Table 1, entry 2 and entries 8–12). To render further improve the yield of the transformation, a change in the amount of reaction substrates was also investigated. The results indicated that the molar ratio of 1a : 2 was enhanced to 1 : 1.2, the yield of reaction products was the best (Table 1, entries 13–19). However, it could be found that if the amount of CBr_4_ was further increased, the yield of the product 3a would decrease (Table 1, entry 13, 31% yield). In addition, it was noteworthy that the longer reaction time and the more suitable higher temperature were required for the formation of 3a (Table 1, entries 16–19). Finally, the optimized reaction conditions were obtained as follows: the CDC reaction system with the ratio of 1 : 1.2 (1a : 2) was carried out at 100 °C in DCM for 12 h in the presence of K_2_CO_3_ under N_2_.

**Table 1:** Optimization of the reaction conditions a

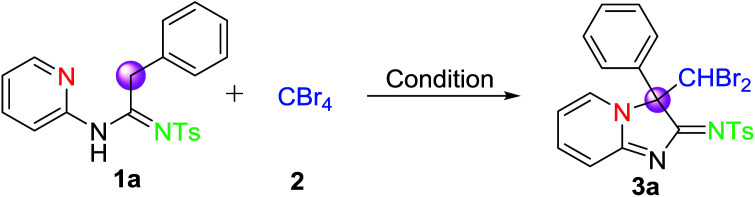				
Entry	Catalyst	Solvent	Temp/°C	Yield^b^ [%]
1	K_2_CO_3_	DCM	rt	25
2	K_2_CO_3_	DCM	60	37
3	Cs_2_CO_3_	DCM	60	30
4	TEA	DCM	60	Trace
5	LiO^*t*^Bu	DCM	60	22
6	KO^*t*^Bu	DCM	60	24
7	DBU	DCM	60	Trace
8	K_2_CO_3_	THF	60	Trace
9	K_2_CO_3_	DMF	60	Trace
10	K_2_CO_3_	MeCN	60	34
11	K_2_CO_3_	DMSO	60	Trace
12	K_2_CO_3_	Dioxane	60	28
13	K_2_CO_3_	DCM	60	41
14	K_2_CO_3_	DCM	60	45
15	K_2_CO_3_	DCM	80	57
16	K_2_CO_3_	DCM	100	68
17	K_2_CO_3_	DCM	120	66
18^c^	K_2_CO_3_	DCM	100	78
19^d^	K_2_CO_3_	DCM	100	74

a Reaction conditions: entries 1–12, 1a (0.2 mmol), 2 (0.2 mmol), base (0.4 mmol) in solvent (2 mL), stirred at room temperature for 10 hours under N_2_; entry 13, the molar ratio of 1a : 2 = 1 : 1.5, the amount of other conditions is unchanged. Entries 14–20, the molar ratio of 1a : 2 = 1 : 1.2, the amount of other conditions is unchanged.

b An isolated yield of 3a is given, 1a is used as a reference.

c The reaction was carried out for 12 hours.

d The reaction was carried out for 14 hours.

Under the optimized conditions given above, the scope and generality of the reaction in regard to different *N*-(2-pyridyl)amidines (1), which were furnished from the reaction of 2-amino pyridines, terminal alkynes and sulfonyl azides under Cu(i) and base,^25,26^ were investigated, and the results are summarized in Table 2. All tested amidines reacted smoothly with CBr_4_ were efficiently transformed into their corresponding products (Table 2, 3a–3ad) with moderate to good yields, reflecting wide scope of this CDC reaction system. The structure of product 3a was also further confirmed by single-crystal X-ray diffraction, as also shown in Table 2. Moreover, it was found that the yields remained relatively stable and only *N*-(2-pyridyl)amidines derived from terminal alkynes with electron-withdrawing group on phenyl rings afforded slightly lower yields of products than those from other terminal alkynes (Table 2, 3a–3i). Meanwhile, the effect of different *N*-(2-pyridyl)amidines from sulfonyl azides was surveyed too. The results indicated that the yields of products derived from sulfonyl azides bearing electron-donating group on phenyl rings were obviously higher than those of products without substituents on phenyl rings of sulfonyl azides, for example, 3a and 3x or 3b and 3y.

**Table 2:** Substrate scopes of CDC reaction system for the formation of 3^a^

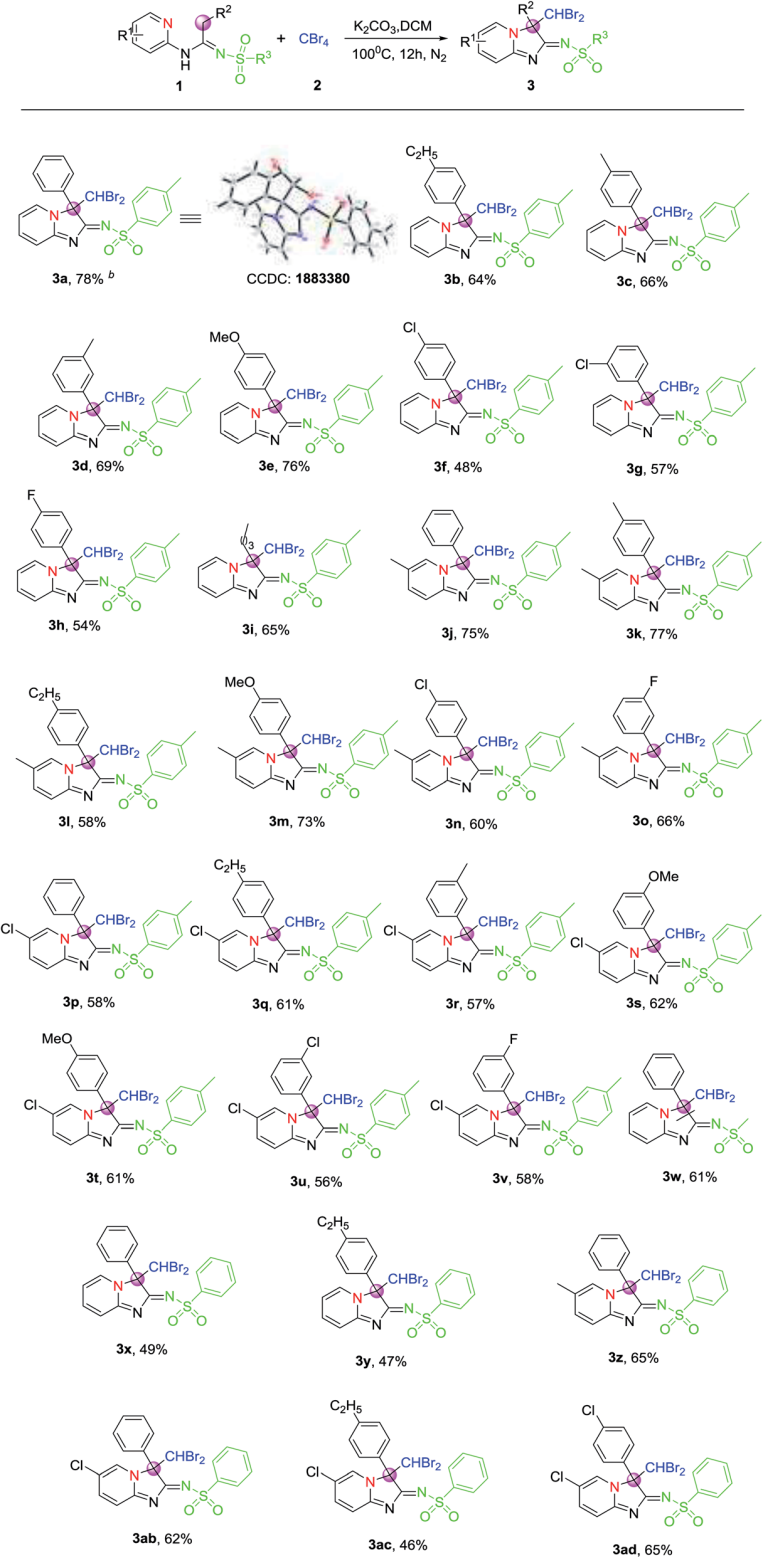

a Optimal reaction conditions: 1 (0.2 mmol), 2 (0.24 mmol), K_2_CO_3_ (0.4 mmol) in dry DCM (2 mL), sealed and then stirred at 100 °C for 12 h under N_2_.

b Isolated yield of 3 are given, 1 as reference.

**Scheme 2:**
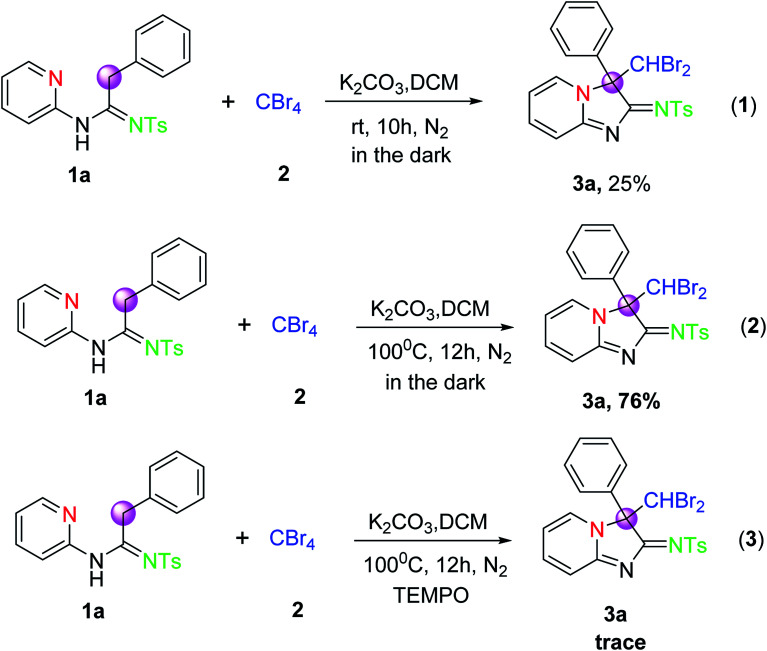
Control experiments.

From the results of the investigation in Table 2, we found that an appropriate temperature rise for the reaction would contribute to the formation of the products 3. To further gain mechanistic insights into this transformation, a series of control experiments were performed under the envisaged conditions. However, when the reaction was carried out in the dark according to eqn (1) in Scheme 2 under the reaction conditions of entry 1 (Table 1), the yield is equal to that of entry 1. Similarly, the reaction was performed in the dark according to eqn (2) in Scheme 2 under standard reaction conditions, the desired product 3a was also isolated in 76% yield, which was also close to that of the reaction in Table 1 (entry 18, 78%). These results indicated that the visible light was not essential for the successful completion of the reaction. In addition, the reaction of 1a and 2 was conducted in the presence of 1 equiv. of TEMPO as a radical scavenger under the optimized conditions, only the trace amount of 3a was observed. This result suggested that radical processes might be involved in the CDC reaction system.

**Scheme 3:**
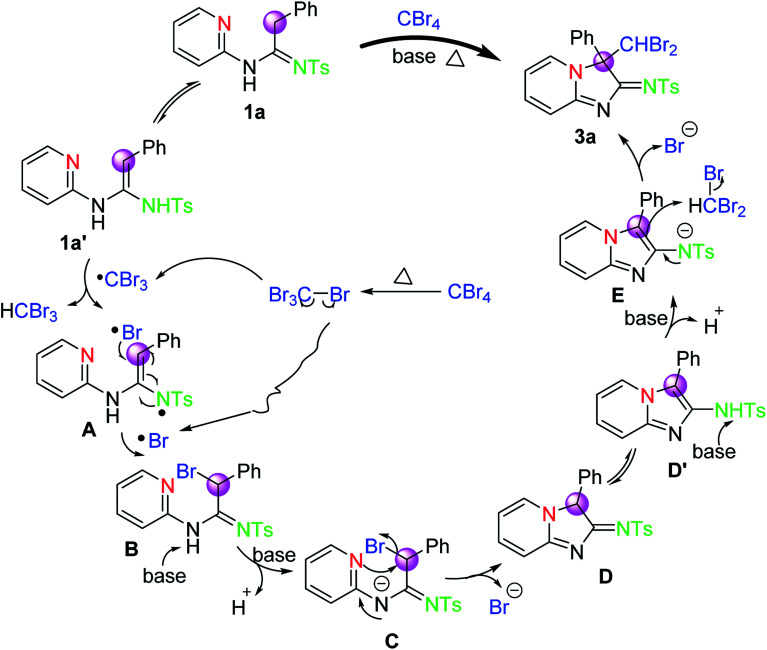
The tentative mechanistic pathway for the formation of 3a.

Based on these facts mentioned above and previous literatures,^17^ a tentative mechanism for the transformation of 1a and CBr_4_ into 3a is proposed, as depicted in Scheme 3. Initially, substrate 1a can easily tautomerize into intermediate 1a′ in the presence of the α-hydrogen of the amidine group under such reaction conditions. And then, a hydrogen atom of intermediate 1a′ is abstracted by CBr_3_ radical, which is derived from the homolytic cleavage of the C–Br bond of CBr_4_ upon heating accompanied by the formation of Br radical, to deliver the radical intermediate A. Intermediate A reacts rapidly with Br radical to afford intermediate B. Subsequently, a base-promoted intramolecular nucleophilic substitution of intermediate C, which is achieved from intermediate B in the presence of base, will occur to provide intermediate D. Intermediate D will tautomerize into intermediate D′ again. Finally, the desired product 3a*via* intermediate E will be provided by the nucleophilic substitution of intermediate D′ with CHBr_3_ under base. Meanwhile, we can also not rule out another possible mechanism route that experienced a base promoted nucleophilic substitution. Thus, another possible mechanism process is also described by us (see ESI†).

In conclusion, we have demonstrated a facile metal-free strategy to form 2-iminoimidazo[1, 2-*a*]-pyridines by CBr_4_-mediated intramolecular CDC reaction under mild conditions, in which the new heterocyclic skeletons were constructed from amidines by the sequential C(sp^3^)–H bifunctionalization mode on the same carbon atom involving C–N and C–C bond formation. The surveys could better reveal that the results of the reaction depended greatly on the structure of the substrates and the reaction conditions. Moreover, this approach further indicates that amidines have abundant reactivity under various reaction conditions again. This work also provides an excellent example for CBr_4_ as an efficient reagent to initiate radical reactions under initiator-free conditions or to give rise to nucleophilic substitution reactions only by base.

## Experimental section

### General remarks

All reagents were purchased from commercial suppliers, and were used without further purification. All solvents were treated according to standard procedures. The progress of reactions was monitored by TLC. For chromatographic purifications, 200–300 mesh silica gel was used. ^1^H (500 MHz) and ^13^C (126 MHz) NMR spectra were recorded with tetramethylsilane as an internal standard. HRMS measurements were carried out using the ESI ionization technique with an FT-ICR analyzer. Data are reported as follows: chemical shift, multiplicity (s = singlet, d = doublet, t = triplet, q = quartet, m = multiplet), coupling constants (Hz) and integration. CCDC (for 3a) contains the supplementary crystallographic data for this paper.

### Procedure for generation of *N*-(2-pyridyl)amidines 1

*N*-(2-Pyridyl)amidines (1) were prepared as reported in the literature^1*f*,26*a*^ according to the reaction equation (see ESI†) or are commercially available.

### General procedure for the synthesis of 3

Under N_2_, a mixture of *N*-(2-pyridyl)amidines (1) (0.2 mmol), CBr_4_ (2) (0.24 mmol), K_2_CO_3_ (0.3 mmol), in dry DCM (2 mL) was added to a sealed tube. And then, the mixture was stirred at 100 °C for 12 h. The reaction mixture was then cooled to room temperature. The solvent was removed under reduced pressure to give a residue. The crude product was purified on silica gel column chromatography using ethyl acetate and petroleum ether as the eluent to afford the desired products 3.

### Characterization data for 3

#### *N*-(3-(Dibromomethyl)-3-phenylimidazo[1,2-*a*]pyridin-2(3*H*)-ylidene)-4-methylbenzenesulfonamide (3a)

A yellow solid (78%). Mp: 216–218 °C. ^1^H NMR (500 MHz, chloroform-*d*) *δ* 8.42 (d, *J* = 6.4 Hz, 1H), 8.02 (t, *J* = 7.6 Hz, 1H), 7.85 (d, *J* = 8.1 Hz, 2H), 7.49 (d, *J* = 8.8 Hz, 1H), 7.37 (d, *J* = 7.7 Hz, 3H), 7.17 (d, *J* = 7.9 Hz, 2H), 7.09 (d, *J* = 7.7 Hz, 3H), 6.59 (s, 1H), 2.35 (s, 3H). ^13^C NMR (126 MHz, chloroform-*d*) *δ* 176.06, 165.89, 145.29, 142.25, 138.75, 135.34, 133.92, 129.73, 129.33, 128.62, 127.61, 125.53, 117.68, 114.59, 81.42, 45.46, 21.53. HRMS: calcd for C_21_H_17_Br_2_N_3_O_2_S [M + H]^+^ 533.9481; found 533.9486.

#### *N*-(3-(Dibromomethyl)-3-(4-ethylphenyl)imidazo[1,2-*a*]pyridin-2(3*H*)-ylidene)-4-methylbenzenesulfonamide (3b)

A light yellow solid (64%). Mp: 227–229 °C ^1^H NMR (500 MHz, chloroform-*d*) *δ* 8.42 (d, *J* = 6.5 Hz, 1H), 8.00 (t, *J* = 7.7 Hz, 1H), 7.87 (d, *J* = 8.2 Hz, 2H), 7.50 (d, *J* = 8.9 Hz, 1H), 7.18 (t, *J* = 9.4 Hz, 4H), 7.06 (t, *J* = 6.8 Hz, 1H), 7.00 (d, *J* = 8.2 Hz, 2H), 6.58 (s, 1H), 2.65 (q, *J* = 7.5 Hz, 2H), 2.36 (s, 3H), 1.23 (t, *J* = 7.6 Hz, 3H). ^13^C NMR (126 MHz, chloroform-*d*) *δ* 176.28, 166.01, 146.12, 144.77, 142.12, 138.89, 135.34, 131.23, 128.78, 128.56, 127.68, 127.54, 125.47, 117.70, 114.00, 81.36, 45.66, 28.40, 21.52, 15.15. HRMS: calcd for C_23_H_21_Br_2_N_3_O_2_S [M + H]^+^ 563.9774; found 563.9783.

#### N-(3-(Dibromomethyl)-3-(*p*-tolyl)imidazo[1,2-*a*]pyridin-2(3*H*)-ylidene)-4-methylbenzenesulfonamide (3c)

A light yellow solid (66%). Mp: 182–184 °C ^1^H NMR (500 MHz, chloroform-*d*) *δ* 8.40 (d, *J* = 6.5 Hz, 1H), 8.01 (t, *J* = 7.6 Hz, 1H), 7.86 (d, *J* = 8.2 Hz, 2H), 7.48 (d, *J* = 8.9 Hz, 1H), 7.16 (d, *J* = 7.9 Hz, 4H), 7.07 (t, *J* = 6.8 Hz, 1H), 6.96 (d, *J* = 8.2 Hz, 2H), 6.56 (s, 1H), 2.34 (d, *J* = 8.2 Hz, 6H). ^13^C NMR (126 MHz, chloroform-*d*) *δ* 176.27, 165.95, 144.96, 142.15, 139.95, 138.89, 135.35, 131.07, 129.99, 128.58, 127.67, 125.39, 117.68, 114.19, 81.33, 45.70, 21.54, 21.10. HRMS: calcd for C_22_H_19_Br_2_N_3_O_2_S [M + H]^+^ 549.9617; found 549.9625.

#### *N*-(3-(Dibromomethyl)-3-(*m*-tolyl)imidazo[1,2-*a*]pyridin-2(3*H*)-ylidene)-4-methylbenzenesulfonamide (3d)

A yellow solid (69%). Mp: 178–180 °C ^1^H NMR (500 MHz, chloroform-*d*) *δ* 8.40 (d, *J* = 6.5 Hz, 1H), 8.01 (t, *J* = 7.7 Hz, 1H), 7.84 (d, *J* = 8.2 Hz, 2H), 7.49 (d, *J* = 8.8 Hz, 1H), 7.24 (t, *J* = 7.7 Hz, 1H), 7.19–7.14 (m, 3H), 7.08 (t, *J* = 6.8 Hz, 1H), 6.85 (d, *J* = 6.8 Hz, 2H), 6.57 (s, 1H), 2.32 (d, *J* = 19.1 Hz, 6H). ^13^C NMR (126 MHz, chloroform-*d*) *δ* 176.09, 166.01, 144.82, 142.15, 139.24, 135.33, 133.91, 130.53, 129.16, 128.57, 127.64, 127.51, 126.00, 122.65, 117.68, 114.02, 81.37, 45.55, 21.61, 21.52. HRMS: calcd for C_22_H_19_Br_2_N_3_O_2_S [M + H]^+^ 549.9617; found 549.9627.

#### *N*-(3-(Dibromomethyl)-3-(4-methoxyphenyl)imidazo[1,2-*a*]pyridin-2(3*H*)-ylidene)-4-methylbenzenesulfonamide (3e)

A light yellow solid (76%). Mp: 155–157 °C ^1^H NMR (500 MHz, chloroform-*d*) *δ* 8.42 (d, *J* = 6.5 Hz, 1H), 8.00 (t, *J* = 7.7 Hz, 1H), 7.88 (d, *J* = 8.1 Hz, 2H), 7.49 (d, *J* = 8.8 Hz, 1H), 7.18 (d, *J* = 7.9 Hz, 2H), 7.04 (dd, *J* = 16.4, 7.8 Hz, 3H), 6.88 (d, *J* = 8.8 Hz, 2H), 6.55 (s, 1H), 3.82 (s, 3H), 2.37 (s, 3H). ^13^C NMR (126 MHz, chloroform-*d*) *δ* 176.59, 166.11, 160.59, 144.79, 142.27, 139.03, 135.41, 128.70, 127.81, 127.05, 126.06, 117.86, 114.78, 114.04, 81.17, 55.54, 46.00, 21.64. HRMS: calcd for C_22_H_19_Br_2_N_3_O_3_S [M + H]^+^ 565.9567; found 565.9573.

#### *N*-(3-(4-Chlorophenyl)-3-(dibromomethyl)imidazo[1,2-*a*]pyridin-2(3*H*)-ylidene)-4-methylbenzenesulfonamide (3f)

A light yellow solid (48%). Mp: 172–174 °C ^1^H NMR (500 MHz, chloroform-*d*) *δ* 8.41 (s, 1H), 7.84 (d, *J* = 7.4 Hz, 2H), 7.58 (s, 1H), 7.42–7.37 (m, 2H), 7.33–7.30 (m, 2H), 7.18 (d, *J* = 7.4 Hz, 2H), 7.05–7.02 (m, 2H), 6.57 (s, 1H), 2.35 (s, 3H). ^13^C NMR (126 MHz, chloroform-*d*) *δ* 175.69, 165.56, 145.58, 142.56, 138.54, 136.09, 135.24, 132.50, 129.61, 129.10, 128.80, 127.60, 127.31, 127.07, 118.01, 114.93, 81.07, 45.19, 21.59. HRMS: calcd for C_21_H_16_Br_2_ClN_3_O_2_S [M + H]^+^ 569.9070; found 569.9074.

#### *N*-(3-(3-Chlorophenyl)-3-(dibromomethyl)imidazo[1,2-*a*]pyridin-2(3*H*)-ylidene)-4-methylbenzenesulfonamide (3g)

A yellow solid (57%). Mp: 161–163 °C ^1^H NMR (500 MHz, chloroform-*d*) *δ* 8.42 (d, *J* = 6.5 Hz, 1H), 8.04 (t, *J* = 7.7 Hz, 1H), 7.86 (d, *J* = 8.1 Hz, 2H), 7.53 (d, *J* = 8.8 Hz, 1H), 7.39 (d, *J* = 8.0 Hz, 1H), 7.34 (t, *J* = 7.9 Hz, 1H), 7.19 (d, *J* = 8.0 Hz, 2H), 7.10 (t, *J* = 7.6 Hz, 2H), 7.02 (s, 1H), 6.53 (s, 1H), 2.37 (s, 3H). ^13^C NMR (101 MHz, chloroform-*d*) *δ* 175.21, 165.38, 145.62, 142.12, 138.24, 135.63, 135.05, 130.42, 129.64, 129.07, 128.39, 127.15, 125.77, 125.51, 123.82, 117.32, 80.73, 44.85, 21.14. HRMS: calcd for C_21_H_16_Br_2_ClN_3_O_2_S [M + H]^+^ 569.9070; found 569.9070.

#### *N*-(3-(Dibromomethyl)-3-(4-fluorophenyl)imidazo[1,2-*a*]pyridin-2(3*H*)-ylidene)-4-methylbenzenesulfonamide (3h)

A yellow solid (54%). Mp: 203–205 °C ^1^H NMR (500 MHz, chloroform-*d*) *δ* 8.42 (d, *J* = 6.5 Hz, 1H), 8.02 (t, *J* = 8.1 Hz, 1H), 7.87 (d, *J* = 8.1 Hz, 2H), 7.51 (d, *J* = 8.9 Hz, 1H), 7.19 (d, *J* = 7.9 Hz, 2H), 7.14–7.10 (m, 2H), 7.08 (d, *J* = 7.7 Hz, 3H), 6.55 (s, 1H), 2.37 (s, 3H). ^13^C NMR (126 MHz, chloroform-*d*) *δ* 175.79, 165.67, 145.39, 142.17, 138.62, 135.19, 129.86, 128.47, 127.70, 127.64, 127.37, 117.42, 116.29, 116.11, 114.74, 80.83, 45.37, 21.33. HRMS: calcd for C_21_H_16_Br_2_FN_3_O_2_S [M + H]^+^ 553.9367; found 553.9376.

#### *N*-(3-Butyl-3-(dibromomethyl)imidazo[1,2-*a*]pyridin-2(3*H*)-ylidene)-4-methylbenzenesulfonamide (3i)

A light yellow solid (65%). Mp: 185–187 °C ^1^H NMR (500 MHz, chloroform-*d*) *δ* 8.27 (d, *J* = 6.4 Hz, 1H), 7.94 (dd, *J* = 21.5, 7.9 Hz, 3H), 7.41 (d, *J* = 8.8 Hz, 1H), 7.19 (d, *J* = 8.0 Hz, 2H), 7.04 (t, *J* = 6.8 Hz, 1H), 5.99 (s, 1H), 2.34 (s, 3H), 2.20 (s, 2H), 2.06 (s, 2H), 1.15–1.11 (m, 2H), 0.69 (t, *J* = 7.3 Hz, 3H). ^13^C NMR (126 MHz, chloroform-*d*) *δ* 176.65, 165.67, 144.34, 142.32, 138.73, 133.60, 128.59, 127.96, 117.47, 114.64, 79.27, 46.09, 38.38, 25.17, 22.15, 21.52, 13.52. HRMS: calcd for C_19_H_21_Br_2_N_3_O_2_S [M + H]^+^ 515.9774; found 515.9787.

#### *N*-(3-(Dibromomethyl)-6-methyl-3-phenylimidazo[1,2-*a*]pyridin-2(3*H*)-ylidene)-4-methylbenzenesulfonamide (3j)

A yellow solid (75%). Mp: 146–148 °C ^1^H NMR (500 MHz, chloroform-*d*) *δ* 8.21 (s, 1H), 7.86 (t, *J* = 8.4 Hz, 3H), 7.45 (d, *J* = 9.0 Hz, 1H), 7.39 (d, *J* = 7.6 Hz, 3H), 7.17 (d, *J* = 8.0 Hz, 2H), 7.09 (d, *J* = 6.9 Hz, 2H), 6.61 (s, 1H), 2.40 (s, 3H), 2.36 (s, 3H). ^13^C NMR (126 MHz, chloroform-*d*) *δ* 176.01, 164.56, 147.34, 142.03, 134.11, 133.07, 129.78, 129.64, 129.27, 128.86, 128.73, 128.54, 127.87, 127.63, 125.60, 124.73, 121.09, 117.20, 81.68, 45.60, 21.51, 18.04. HRMS: calcd for C_22_H_19_Br_2_N_3_O_2_S [M + Na]^+^ 571.9437; found 571.9442.

#### *N*-(3-(Dibromomethyl)-6-methyl-3-(*p*-tolyl)imidazo[1,2-*a*]pyridin-2(3*H*)-ylidene)-4-methylbenzenesulfonamide (3k)

A yellow solid (77%). Mp: 211–213 °C ^1^H NMR (500 MHz, chloroform-*d*) *δ* 8.18 (s, 1H), 7.86 (t, *J* = 8.3 Hz, 3H), 7.44 (d, *J* = 9.0 Hz, 1H), 7.16 (d, *J* = 5.2 Hz, 4H), 6.95 (d, *J* = 7.9 Hz, 2H), 6.57 (s, 1H), 2.38 (s, 3H), 2.34 (d, *J* = 4.2 Hz, 6H). ^13^C NMR (126 MHz, chloroform-*d*) *δ* 176.26, 164.44, 147.42, 141.98, 139.80, 133.09, 131.19, 129.92, 128.52, 127.62, 125.49, 124.80, 117.13, 81.62, 45.74, 21.51, 21.08, 18.03. HRMS: calcd for C_23_H_21_Br_2_N_3_O_2_S [M + H]^+^ 563.9774; found 563.9780.

#### *N*-(3-(Dibromomethyl)-3-(4-ethylphenyl)-6-methylimidazo[1,2-*a*]pyridin-2(3*H*)-ylidene)-4-methylbenzenesulfonamide (3l)

A light yellow solid (58%). Mp: 190–192 °C ^1^H NMR (500 MHz, chloroform-*d*) *δ* 8.20 (s, 1H), 7.86 (d, *J* = 8.2 Hz, 3H), 7.45 (d, *J* = 9.0 Hz, 1H), 7.18 (dd, *J* = 16.2, 8.1 Hz, 4H), 6.98 (d, *J* = 8.3 Hz, 2H), 6.60 (s, 1H), 2.65 (q, *J* = 7.5 Hz, 2H), 2.37 (d, *J* = 17.3 Hz, 6H), 1.24 (s, 3H). ^13^C NMR (126 MHz, chloroform-*d*) *δ* 176.26, 164.55, 147.14, 146.00, 141.95, 133.13, 128.73, 128.50, 127.65, 125.57, 124.50, 117.16, 81.66, 45.73, 28.39, 21.50, 18.02, 15.14. HRMS: calcd for C_24_H_23_Br_2_N_3_O_2_S [M + Na]^+^ 599.9750; found 599.9789.

#### *N*-(3-(Dibromomethyl)-3-(4-methoxyphenyl)-6-methylimidazo[1,2-*a*]pyridin-2(3*H*)-ylidene)-4-methylbenzenesulfonamide (3m)

A yellow solid (73%). Mp: 148–150 °C ^1^H NMR (500 MHz, chloroform-*d*) *δ* 8.18 (s, 1H), 7.85 (d, *J* = 8.0 Hz, 3H), 7.43 (d, *J* = 9.0 Hz, 1H), 7.16 (d, *J* = 7.9 Hz, 2H), 7.00 (d, *J* = 8.7 Hz, 2H), 6.87 (d, *J* = 8.8 Hz, 2H), 6.55 (s, 1H), 3.79 (s, 3H), 2.36 (d, *J* = 15.9 Hz, 6H). ^13^C NMR (126 MHz, chloroform-*d*) *δ* 176.49, 164.33, 160.37, 147.39, 142.05, 133.10, 129.50, 128.56, 127.57, 127.06, 126.33, 125.98, 117.17, 114.61, 81.47, 55.42, 45.85, 21.49, 18.03. HRMS: calcd for C_23_H_21_Br_2_N_3_O_3_S [M + H]^+^ 579.9723; found 579.9725.

#### *N*-(3-(4-Chlorophenyl)-3-(dibromomethyl)-6-methylimidazo[1,2-*a*]pyridin-2(3*H*)-ylidene)-4-methylbenzenesulfonamide (3n)

A yellow solid (60%). Mp: 151–153 °C ^1^H NMR (500 MHz, chloroform-*d*) *δ* 8.17 (s, 1H), 7.88 (d, *J* = 8.7 Hz, 1H), 7.84 (d, *J* = 8.2 Hz, 2H), 7.46 (d, *J* = 9.0 Hz, 1H), 7.34 (d, *J* = 8.7 Hz, 2H), 7.17 (d, *J* = 8.0 Hz, 2H), 7.04 (d, *J* = 8.6 Hz, 2H), 6.53 (s, 1H), 2.37 (d, *J* = 15.7 Hz, 6H). ^13^C NMR (126 MHz, chloroform-*d*) *δ* 175.57, 164.54, 147.66, 142.21, 132.80, 132.67, 131.04, 129.45, 129.01, 128.96, 128.60, 128.08, 127.61, 127.08, 120.99, 117.32, 117.24, 81.19, 45.25, 21.51, 18.05. HRMS: calcd for C_22_H_18_Br_2_ClN_3_O_2_S [M + H]^+^ 583.9226; found 583.9229.

#### *N*-(3-(Dibromomethyl)-3-(3-fluorophenyl)-6-methylimidazo[1,2-*a*]pyridin-2(3*H*)-ylidene)-4-methylbenzenesulfonamide (3o)

A yellow solid (66%). Mp: 127–129 °C ^1^H NMR (500 MHz, chloroform-*d*) *δ* 8.16 (s, 1H), 7.90 (d, *J* = 8.8 Hz, 1H), 7.80 (d, *J* = 8.1 Hz, 2H), 7.42 (d, *J* = 9.0 Hz, 1H), 7.34–7.29 (m, 1H), 7.12 (d, *J* = 8.0 Hz, 2H), 7.04 (t, *J* = 8.0 Hz, 1H), 6.82 (t, *J* = 7.0 Hz, 2H), 6.48 (s, 1H), 2.32 (d, *J* = 16.8 Hz, 6H). ^13^C NMR (126 MHz, chloroform-*d*) *δ* 174.68, 163.35, 147.50, 141.36, 138.09, 135.55, 132.30, 130.27, 130.20, 128.51, 127.78, 126.64, 125.28, 120.76, 116.12, 112.81, 112.61, 80.55, 44.50, 20.65, 20.59, 17.13. HRMS: calcd for C_22_H_18_Br_2_FN_3_O_2_S [M + H]^+^ 567.9523; found 567.9528.

#### *N*-(6-Chloro-3-(dibromomethyl)-3-phenylimidazo[1,2-*a*]pyridin-2(3*H*)-ylidene)-4-methylbenzenesulfonamide (3p)

A yellow solid (58%). Mp: 144–146 °C ^1^H NMR (500 MHz, chloroform-*d*) *δ* 8.40 (s, 1H), 7.99 (d, *J* = 9.2 Hz, 1H), 7.84 (d, *J* = 8.1 Hz, 2H), 7.59 (d, *J* = 9.5 Hz, 1H), 7.40 (d, *J* = 7.4 Hz, 3H), 7.18 (d, *J* = 7.9 Hz, 2H), 7.09 (d, *J* = 6.9 Hz, 2H), 6.55 (s, 1H), 2.36 (s, 3H). ^13^C NMR (126 MHz, chloroform-*d*) *δ* 175.93, 164.80, 145.90, 142.44, 138.67, 133.58, 132.83, 129.96, 129.63, 129.50, 129.04, 128.71, 127.91, 127.57, 125.38, 118.51, 81.91, 45.09, 21.54. HRMS: calcd for C_21_H_16_Br_2_ClN_3_O_2_S [M + H]^+^ 569.9070; found 569.9075.

#### *N*-(6-Chloro-3-(Dibromomethyl)-3-(4-ethylphenyl)imidazo[1,2-*a*]pyridin-2(3*H*)-ylidene)-4-methylbenzenesulfonamide (3q)

A yellow solid (61%). Mp: 149–151 °C ^1^H NMR (500 MHz, chloroform-*d*) *δ* 8.41 (s, 1H), 7.97–7.93 (m, 1H), 7.86 (d, *J* = 8.1 Hz, 2H), 7.53 (d, *J* = 9.5 Hz, 1H), 7.21 (dd, *J* = 16.9, 8.1 Hz, 4H), 7.00 (d, *J* = 8.2 Hz, 2H), 6.55 (s, 1H), 2.67 (q, *J* = 7.6 Hz, 2H), 2.38 (s, 3H), 1.24 (d, *J* = 7.6 Hz, 3H). ^13^C NMR (126 MHz, chloroform-*d*) *δ* 176.21, 164.75, 146.36, 145.80, 142.36, 132.90, 130.83, 129.55, 128.95, 128.67, 128.50, 127.95, 127.60, 125.36, 121.33, 118.44, 81.91, 45.22, 28.39, 21.53, 15.12. HRMS: calcd for C_23_H_20_Br_2_ClN_3_O_2_S [M + H]^+^ 597.93834; found 597.93830.

#### *N*-(6-Chloro-3-(dibromomethyl)-3-(*m*-tolyl)imidazo[1,2-*a*]pyridin-2(3*H*)-ylidene)-4-methylbenzenesulfonamide (3r)

A yellow solid (57%). Mp: 183–185 °C ^1^H NMR (500 MHz, chloroform-*d*) *δ* 8.40 (s, 1H), 7.99 (d, *J* = 9.3 Hz, 1H), 7.84 (d, *J* = 8.0 Hz, 2H), 7.61 (d, *J* = 9.5 Hz, 1H), 7.28–7.26 (m, 1H), 7.20 (dd, *J* = 16.1, 7.7 Hz, 3H), 6.88 (s, 1H), 6.84 (d, *J* = 7.7 Hz, 1H), 6.54 (s, 1H), 2.36 (d, *J* = 13.9 Hz, 6H). ^13^C NMR (126 MHz, chloroform-*d*) *δ* 176.01, 164.77, 146.12, 142.40, 139.46, 138.80, 133.51, 132.79, 130.75, 129.31, 128.69, 127.50, 125.86, 122.51, 121.41, 118.60, 81.91, 45.09, 21.63, 21.54. HRMS: calcd for C_22_H_18_Br_2_ClN_3_O_2_S [M + H]^+^ 583.9226; found 583.9231.

#### *N*-(6-Chloro-3-(dibromomethyl)-3-(3-methoxyphenyl)imidazo[1,2-*a*]pyridin-2(3*H*)-ylidene)-4-methylbenzenesulfonamide (3s)

A yellow solid (62%). Mp: 145–147 °C ^1^H NMR (500 MHz, chloroform-*d*) *δ* 8.42 (s, 1H), 7.97 (d, *J* = 8.9 Hz, 1H), 7.86 (d, *J* = 8.1 Hz, 2H), 7.57 (d, *J* = 9.5 Hz, 1H), 7.32 (d, *J* = 8.0 Hz, 1H), 7.19 (d, *J* = 7.9 Hz, 2H), 6.94 (d, *J* = 8.6 Hz, 1H), 6.64 (d, *J* = 8.0 Hz, 2H), 6.51 (s, 1H), 3.77 (s, 3H), 2.37 (s, 3H). ^13^C NMR (126 MHz, chloroform-*d*) *δ* 175.79, 164.72, 160.20, 145.92, 142.47, 138.73, 134.88, 132.86, 130.54, 128.73, 127.53, 121.32, 118.47, 117.45, 114.84, 112.03, 81.74, 55.51, 45.03, 21.52. HRMS: calcd for C_22_H_18_Br_2_ClN_3_O_3_S [M + H]^+^ 597.9196; found 597.9191.

#### *N*-(6-Chloro-3-(dibromomethyl)-3-(4-methoxyphenyl)imidazo[1,2-*a*]pyridin-2(3*H*)-ylidene)-4-methylbenzenesulfonamide (3t)

A yellow solid (61%). Mp: 134–136 °C ^1^H NMR (500 MHz, chloroform-*d*) *δ* 8.40 (s, 1H), 7.96 (d, *J* = 8.9 Hz, 1H), 7.85 (d, *J* = 7.9 Hz, 2H), 7.55 (d, *J* = 9.0 Hz, 1H), 7.19 (d, *J* = 7.8 Hz, 2H), 7.02 (d, *J* = 8.6 Hz, 2H), 6.90 (d, *J* = 8.4 Hz, 2H), 6.52 (s, 1H), 3.81 (s, 3H), 2.37 (s, 3H). ^13^C NMR (126 MHz, chloroform-*d*) *δ* 176.48, 164.59, 160.57, 145.97, 142.50, 132.87, 131.08, 128.75, 127.53, 126.87, 125.38, 121.49, 118.48, 114.83, 81.72, 55.49, 45.35, 21.54. HRMS: calcd for C_22_H_18_Br_2_ClN_3_O_3_S [M + H]^+^ 597.9196; found 597.9192.

#### *N*-(6-Chloro-3-(3-chlorophenyl)-3-(dibromomethyl)imidazo[1,2-*a*]pyridin-2(3*H*)-ylidene)-4-methylbenzenesulfonamide (3u)

A light yellow solid (56%). Mp: 231–233 °C ^1^H NMR (500 MHz, chloroform-*d*) *δ* 8.41 (s, 1H), 7.98 (d, *J* = 9.4 Hz, 1H), 7.85 (d, *J* = 8.1 Hz, 2H), 7.57 (d, *J* = 9.5 Hz, 1H), 7.42 (d, *J* = 8.1 Hz, 1H), 7.37 (t, *J* = 8.2 Hz, 1H), 7.22 (d, *J* = 7.9 Hz, 2H), 7.06 (s, 2H), 6.49 (s, 1H), 2.39 (s, 3H). ^13^C NMR (126 MHz, chloroform-*d*) *δ* 175.16, 164.92, 145.94, 142.60, 135.59, 135.47, 132.50, 130.78, 130.26, 128.77, 127.56, 125.52, 123.75, 121.48, 118.62, 81.23, 44.59, 21.54. HRMS: calcd for C_21_H_15_Br_2_Cl_2_N_3_O_2_S [M + H]^+^ 603.8679; found 603.8678.

#### *N*-(6-Chloro-3-(dibromomethyl)-3-(3-fluorophenyl)imidazo[1,2-*a*]pyridin-2(3*H*)-ylidene)-4-methylbenzenesulfonamide (3v)

A yellow solid (58%). Mp: 145–147 °C ^1^H NMR (500 MHz, chloroform-*d*) *δ* 8.39 (s, 1H), 8.02 (d, *J* = 9.3 Hz, 1H), 7.83 (d, *J* = 8.1 Hz, 2H), 7.62 (d, *J* = 9.4 Hz, 1H), 7.37 (q, *J* = 7.8 Hz, 1H), 7.18 (d, *J* = 7.9 Hz, 2H), 7.11 (t, *J* = 6.8 Hz, 1H), 6.87 (t, *J* = 9.8 Hz, 2H), 6.48 (s, 1H), 2.35 (s, 3H). ^13^C NMR (126 MHz, chloroform-*d*) *δ* 175.31, 164.81, 163.89, 161.91, 146.28, 142.63, 138.49, 135.72, 132.54, 131.30, 128.77, 127.55, 121.72, 121.18, 118.70, 117.08, 113.18, 112.98, 81.31, 44.61, 21.54. HRMS: calcd for C_21_H_15_Br_2_ClFN_3_O_2_S [M + H]^+^ 585.8997; found 585.8999.

#### *N*-(3-(Dibromomethyl)-3-phenylimidazo[1,2-*a*]pyridin-2(3*H*)-ylidene)methanesulfonamide (3w)

A yellow solid (61%). Mp: 256–258 °C ^1^H NMR (500 MHz, chloroform-*d*) *δ* 8.50 (d, *J* = 6.4 Hz, 1H), 8.09 (t, *J* = 7.5 Hz, 1H), 7.59 (d, *J* = 8.8 Hz, 1H), 7.41 (d, *J* = 5.7 Hz, 3H), 7.15 (d, *J* = 5.6 Hz, 3H), 6.66 (s, 1H), 3.15 (s, 3H). ^13^C NMR (126 MHz, chloroform-*d*) *δ* 176.80, 165.98, 145.12, 135.43, 129.89, 129.46, 125.50, 117.58, 114.24, 81.37, 45.89, 39.93. HRMS: calcd for C_15_H_13_Br_2_N_3_O_2_S [M + H]^+^ 457.9167; found 457.9168.

#### *N*-(3-(Dibromomethyl)-3-phenylimidazo[1,2-*a*]pyridin-2(3*H*)-ylidene)benzenesulfonamide (3x)

A yellow solid (49%). Mp: 139–141 °C ^1^H NMR (500 MHz, chloroform-*d*) *δ* 8.43 (d, *J* = 4.3 Hz, 1H), 7.98 (d, *J* = 7.6 Hz, 2H), 7.60 (d, *J* = 5.9 Hz, 5H), 7.54 (s, 1H), 7.46 (d, *J* = 7.6 Hz, 2H), 7.39 (d, *J* = 7.1 Hz, 3H), 7.25 (s, 1H), 7.09 (d, *J* = 6.5 Hz, 3H), 6.60 (s, 1H). ^13^C NMR (126 MHz, chloroform-*d*) *δ* 176.20, 165.73, 145.51, 143.23, 135.47, 131.98, 129.54, 129.28, 128.79, 127.93, 127.42, 126.08, 125.51, 117.46, 114.89, 81.53, 45.33. HRMS: calcd for C_20_H_15_Br_2_N_3_O_2_S [M + Na]^+^ 543.9124; found 543.9128.

#### *N*-(3-(Dibromomethyl)-3-(4-ethylphenyl)imidazo[1,2-*a*]pyridin-2(3*H*)-ylidene)benzenesulfonamide (3y)

A yellow solid (47%). Mp: 147–149 °C ^1^H NMR (500 MHz, chloroform-*d*) *δ* 8.42 (d, *J* = 6.1 Hz, 1H), 7.97 (d, *J* = 7.9 Hz, 2H), 7.49 (d, *J* = 8.4 Hz, 1H), 7.43 (d, *J* = 7.6 Hz, 1H), 7.37 (d, *J* = 7.0 Hz, 2H), 7.18 (d, *J* = 7.5 Hz, 2H), 7.07 (dd, *J* = 14.5, 7.5 Hz, 2H), 6.98 (d, *J* = 6.8 Hz, 2H), 6.58 (s, 1H), 2.65–2.62 (m, 2H), 1.21 (d, *J* = 6.6 Hz, 3H). ^13^C NMR (126 MHz, chloroform-*d*) *δ* 176.48, 165.92, 146.16, 145.06, 135.39, 131.69, 128.81, 128.22, 127.93, 127.63, 125.47, 117.63, 114.34, 81.47, 45.52, 28.39, 15.17. HRMS: calcd for C_22_H_19_Br_2_N_3_O_2_S [M + H]^+^ 549.9617; found 549.9619.

#### *N*-(3-(Dibromomethyl)-6-methyl-3-phenylimidazo[1,2-*a*]pyridin-2(3*H*)-ylidene)benzenesulfonamide (3z)

A light yellow solid (65%). Mp: 121–123 °C ^1^H NMR (500 MHz, chloroform-*d*) *δ* 8.19 (s, 1H), 7.90 (d, *J* = 7.2 Hz, 3H), 7.42 (dd, *J* = 17.0, 7.9 Hz, 3H), 7.37–7.33 (m, 4H), 7.08–7.04 (m, 2H), 6.62 (s, 1H), 2.37 (s, 3H). ^13^C NMR (101 MHz, chloroform-*d*) *δ* 175.93, 163.62, 147.96, 143.26, 141.20, 133.60, 133.05, 131.62, 129.38, 129.01, 128.53, 127.71, 127.00, 125.68, 125.43, 116.49, 81.72, 45.21, 17.63. HRMS: calcd for C_21_H_17_Br_2_N_3_O_2_S [M + H]^+^ 535.9461; found 535.9470.

#### *N*-(6-Chloro-3-(dibromomethyl)-3-phenylimidazo[1,2-*a*]pyridin-2(3*H*)-ylidene)benzenesulfonamide (3ab)

A yellow solid (62%). Mp: 141–143 °C. ^1^H NMR (500 MHz, chloroform-*d*) *δ* 8.41 (s, 1H), 8.00 (d, *J* = 8.4 Hz, 1H), 7.96 (d, *J* = 7.6 Hz, 2H), 7.59 (d, *J* = 9.1 Hz, 1H), 7.49–7.43 (m, 2H), 7.42–7.38 (m, 4H), 7.09 (d, *J* = 6.9 Hz, 2H), 6.56 (s, 1H). ^13^C NMR (101 MHz, chloroform-*d*) *δ* 176.05, 164.62, 145.97, 141.20, 133.32, 132.80, 131.89, 129.93, 129.45, 129.00, 128.02, 127.80, 127.41, 125.27, 118.40, 81.93, 44.82. HRMS: calcd for C_20_H_14_Br_2_ClN_3_O_2_S [M + Na]^+^ 577.8733; found 577.8738.

#### *N*-(6-Chloro-3-(dibromomethyl)-3-(4-ethylphenyl)imidazo[1,2-*a*]pyridin-2(3*H*)-ylidene)benzenesulfonamide (3ac)

A yellow solid (46%). Mp: 135–137 °C ^1^H NMR (500 MHz, chloroform-*d*) *δ* 8.40 (s, 1H), 7.94 (d, *J* = 6.6 Hz, 2H), 7.61–7.51 (m, 2H), 7.48–7.43 (m, 2H), 7.38 (s, 2H), 7.23–7.19 (m, 2H), 7.00–6.97 (m, 1H), 6.57 (s, 1H), 2.68–2.61 (m, 2H), 1.22 (d, *J* = 6.6 Hz, 3H). ^13^C NMR (126 MHz, chloroform-*d*) *δ* 176.45, 164.67, 146.47, 146.03, 141.35, 133.03, 132.38, 131.99, 130.68, 129.54, 129.04, 129.00, 128.14, 127.53, 126.35, 125.42, 118.49, 82.12, 45.06, 28.42, 15.16. HRMS: calcd for C_22_H_18_Br_2_ClN_3_O_2_S [M + H]^+^ 583.9226; found 583.9229.

#### *N*-(6-Chloro-3-(4-chlorophenyl)-3-(dibromomethyl)imidazo[1,2-*a*]pyridin-2(3*H*)-ylidene)benzenesulfonamide (3ad)

A yellow solid (65%). Mp: 160–162 °C ^1^H NMR (500 MHz, chloroform-*d*) *δ* 8.39 (s, 1H), 7.96 (d, *J* = 7.7 Hz, 2H), 7.59 (d, *J* = 9.4 Hz, 1H), 7.48 (d, *J* = 7.3 Hz, 1H), 7.41 (t, *J* = 8.2 Hz, 3H), 7.37 (d, *J* = 8.4 Hz, 2H), 7.05 (d, *J* = 8.4 Hz, 2H), 6.54 (s, 1H). ^13^C NMR (101 MHz, chloroform-*d*) *δ* 180.53, 169.20, 151.07, 145.74, 144.01, 140.77, 137.44, 137.31, 136.78, 136.64, 134.30, 132.81, 132.07, 131.67, 126.73, 123.01, 116.37, 86.31, 82.41, 49.37. HRMS: calcd for C_20_H_13_Br_2_Cl_2_N_3_O_2_S [M + H]^+^ 589.8522; found 589.8528.

## Conflicts of interest

There are no conflicts of interest to declare.

## Supplementary Material

RA-009-C9RA09265J-s001

RA-009-C9RA09265J-s002
