# Supplementary material for: Direct intramolecular double cross-dehydrogentive-coupling (CDC) cyclization of N-(2-pyridyl)amidines under metal-free conditions
Source: RSC Adv. 2019 Dec 19;9(72):42172–82. doi: 10.1039/c9ra09265j (PMC9076575; doi:10.1039/c9ra09265j)

Electronic Supplementary Material (ESI) for RSC Advances.
This journal is © The Royal Society of Chemistry 2019

Direct Intramolecular Double Cross-Dehydrogentive-Coupling (CDC) Cyclization of *N*-(2-Pyridyl)amidines under Metal-Free Conditions

Fengping Yi, Chao Fu, Qihui Sun, Huazhen Wei, Genfa, Yu* and Weiyin Yi*

School of Perfume and Aroma Technology, Shanghai Institute of Technology, Shanghai 201418, P. R. China

Supporting information

Table of contents

1. X-Ray Data ………………... S2-S3
2. The reaction equation for the synthesis of *N*-(2-pyridyl)amidines (**1**) S4
3. Another possible mechanism route experienced a base promoted nucleophilic substitution for the formation of **3a**. S4
4. ^{1}H and ^{13}C NMR spectra of the products and impurity in eluent or silica gel……… …………..………………...………………..……………..…..….S5-S34

Crystal structure of the compound 3a. Thermal ellipsoids are shown at 40% probability level.

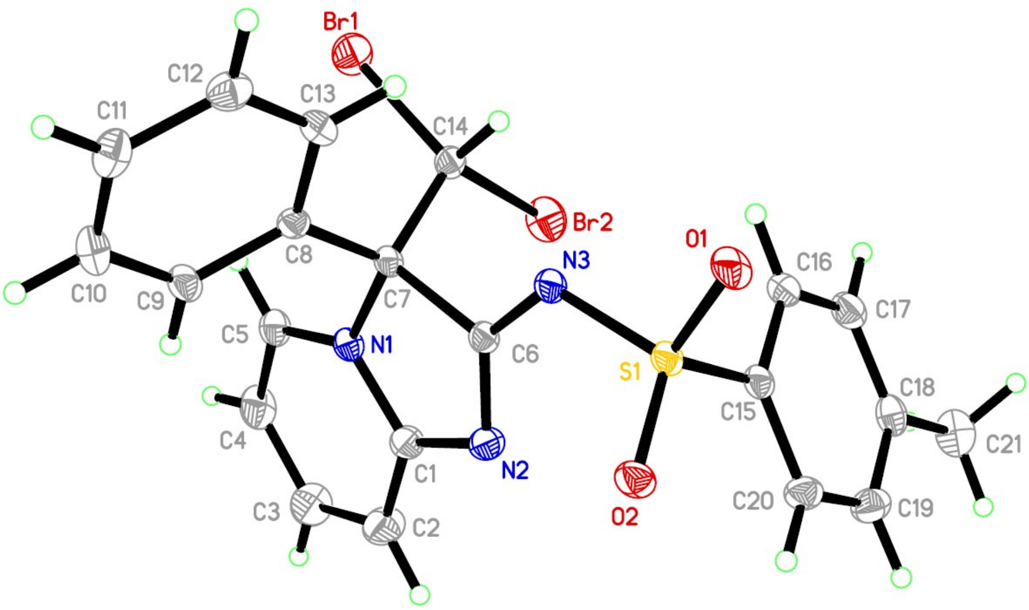

Identification code	t_a
Empirical formula	$C_{21} H_{17} Br_2 N_3 O_2 S$
Formula weight	535.26
Temperature	173(2) K
Wavelength	1.54178 A
Crystal system, space group	Triclinic, P-1
Unit cell dimensions	a = 9.1771(17) A alpha = 81.303(13) deg.
	b = 10.3269(14) A beta = 83.590(15) deg.
	c = 11.3080(12) A gamma = 74.781(11) deg.
Volume	1019.3(3) A^3
Z, Calculated density	2, 1.744 Mg/m^3
Absorption coefficient	6.200 mm^-1
F(000)	532
Crystal size	0.200 x 0.200 x 0.200 mm
Theta range for data collection	3.966 to 68.342 deg.
Limiting indices	-11<=h<=11, -12<=k<=12, -13<=l<=13
Reflections collected / unique	20875 / 3728 [R(int) = 0.0204]
Completeness to theta = 67.679	99.8 %
Refinement method	Full-matrix least-squares on F^2
Data / restraints / parameters	3728 / 0 / 264
Goodness-of-fit on F^2	1.070
Final R indices [I>2sigma(I)]	R1 = 0.0294, wR2 = 0.0955
R indices (all data)	R1 = 0.0309, wR2 = 0.0972
Extinction coefficient	0.0009(3)
Largest diff. peak and hole	0.630 and -0.658 e. A^-3

The reaction equation for the synthesis of *N*-(2-pyridyl)amidines (1)

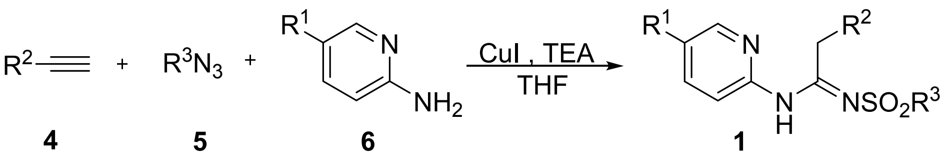

Another possible mechanism route experienced a base promoted nucleophilic substitution for the formation of 3a.

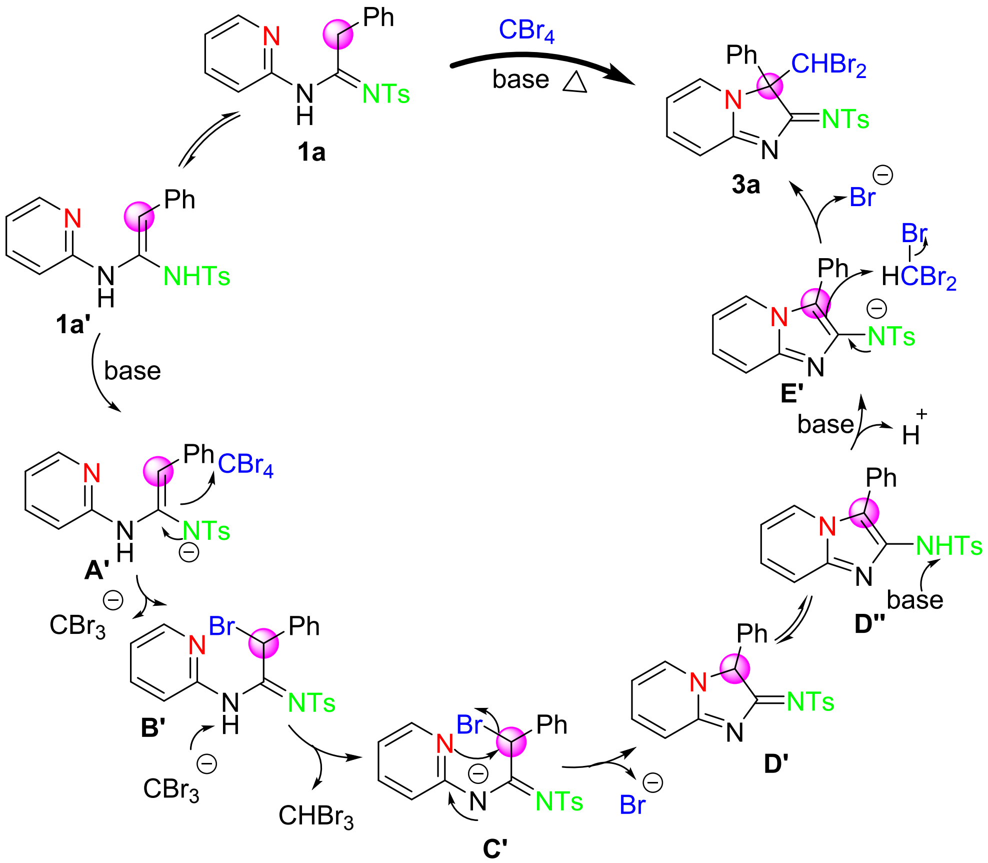

Another tentative mechanism for the transformation of **1a** and CBr_4 into **3a** is also proposed, as depicted above. Initially, substrate **1a** can easily tautomerize into intermediate **1a'** in the presence of the α-hydrogen of the amidine group under such reaction conditions. And then, intermediate **1a'** is transformed into intermediate **A'** under base. Intermediate **A'** reacts rapidly with CBr_4 to afford intermediate **B'** accompanied by the formation of anion CBr_3**.** Subsequently, a base-promoted intramolecular nucleophilic substitution of intermediate **C'**, which is achieved from intermediate **B'** in the presence of anion CBr_3, will occur to provide intermediate **D'**. Intermediate **D'** will tautomerize into intermediate **D''** again. Finally, the desired product **3a** via intermediate **E'** will be afforded by the nucleophilic substitution of intermediate **D''** with $CHBr_3$ under base.

^{1}H and ^{13}C NMR spectra of the products

(Z)-N-(3-(dibromomethyl)-3-phenylimidazo[1,2-a]pyridin-2(3H)-ylidene)-4-methylbenzenesulfonamide（3a）

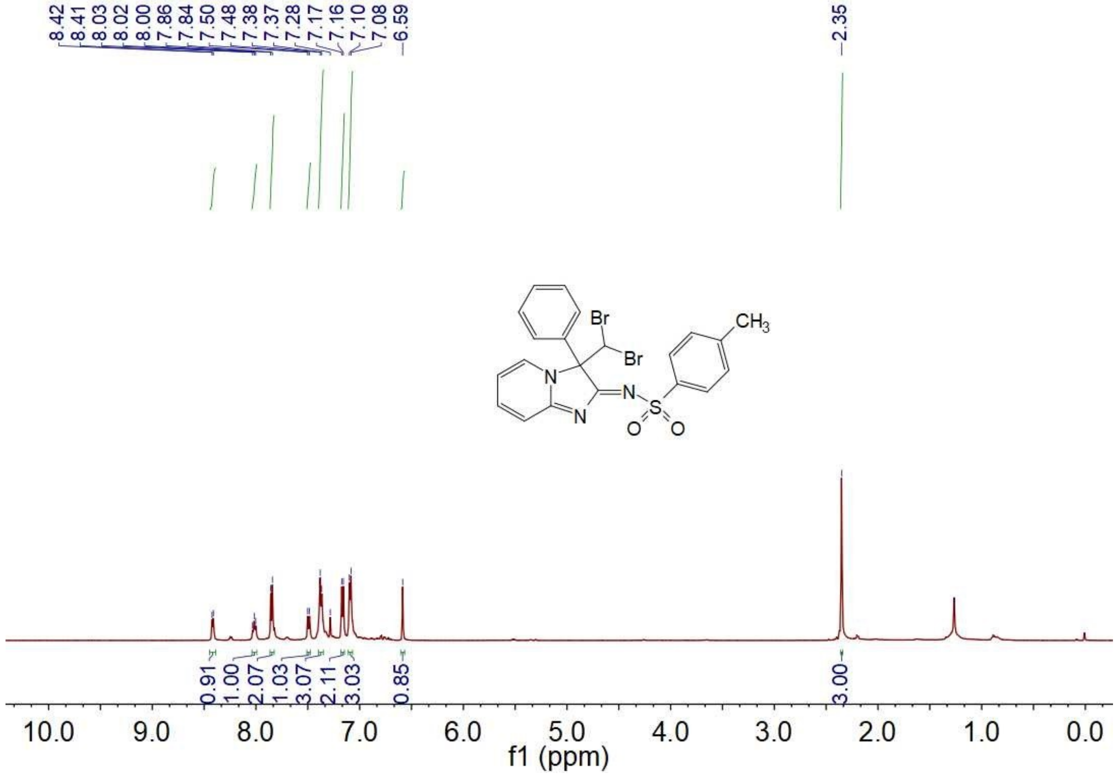

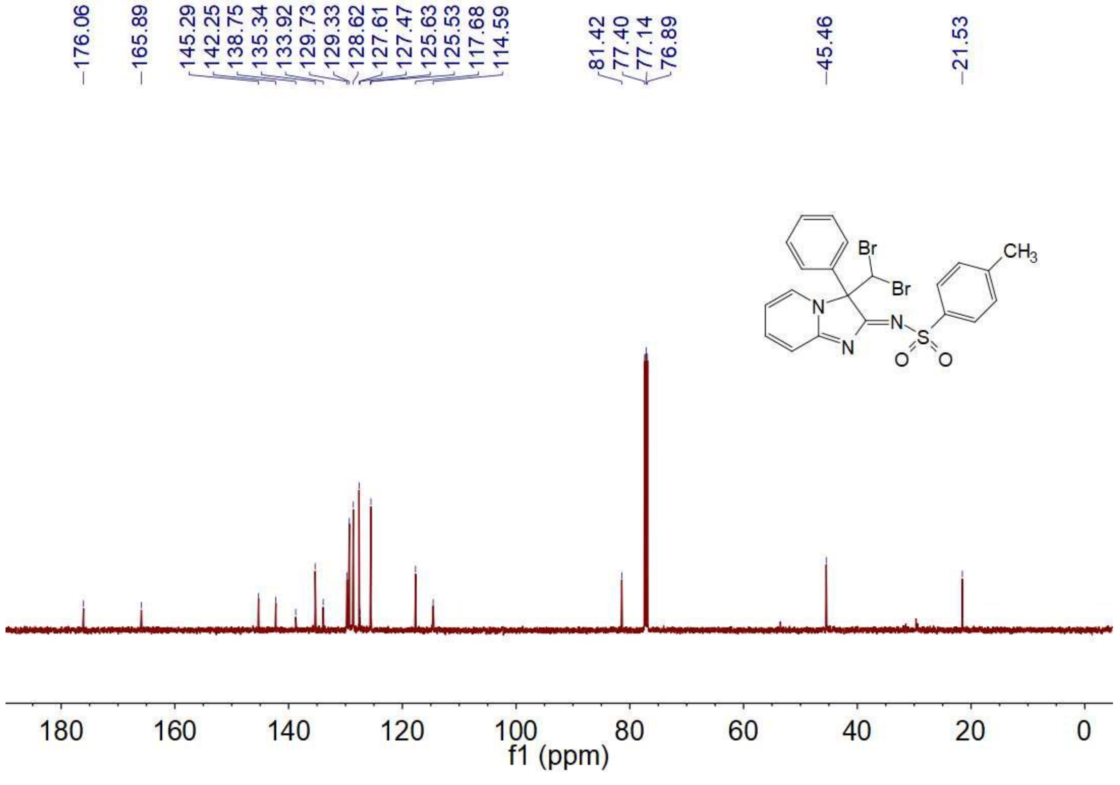

(Z)-N-(3-(dibromomethyl)-3-(4-ethylphenyl)imidazo[1,2-a]pyridin-2(3H)-ylidene)-4-methylbenzenesulfonamide(3b)

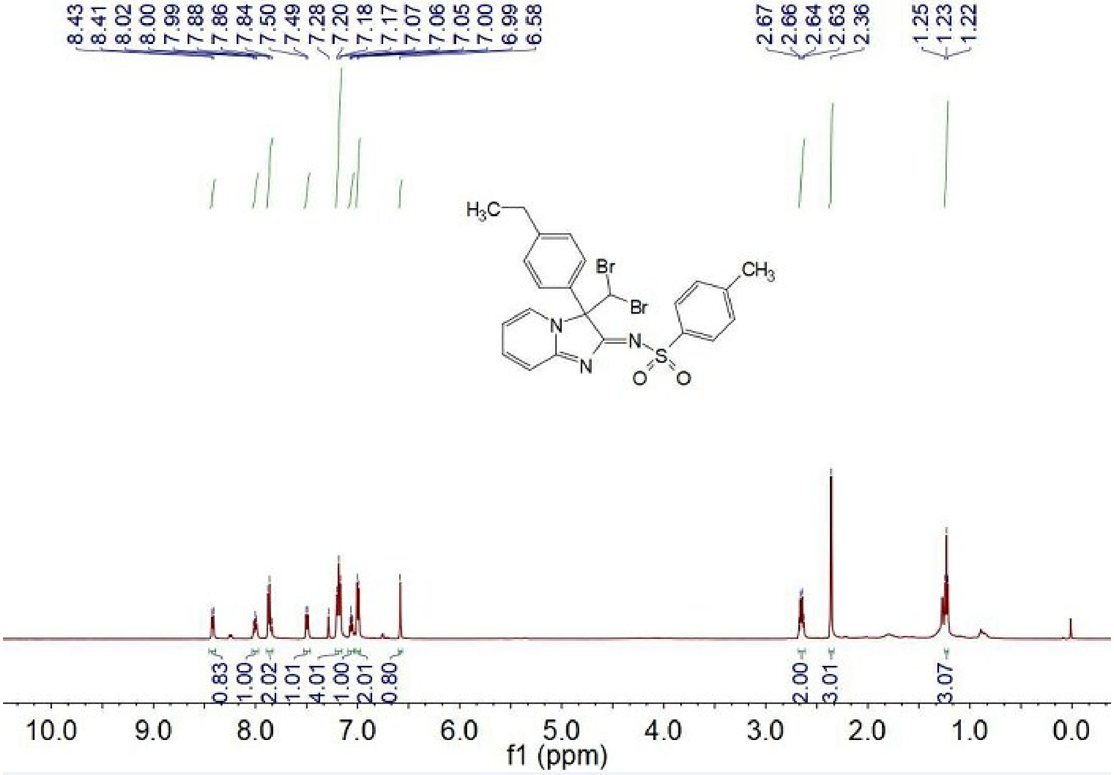

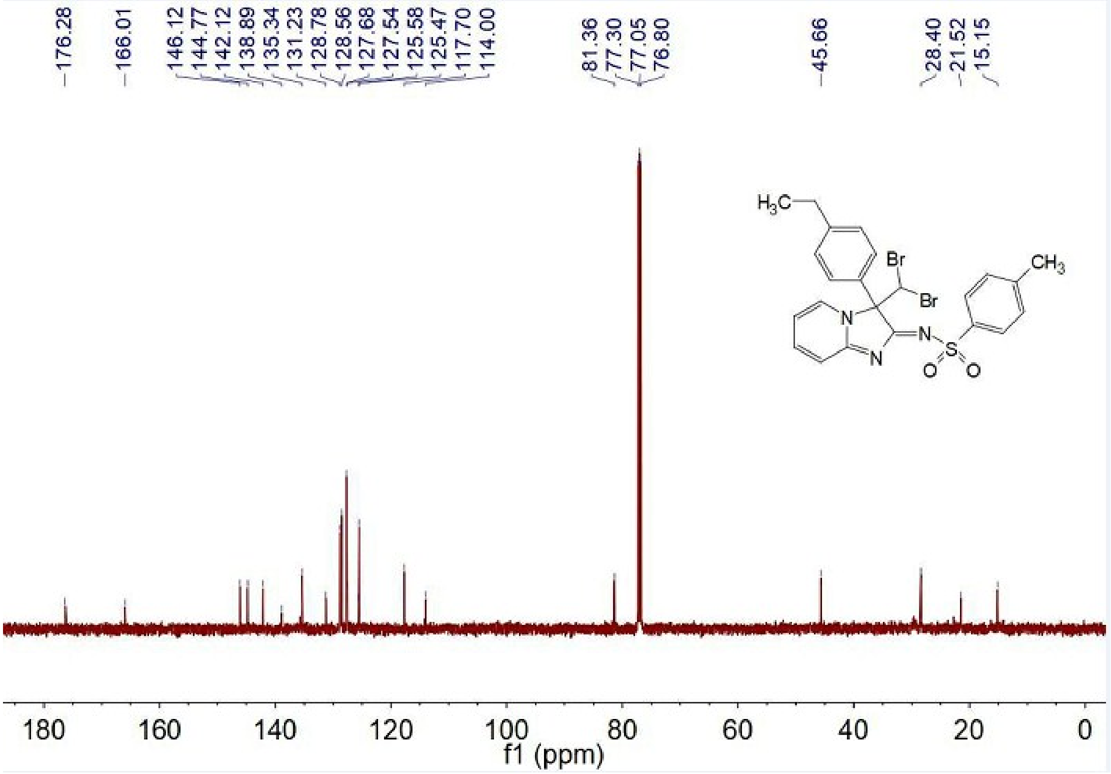

(Z)-N-(3-(dibromomethyl)-3-(p-tolyl)imidazo[1,2-a]pyridin-2(3H)-ylidene)-4-methylbenzenesulfonamide(3c)

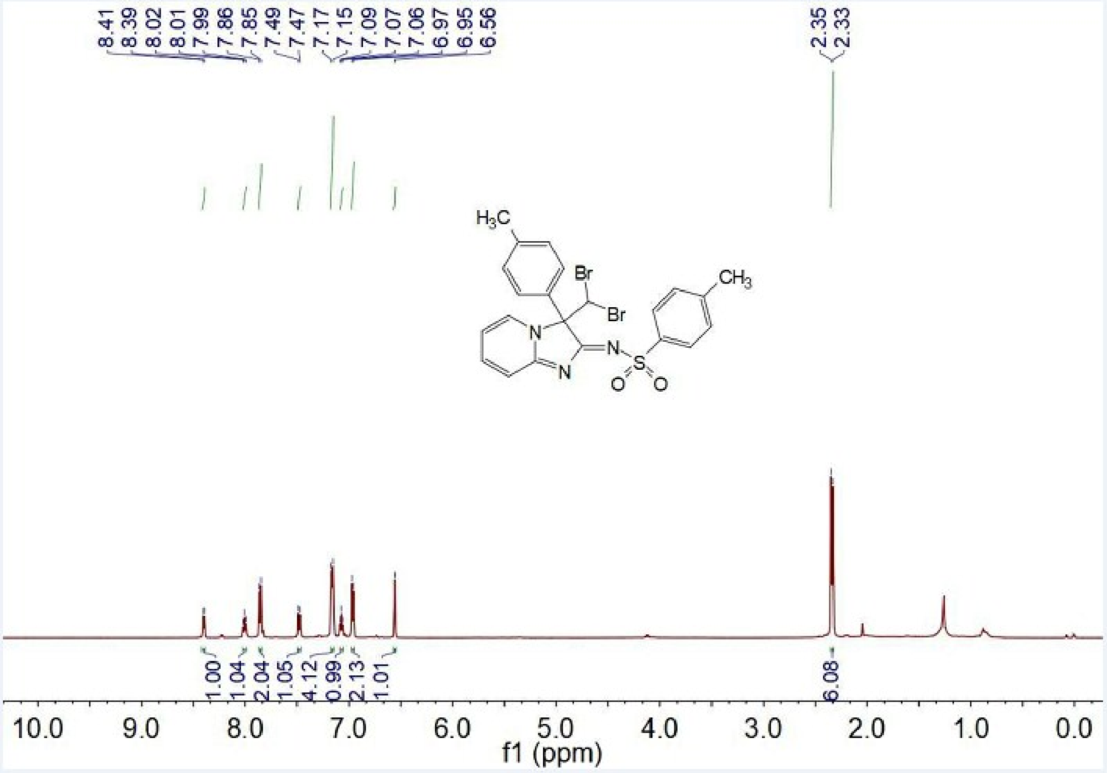

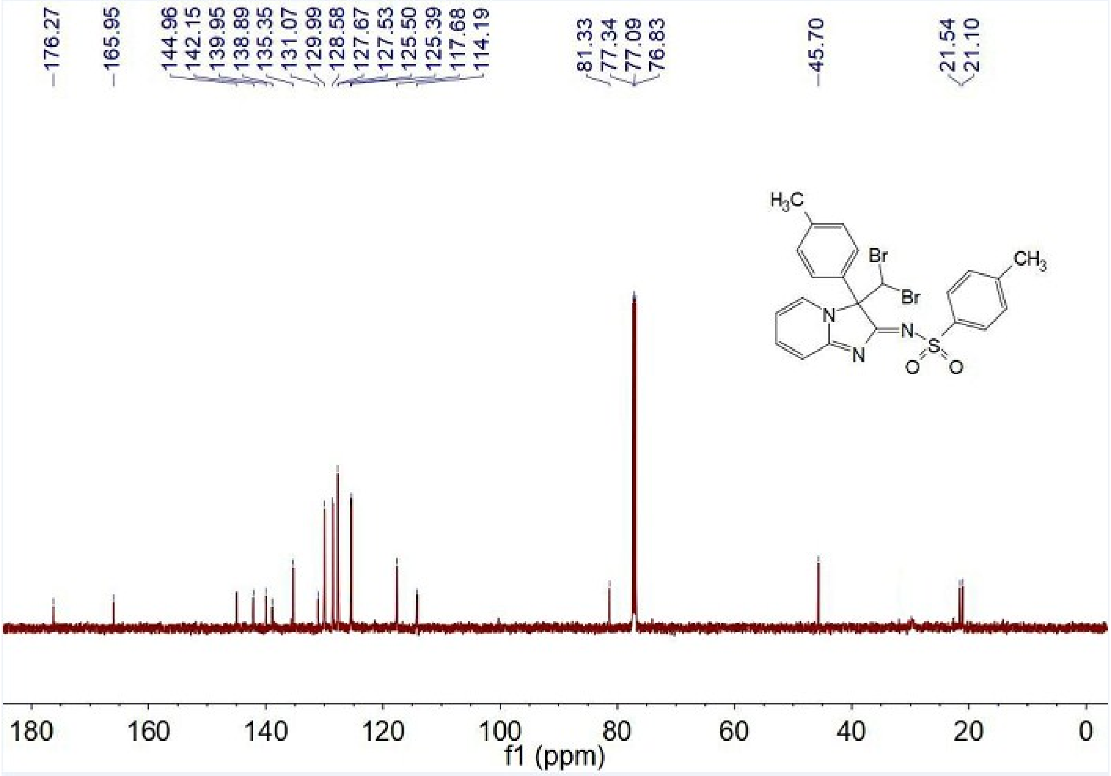

(Z)-N-(3-(dibromomethyl)-3-(m-tolyl)imidazo[1,2-a]pyridin-2(3H)-ylidene)-4-methylbenzenesulfonamide(3d)

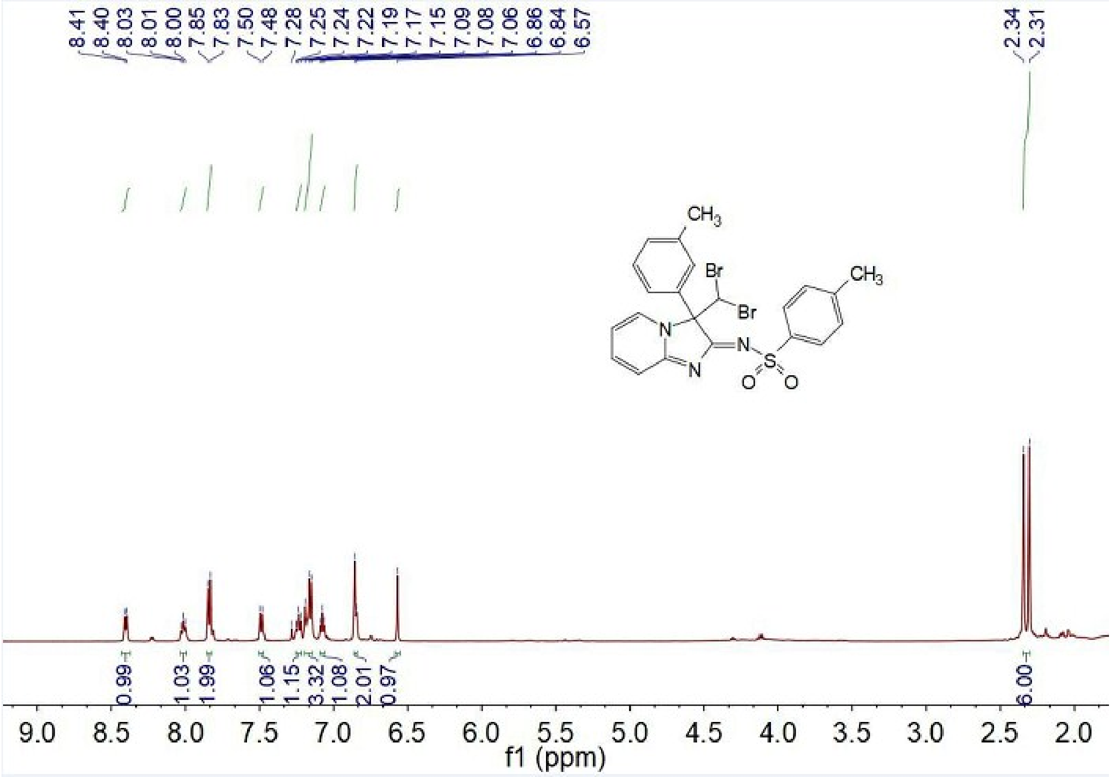

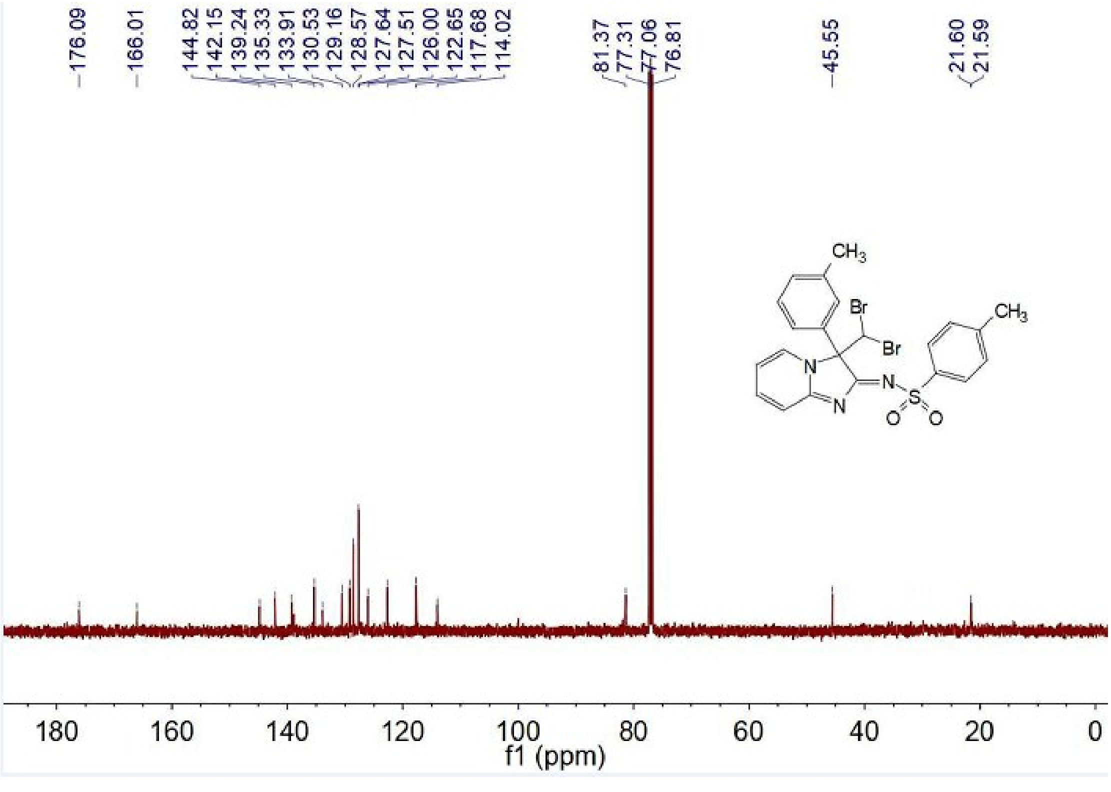

(Z)-N-(3-(dibromomethyl)-3-(4-methoxyphenyl)imidazo[1,2-a]pyridin-2(3H)-ylidene)-4-methylbenzenesulfonamide(3e)

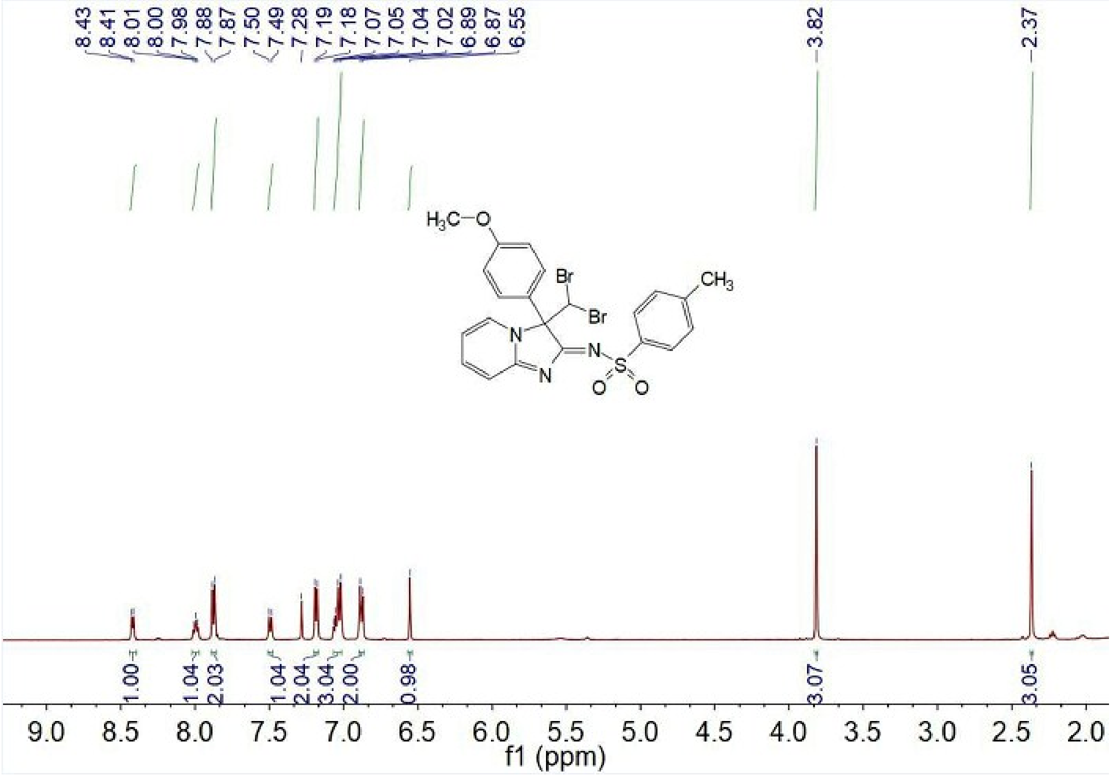

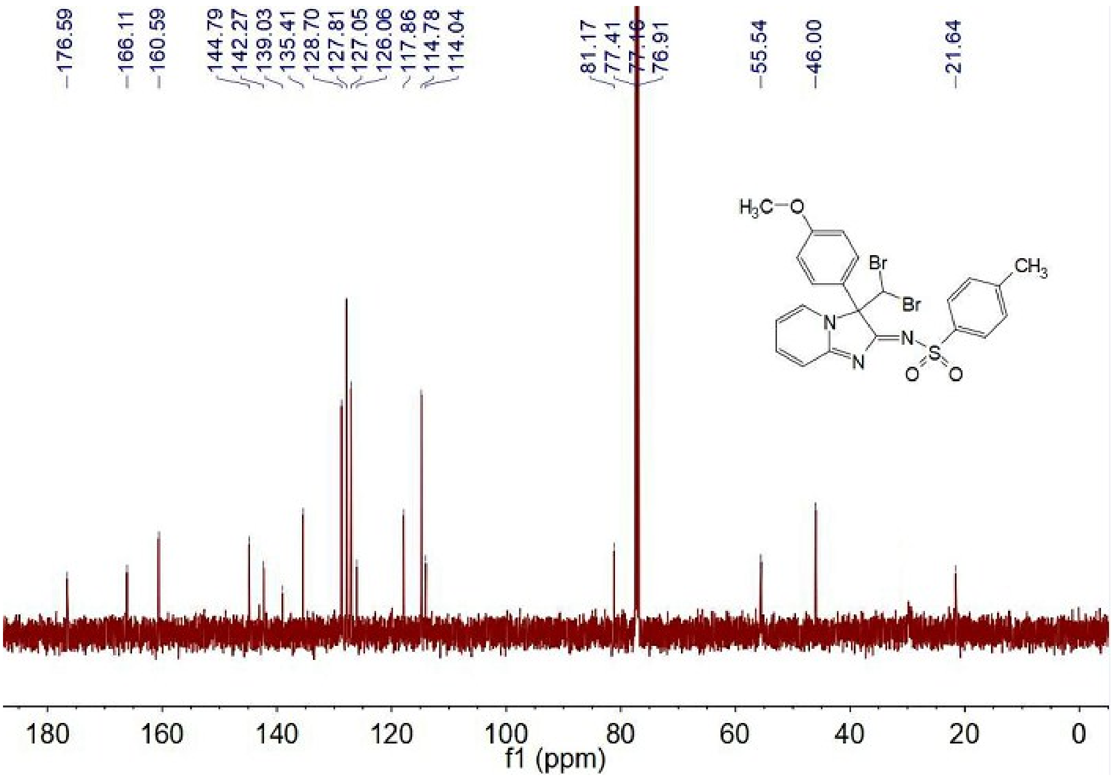

(Z)-N-(3-(4-chlorophenyl)-3-(dibromomethyl)imidazo[1,2-a]pyridin-2(3H)-ylidene)-4-methylbenzenesulfonamide(3f)

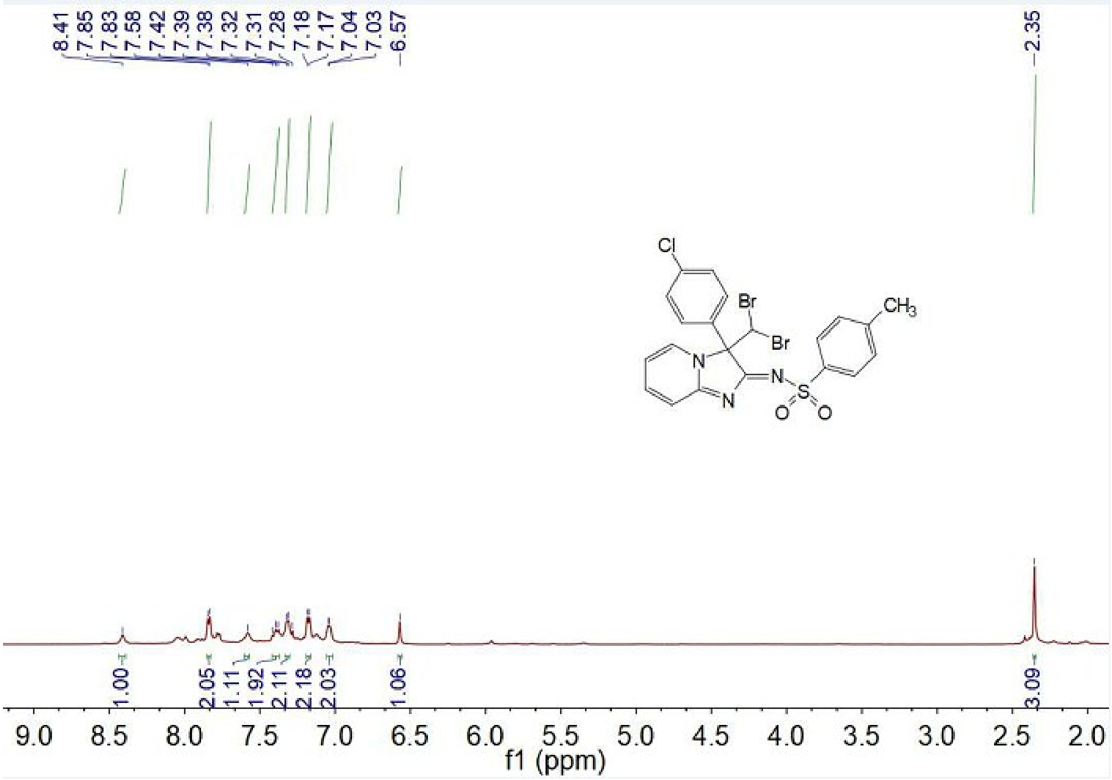

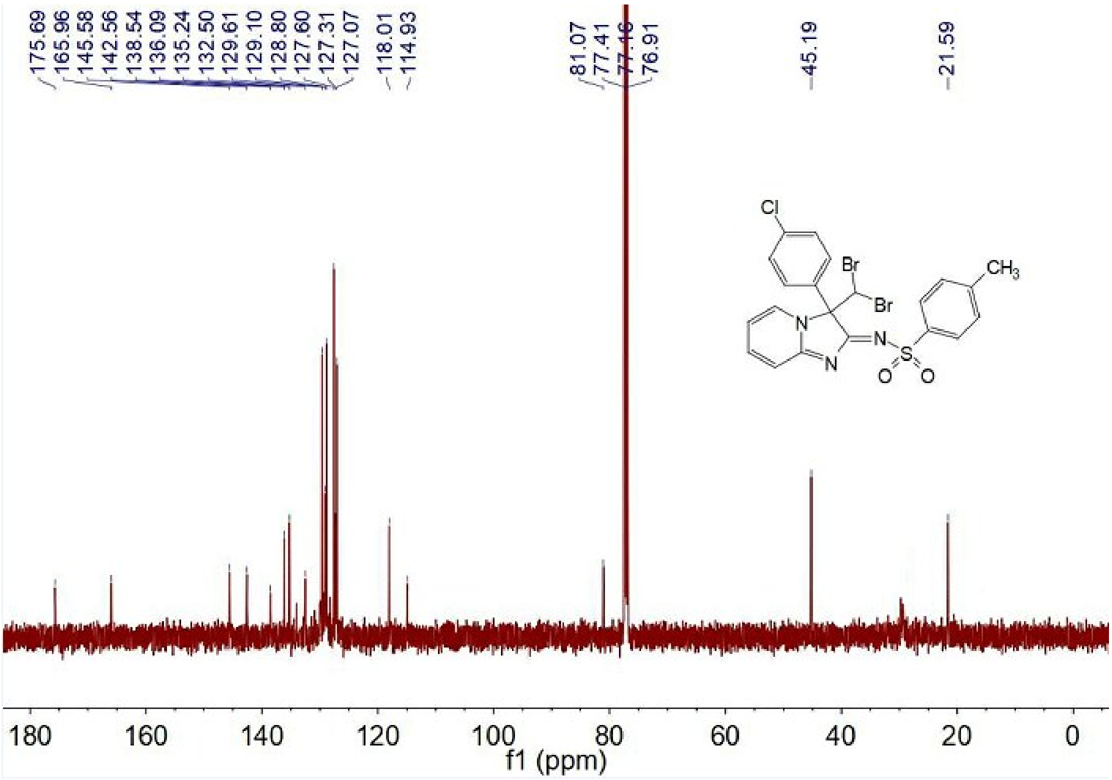

(Z)-N-(3-(3-chlorophenyl)-3-(dibromomethyl)imidazo[1,2-a]pyridin-2(3H)-ylidene)-4-methylbenzenesulfonamide(3g)

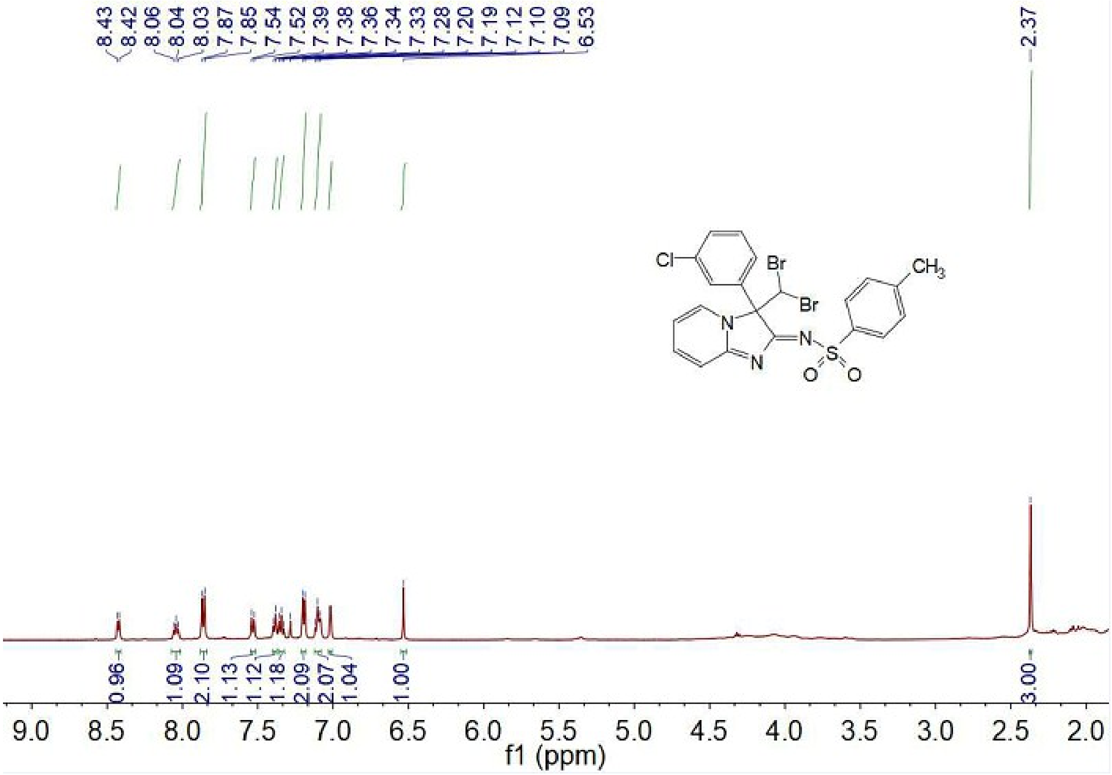

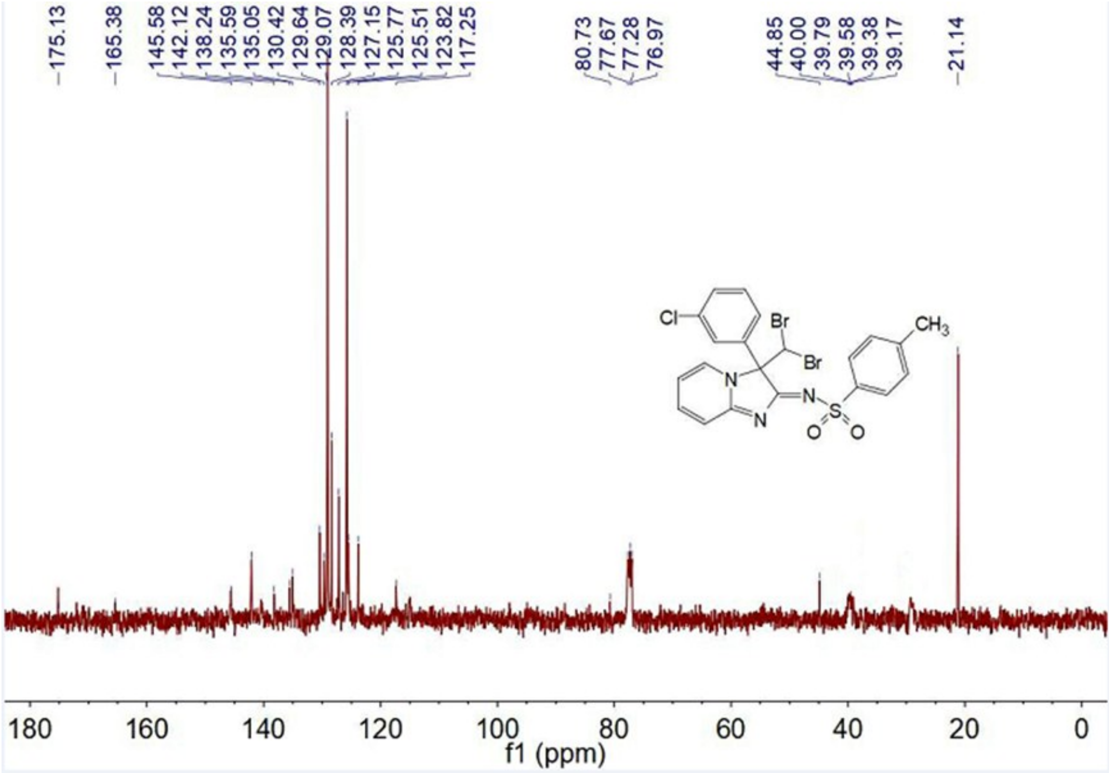

(Z)-N-(3-(dibromomethyl)-3-(4-fluorophenyl)imidazo[1,2-a]pyridin-2(3H)-ylidene)-4-methylbenzenesulfonamide(3h)

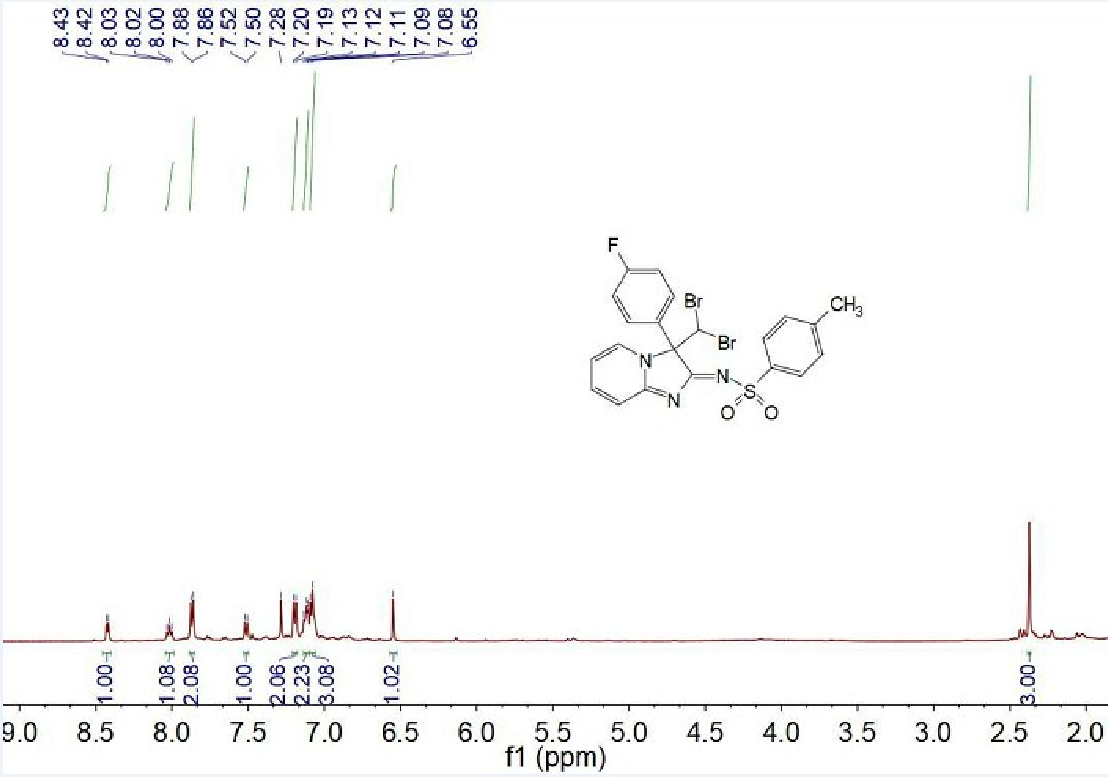

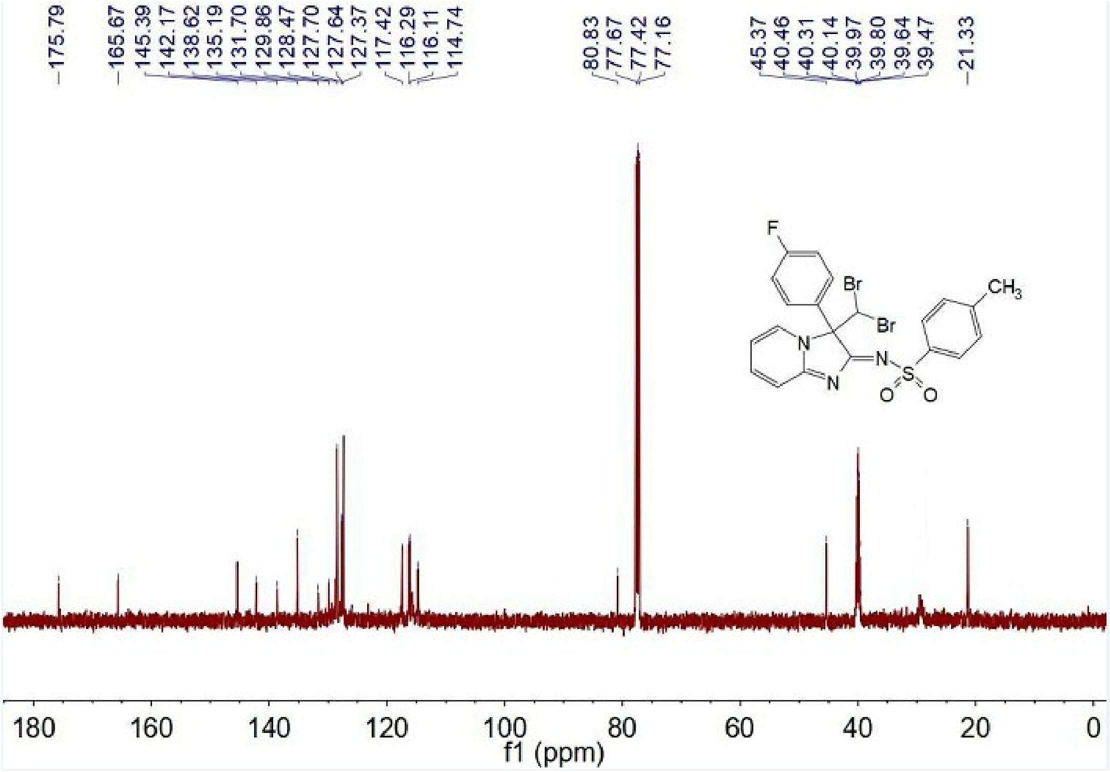

(Z)-N-(3-butyl-3-(dibromomethyl)imidazo[1,2-a]pyridin-2(3H)-ylidene)-4-methylbenzenesulfonamide(3i)

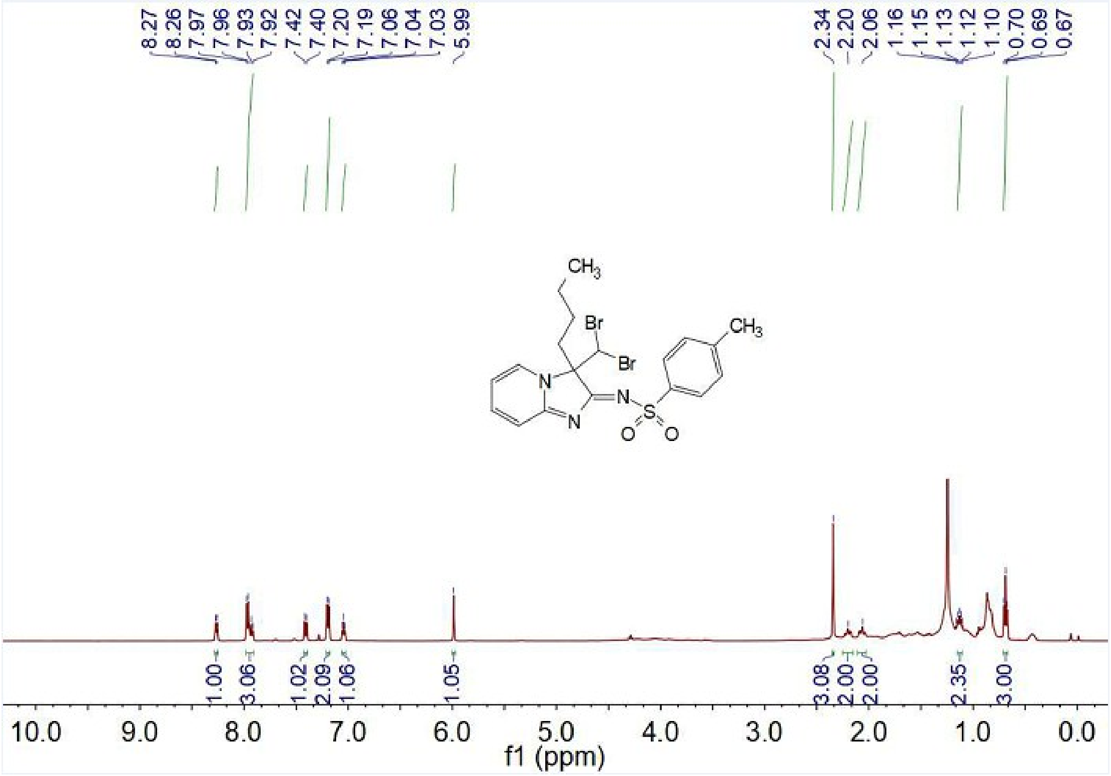

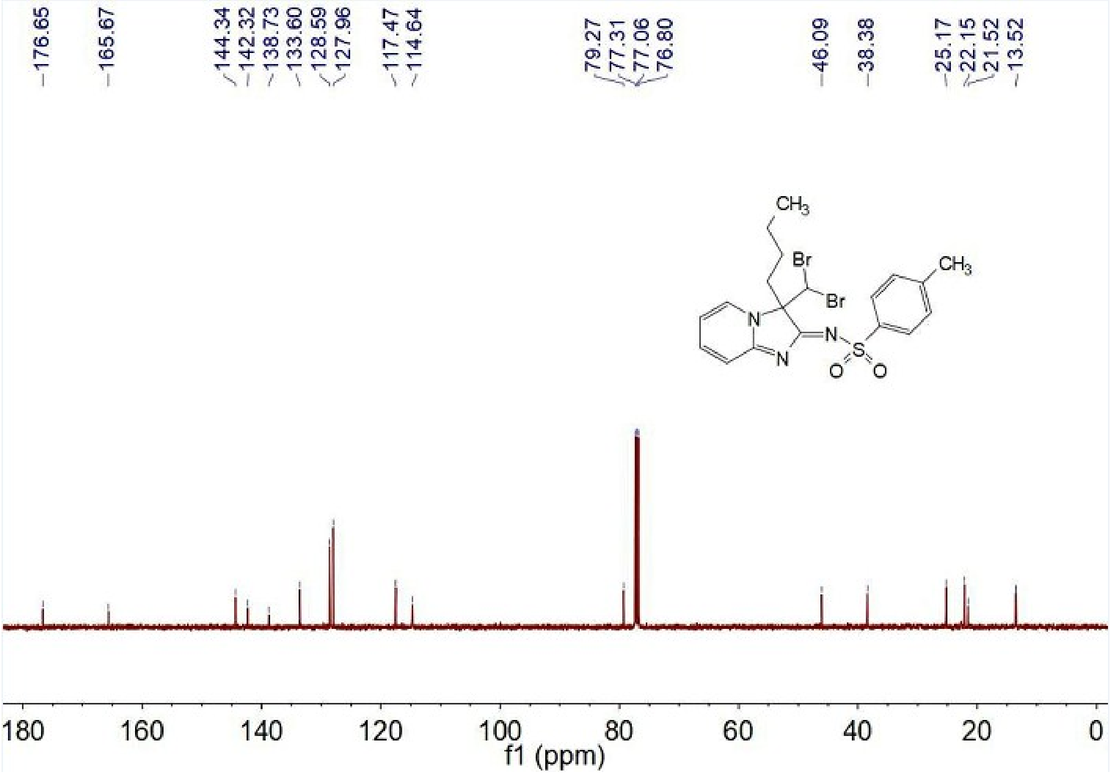

(Z)-N-(3-(dibromomethyl)-6-methyl-3-phenylimidazo[1,2-a]pyridin-2(3H)-ylidene)-4-methylbenzenesulfonamide(3j)

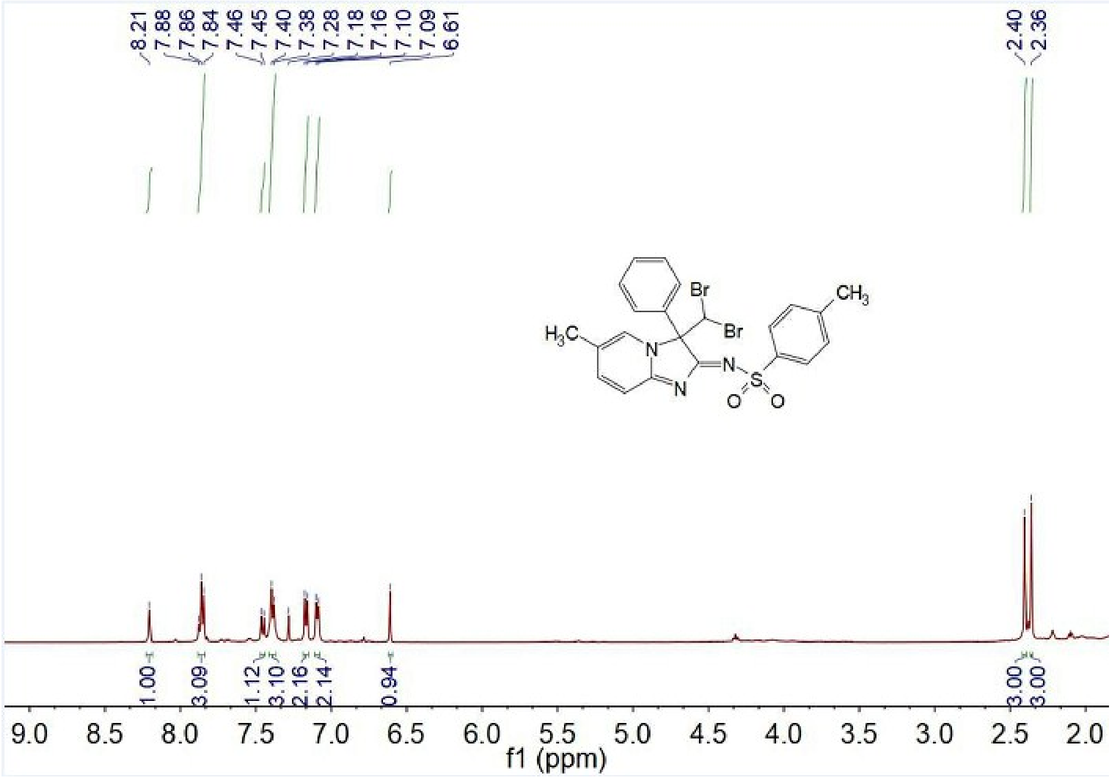

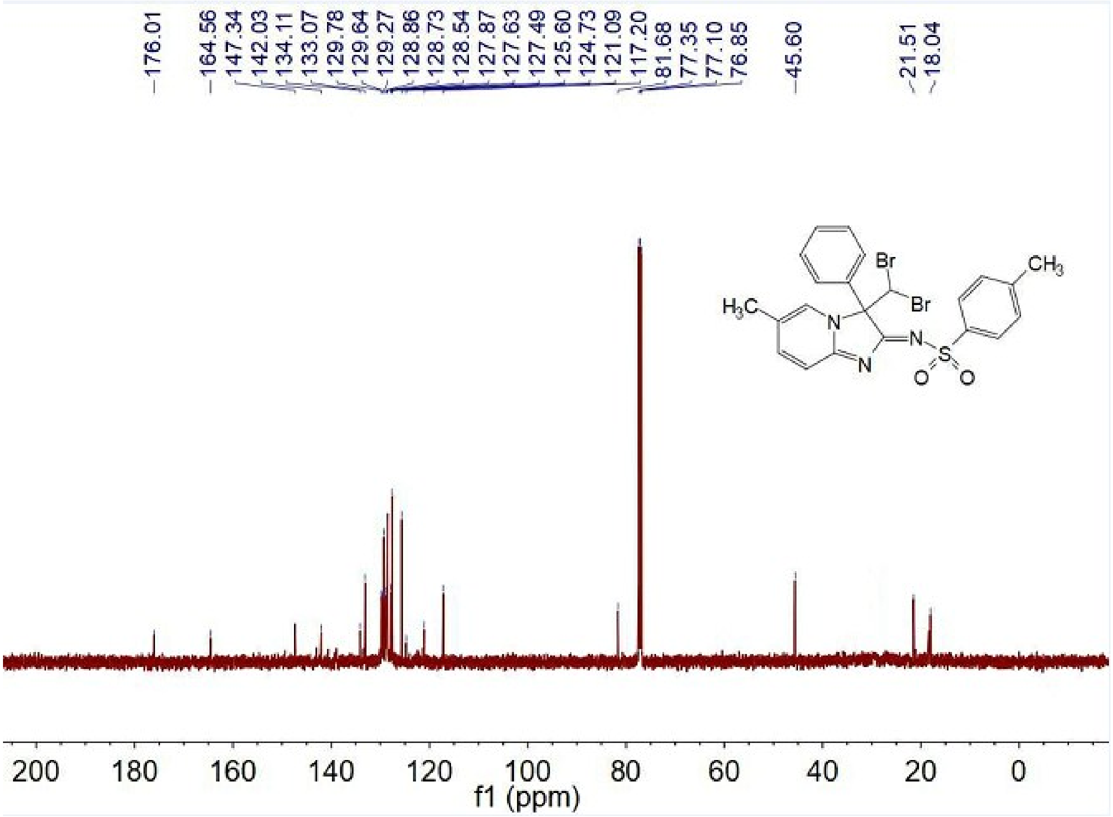

(Z)-N-(3-(dibromomethyl)-6-methyl-3-(p-tolyl)imidazo[1,2-a]pyridin-2(3H)-ylidene)-4-methylbenzenesulfonamide(3k)

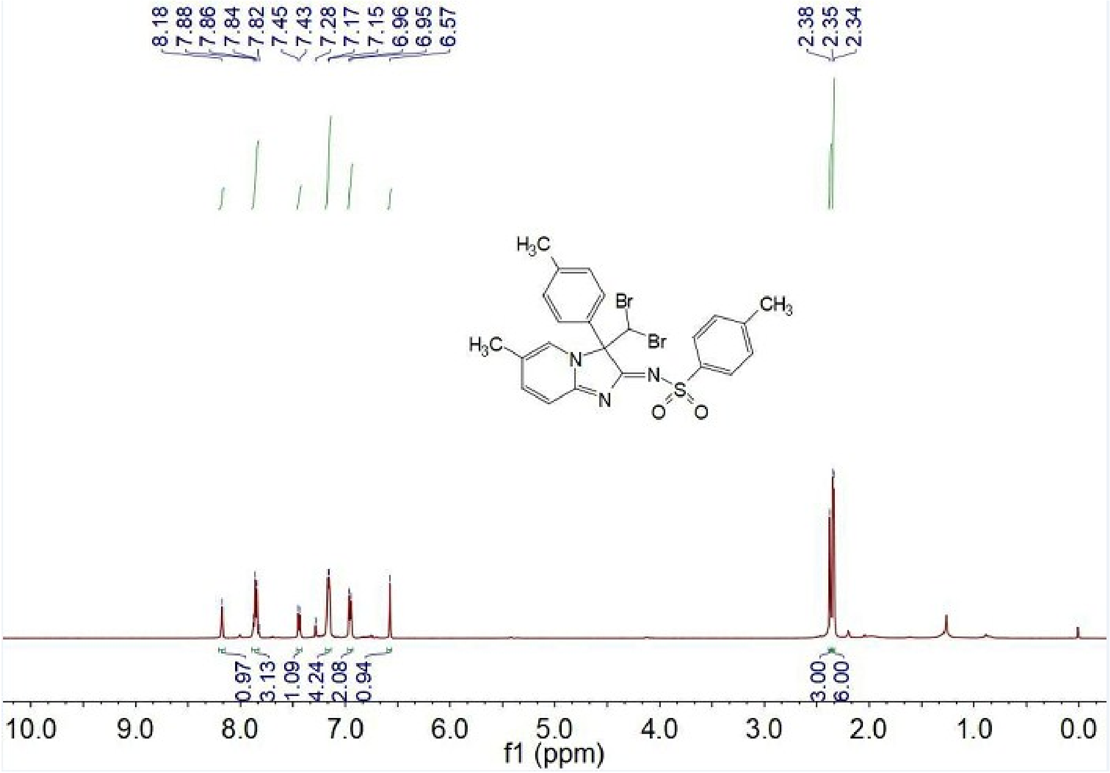

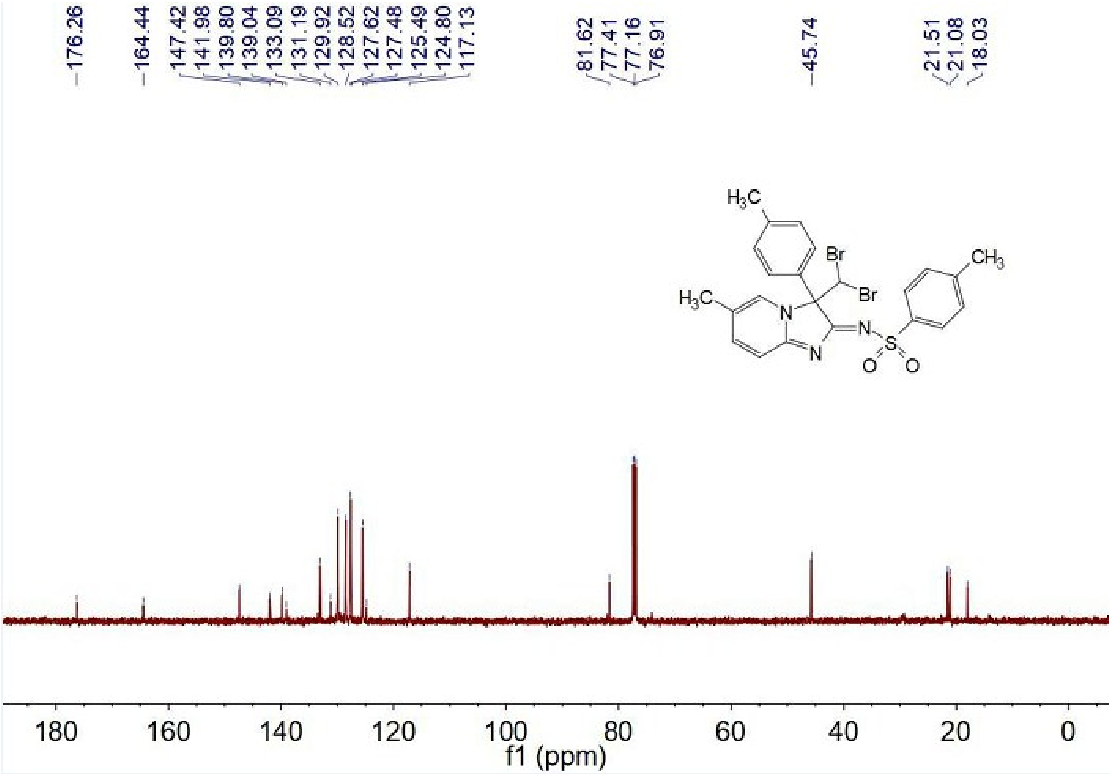

(Z)-N-(3-(dibromomethyl)-3-(4-ethylphenyl)-6-methylimidazo[1,2-a]pyridin-2(3H)-ylidene)-4-methylbenzenesulfonamide(3l)

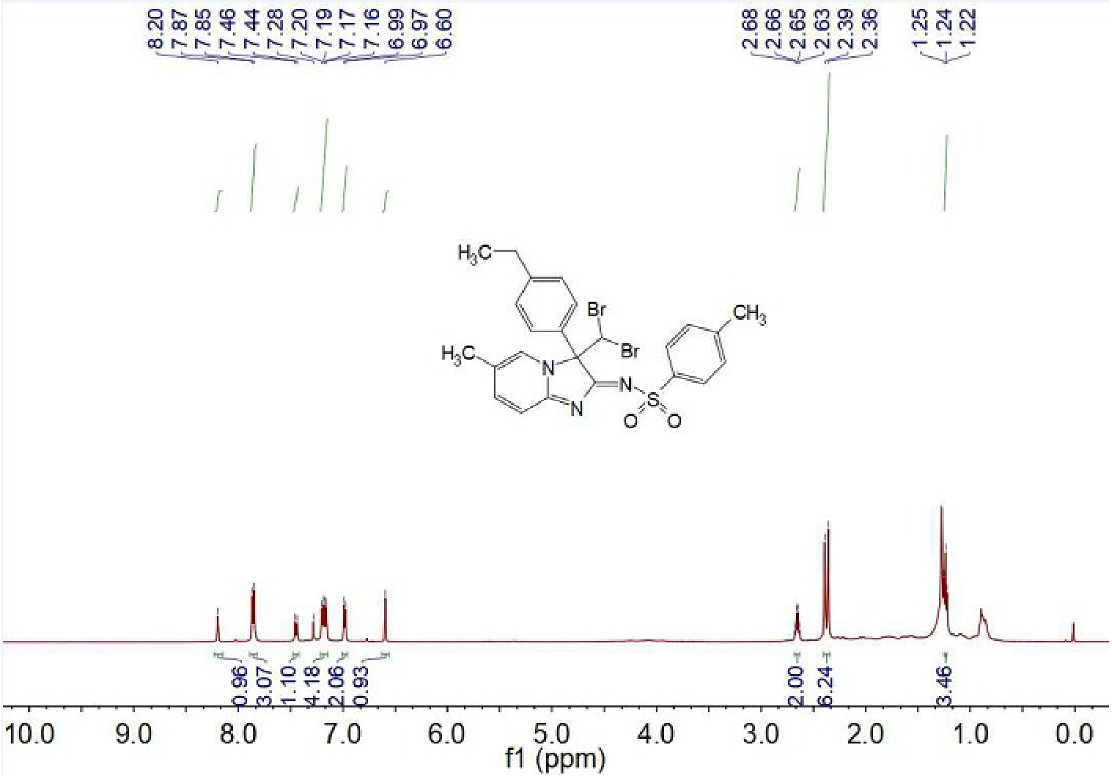

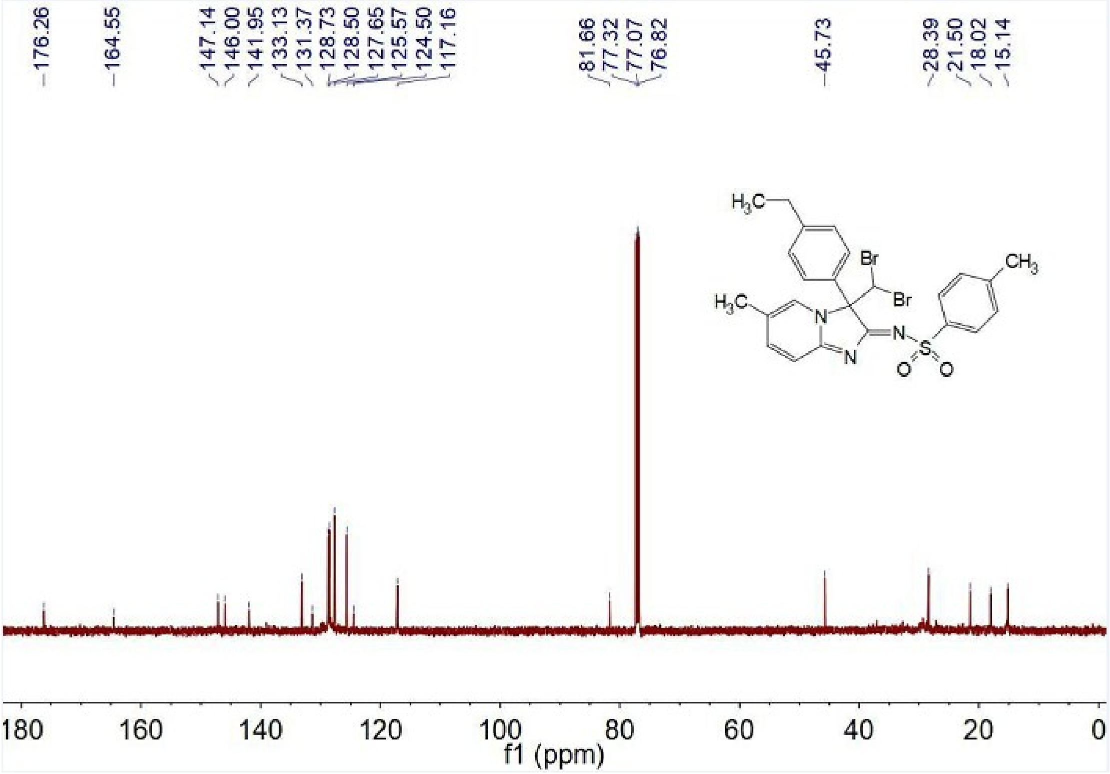

(Z)-N-(3-(dibromomethyl)-3-(4-methoxyphenyl)-6-methylimidazo[1,2-a]pyridin-2(3H)-ylidene)-4-methylbenzenesulfonamide(3m)

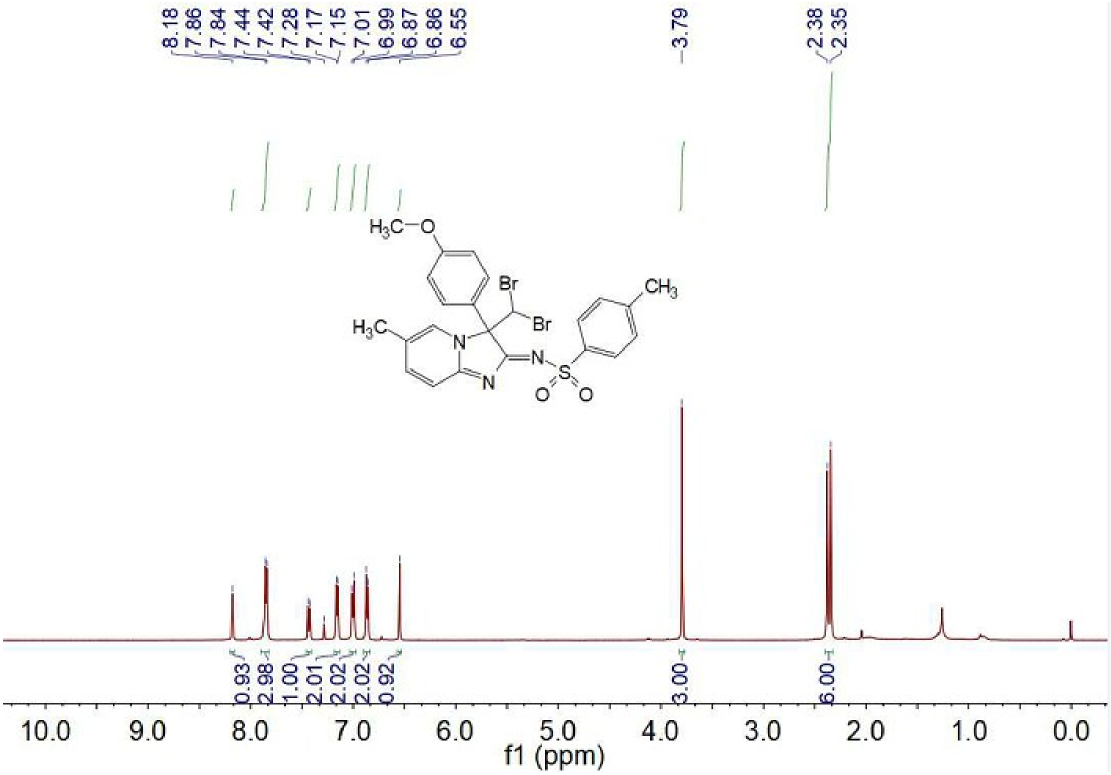

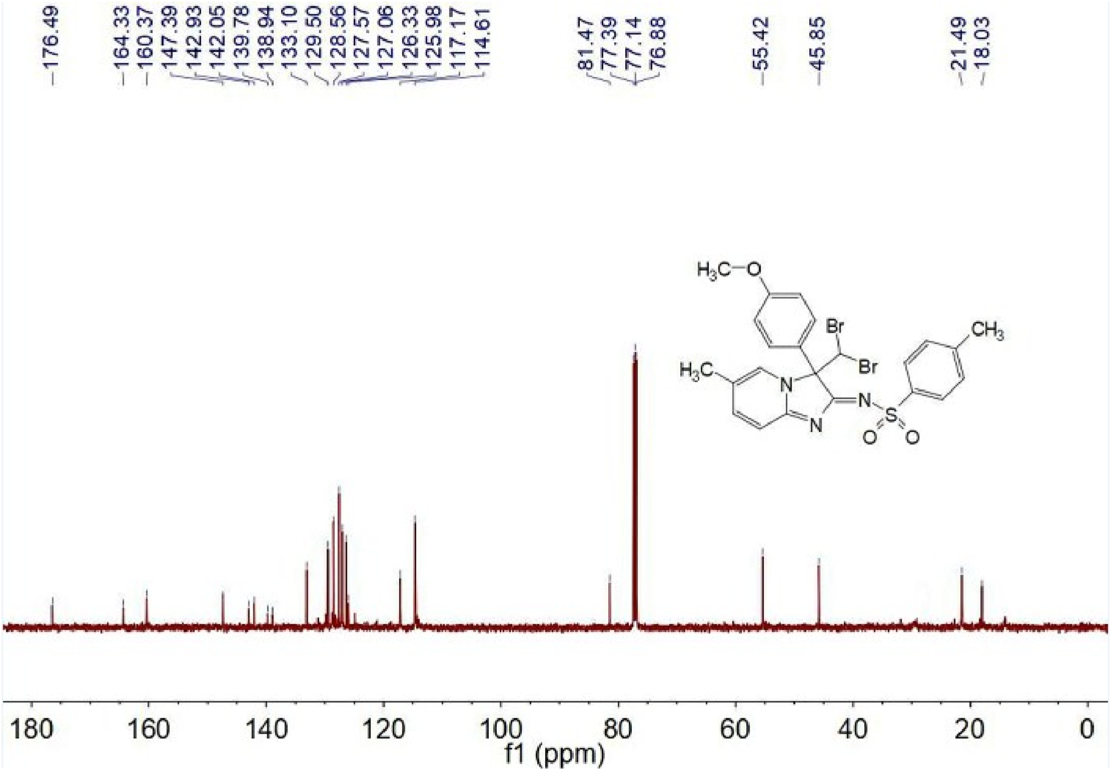

(Z)-N-(3-(4-chlorophenyl)-3-(dibromomethyl)-6-methylimidazo[1,2-a]pyridin-2(3H)-ylidene)-4-methylbenzenesulfonamide(3n)

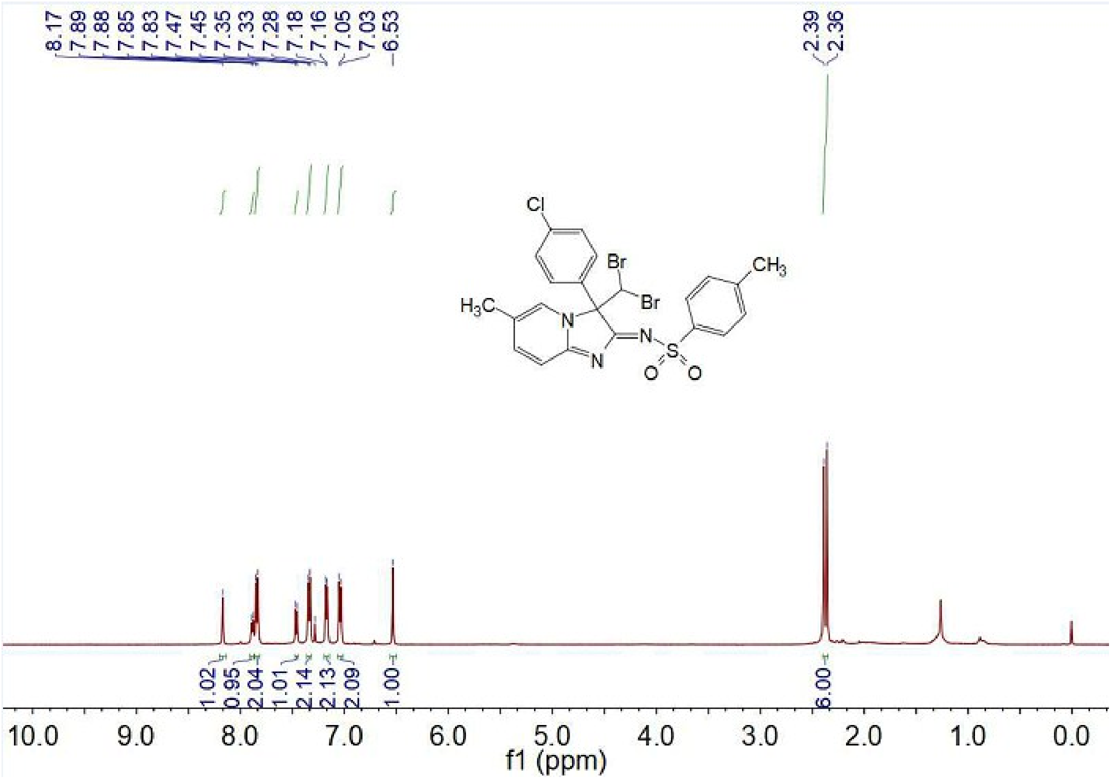

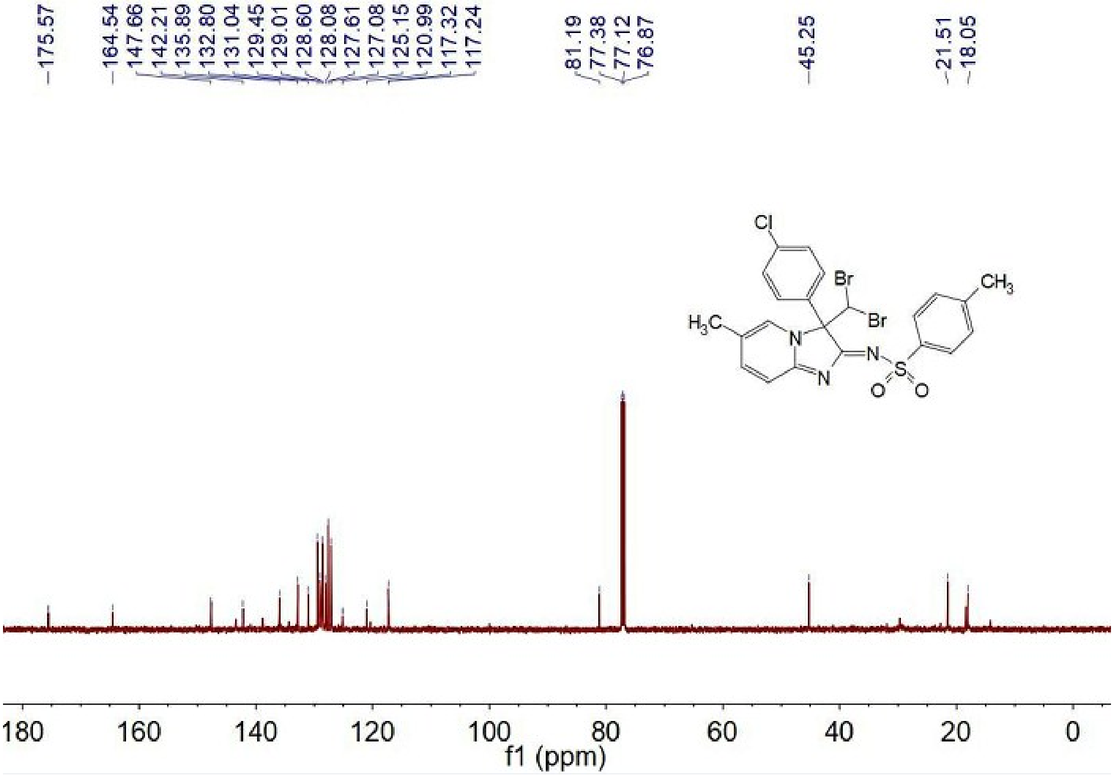

(Z)-N-(3-(dibromomethyl)-3-(3-fluorophenyl)-6-methylimidazo[1,2-a]pyridin-2(3H)-ylidene)-4-methylbenzenesulfonamide(3o)

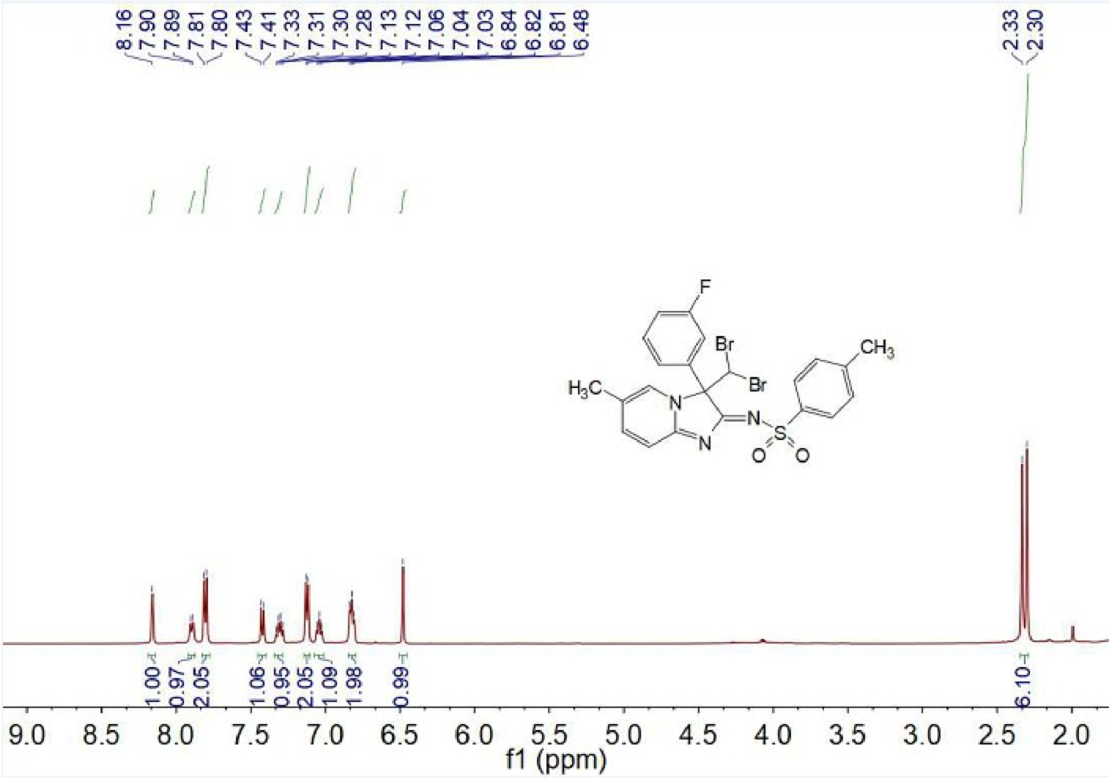

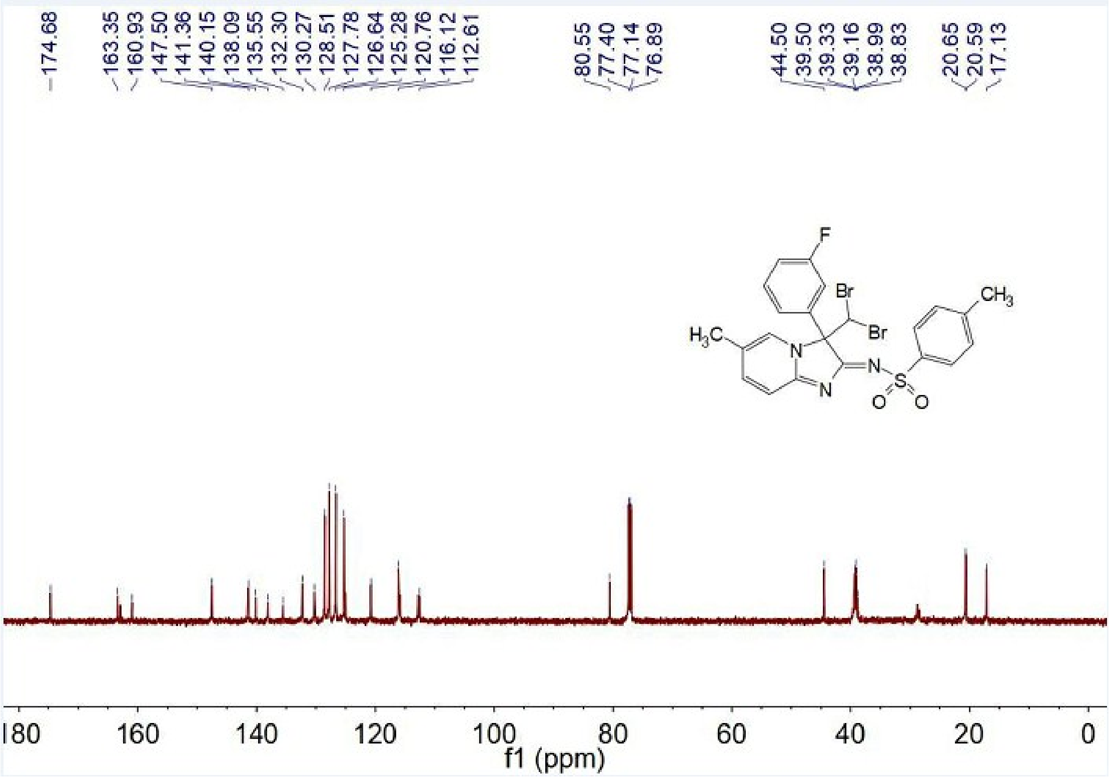

(Z)-N-(6-chloro-3-(dibromomethyl)-3-phenylimidazo[1,2-a]pyridin-2(3H)-ylidene)-4-methylbenzenesulfonamide(3p)

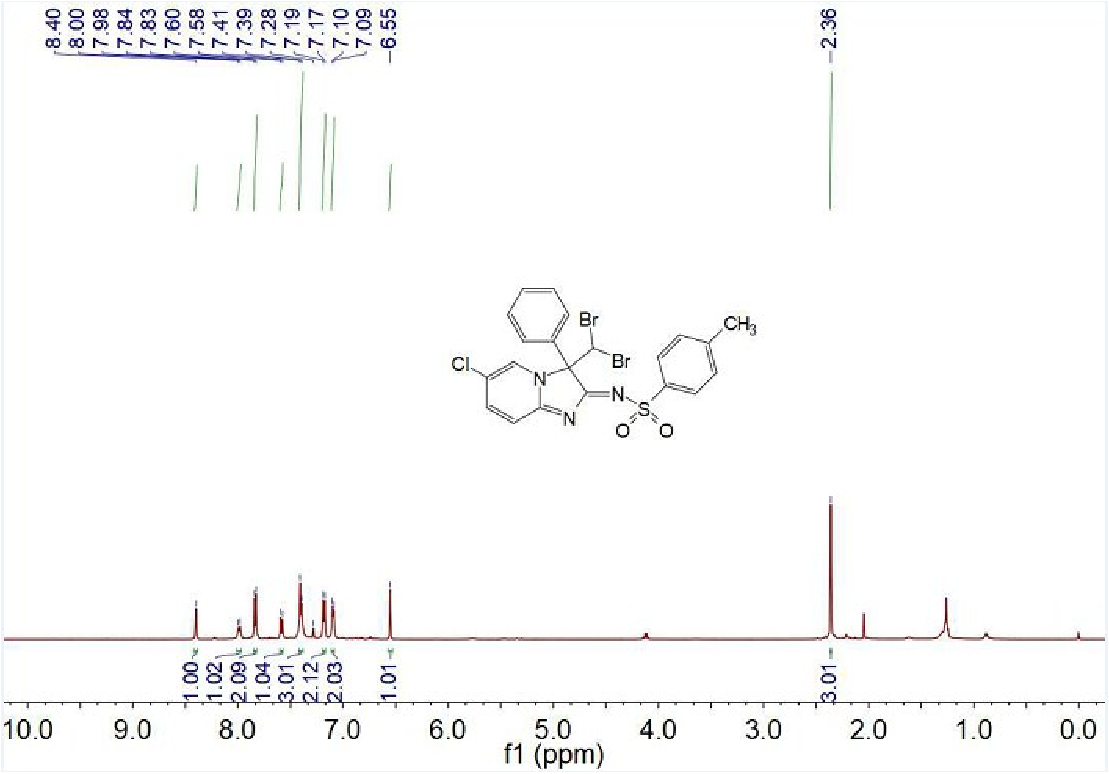

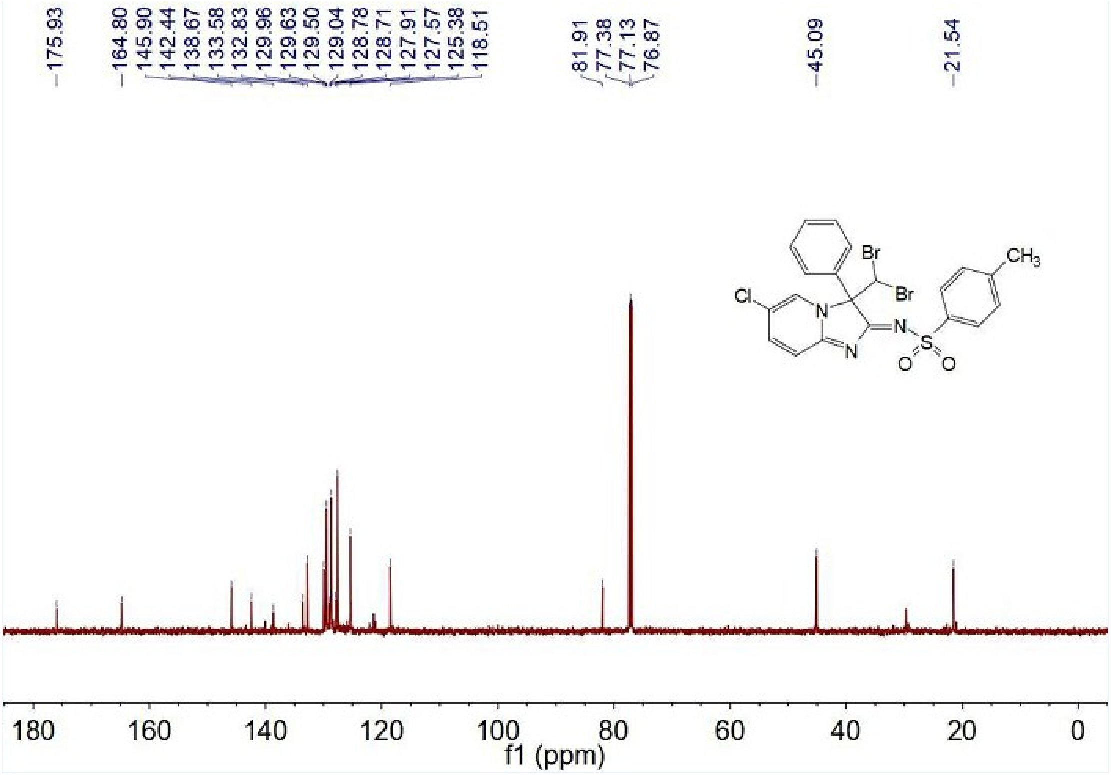

(Z)-N-(6-chloro-3-(dibromomethyl)-3-(4-ethylphenyl)imidazo[1,2-a]pyridin-2(3H)-ylidene)-4-methylbenzenesulfonamide(3q)

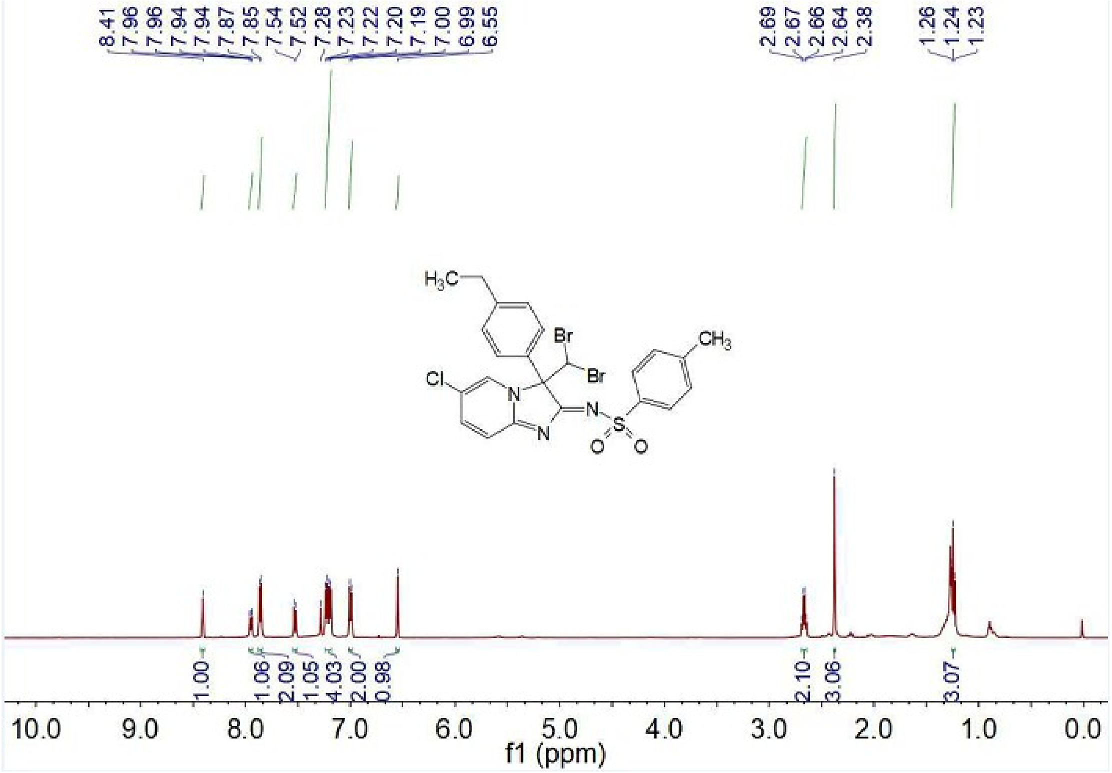

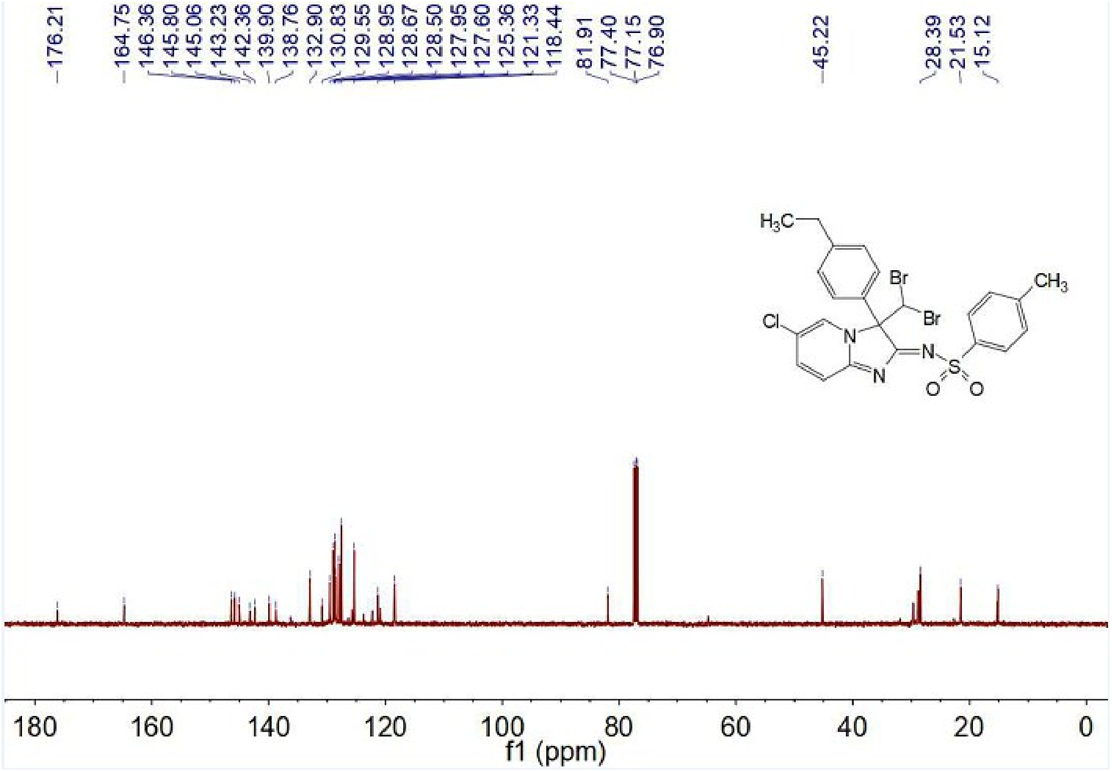

(Z)-N-(6-chloro-3-(dibromomethyl)-3-(m-tolyl)imidazo[1,2-a]pyridin-2(3H)-ylidene)-4-methylbenzenesulfonamide(3r)

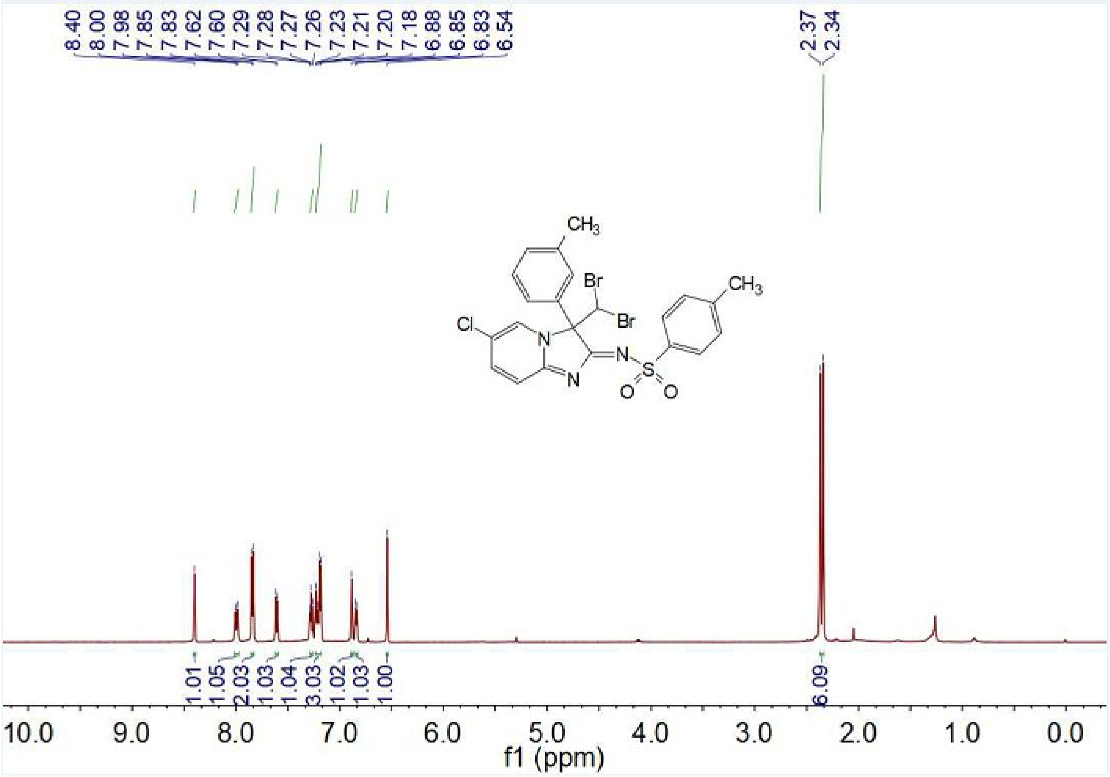

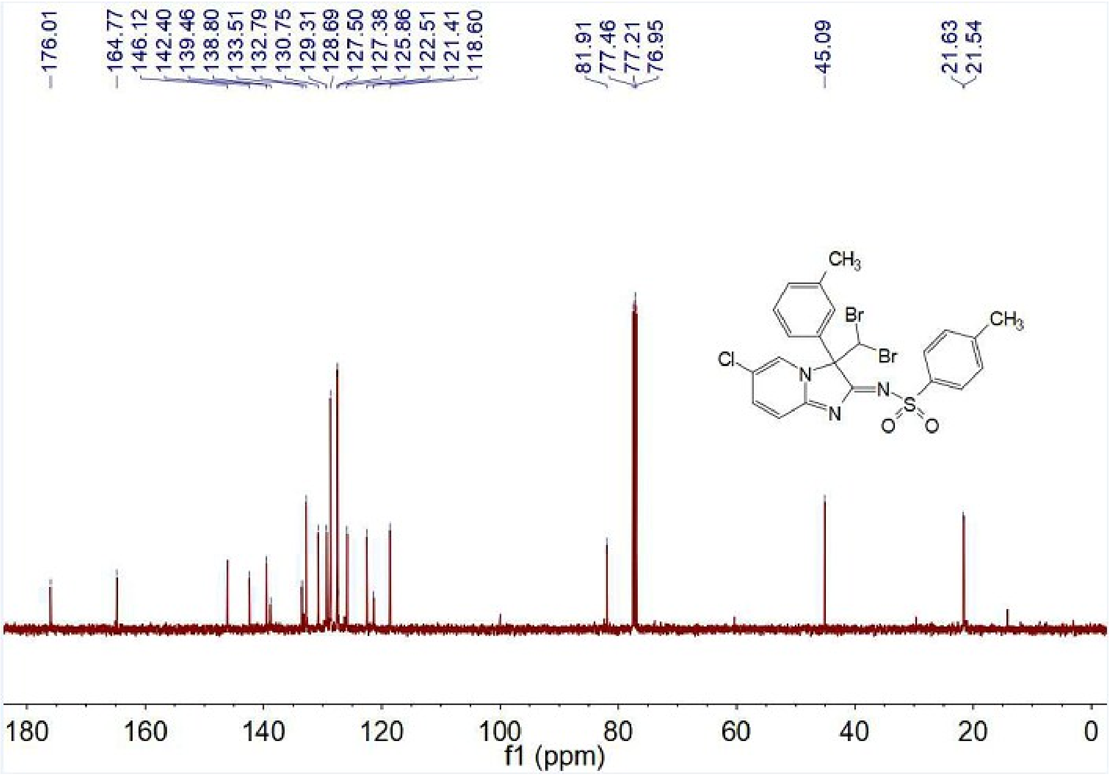

(Z)-N-(6-chloro-3-(dibromomethyl)-3-(3-methoxyphenyl)imidazo[1,2-a]pyridin-2(3H)-ylidene)-4-methylbenzenesulfonamide(3s)

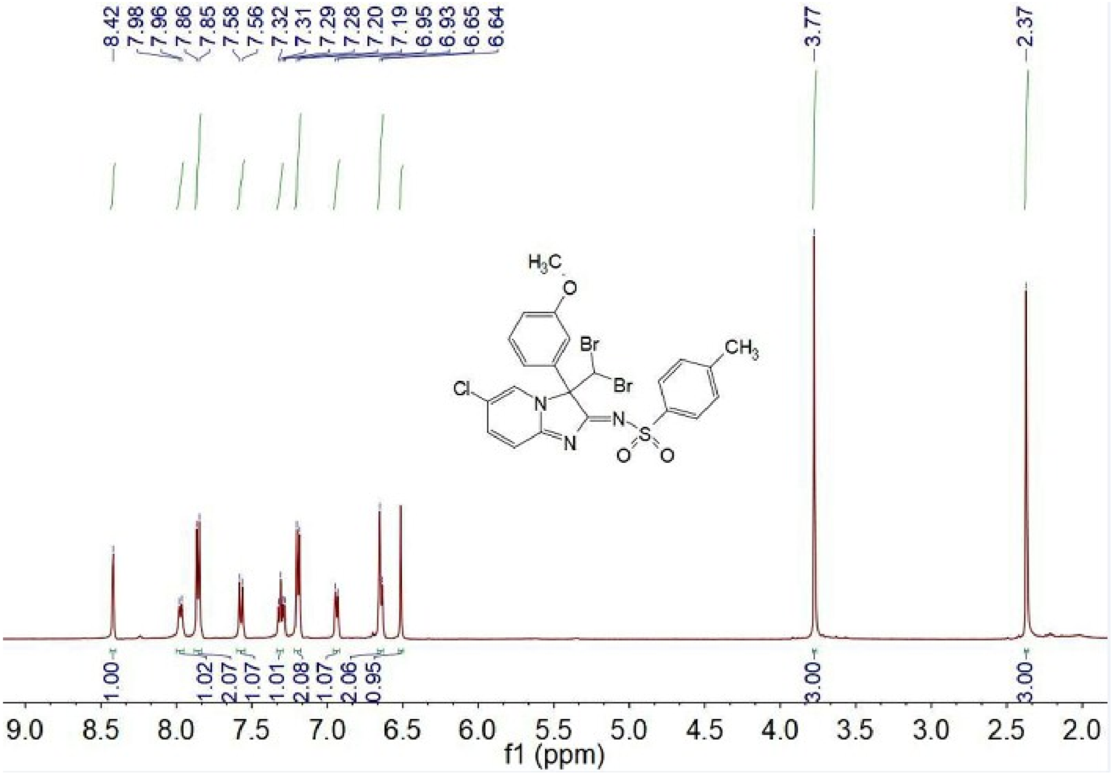

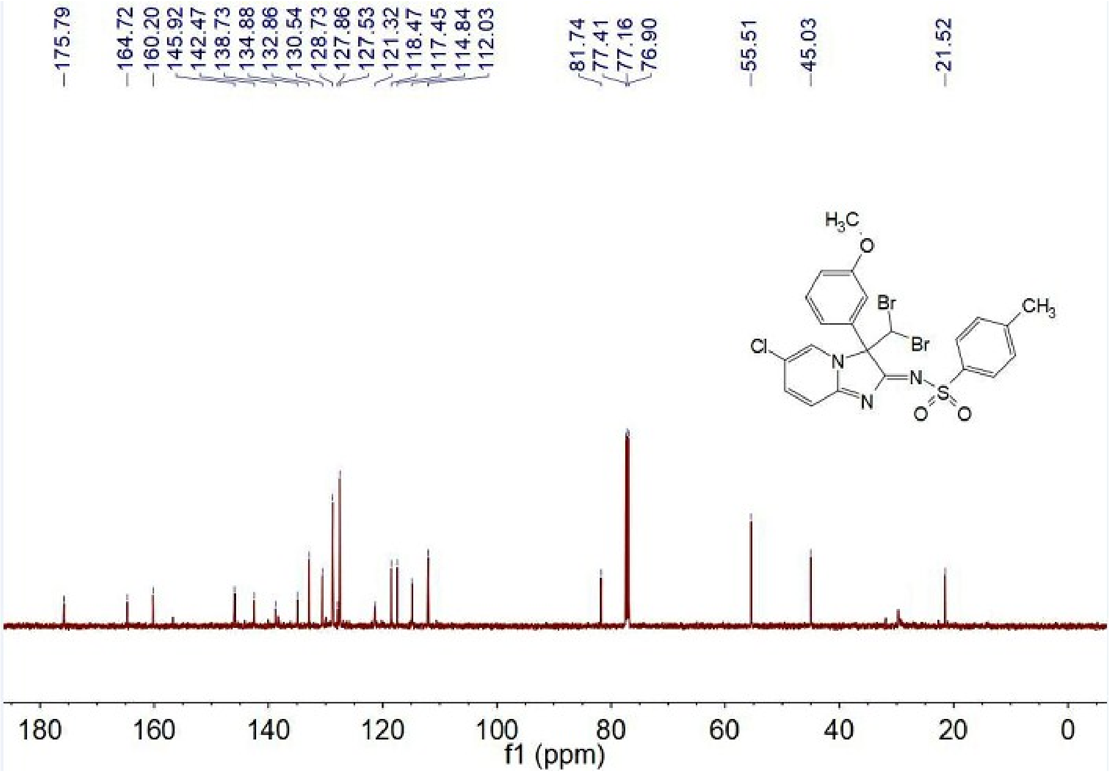

(Z)-N-(6-chloro-3-(dibromomethyl)-3-(4-methoxyphenyl)imidazo[1,2-a]pyridin-2(3H)-ylidene)-4-methylbenzenesulfonamide(3t)

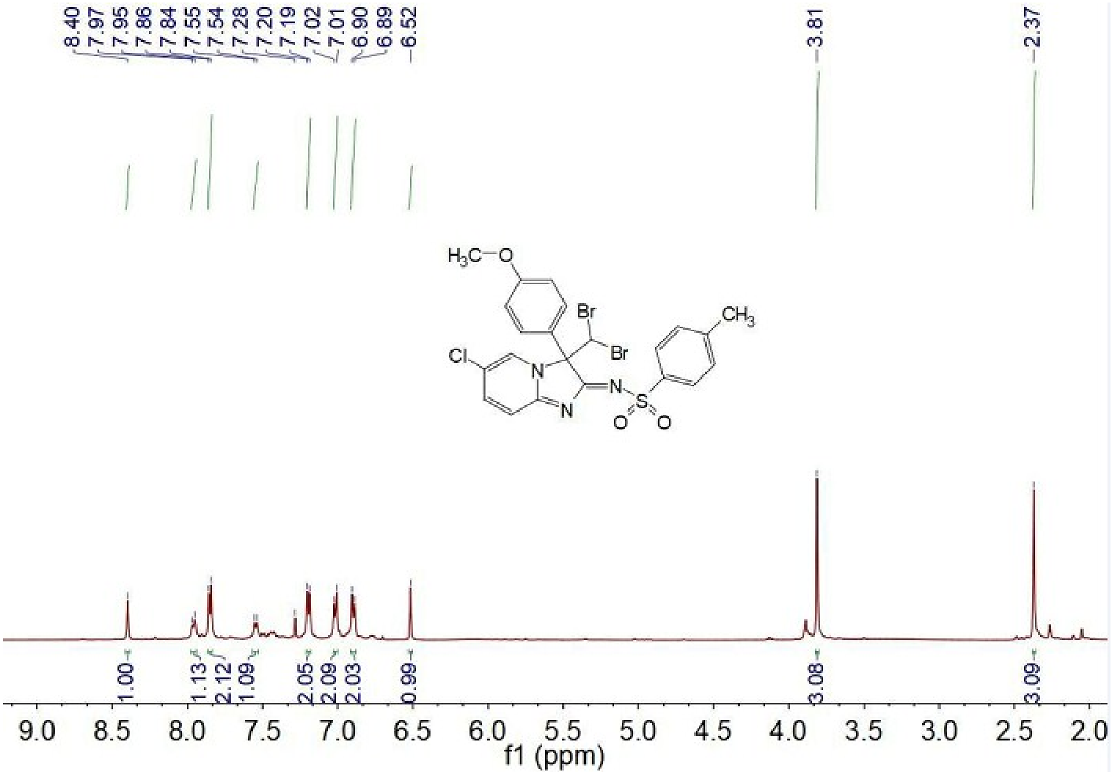

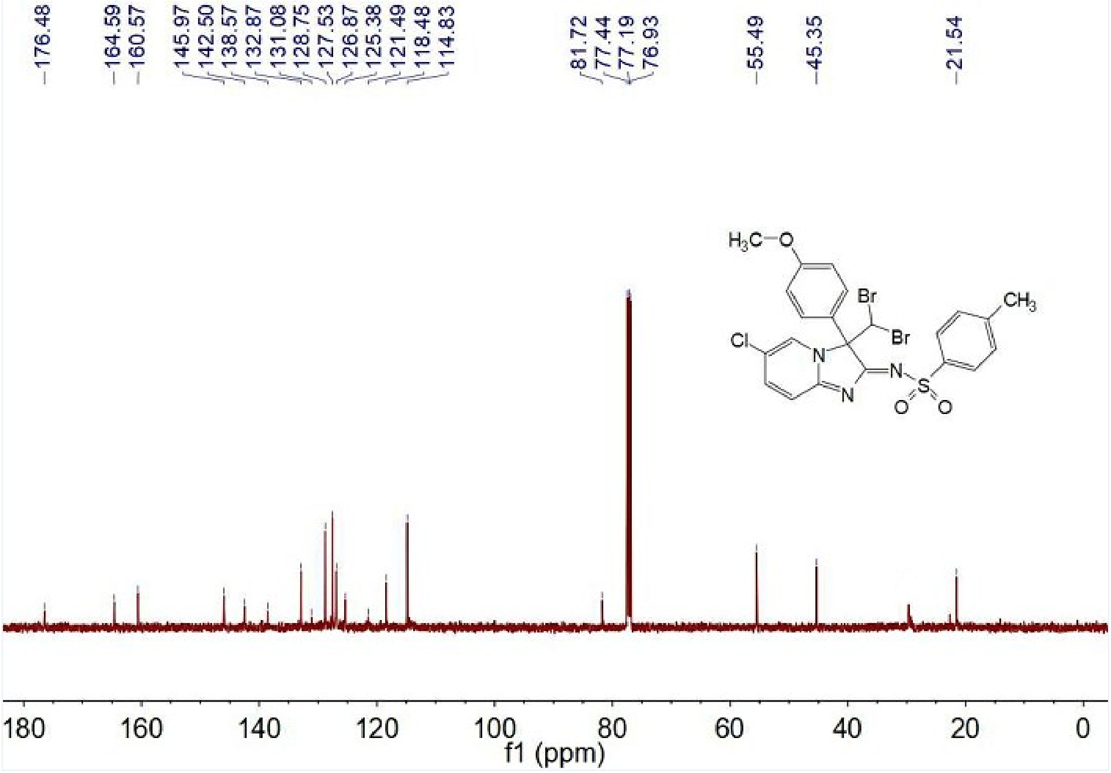

(Z)-N-(6-chloro-3-(3-chlorophenyl)-3-(dibromomethyl)imidazo[1,2-a]pyridin-2(3H)-ylidene)-4-methylbenzenesulfonamide(3u)

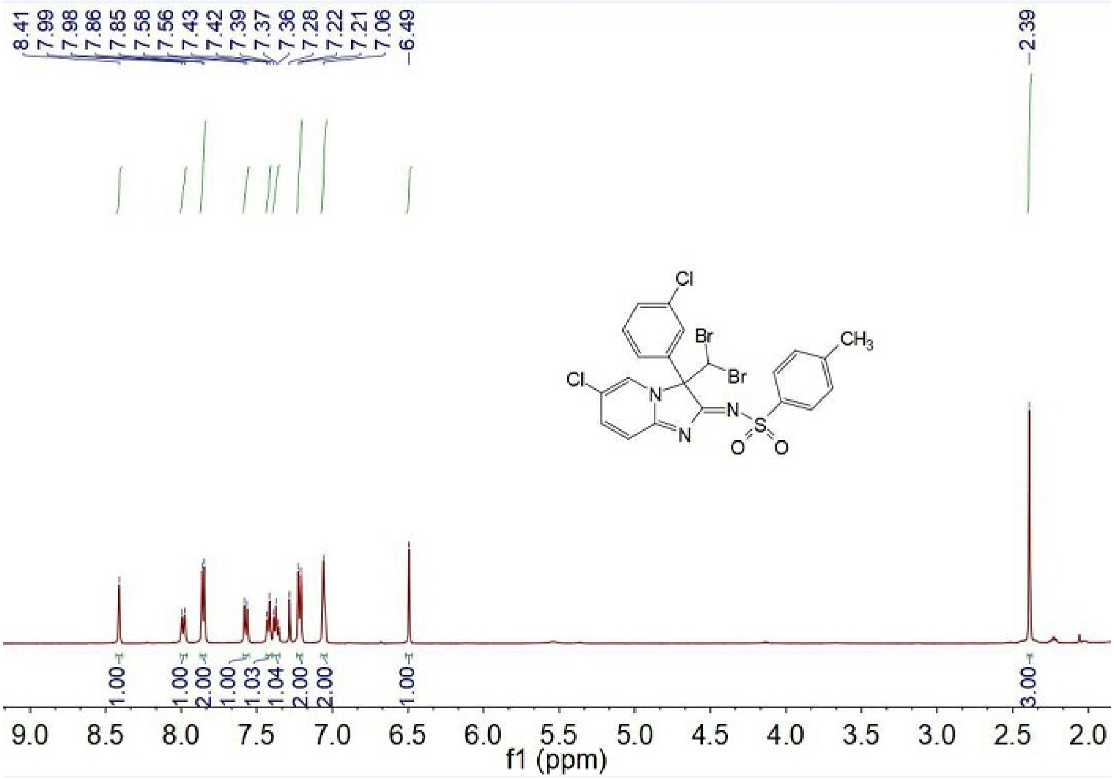

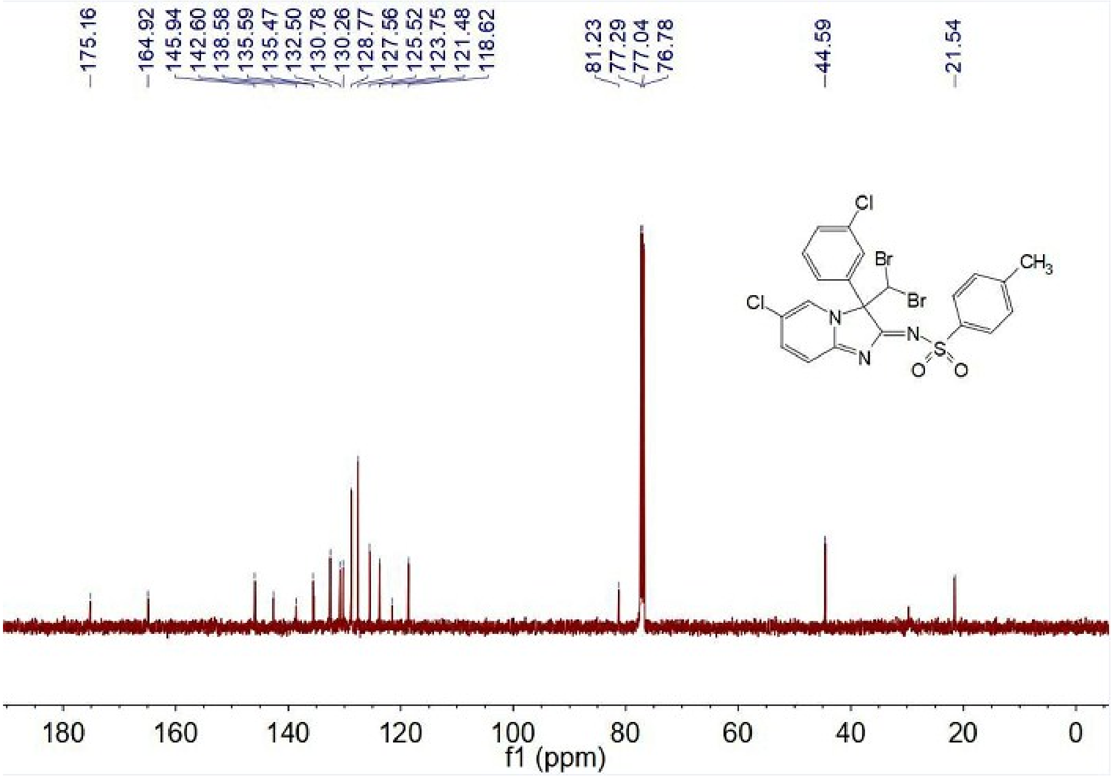

(Z)-N-(6-chloro-3-(dibromomethyl)-3-(3-fluorophenyl)imidazo[1,2-a]pyridin-2(3H)-ylidene)-4-methylbenzenesulfonamide(3v)

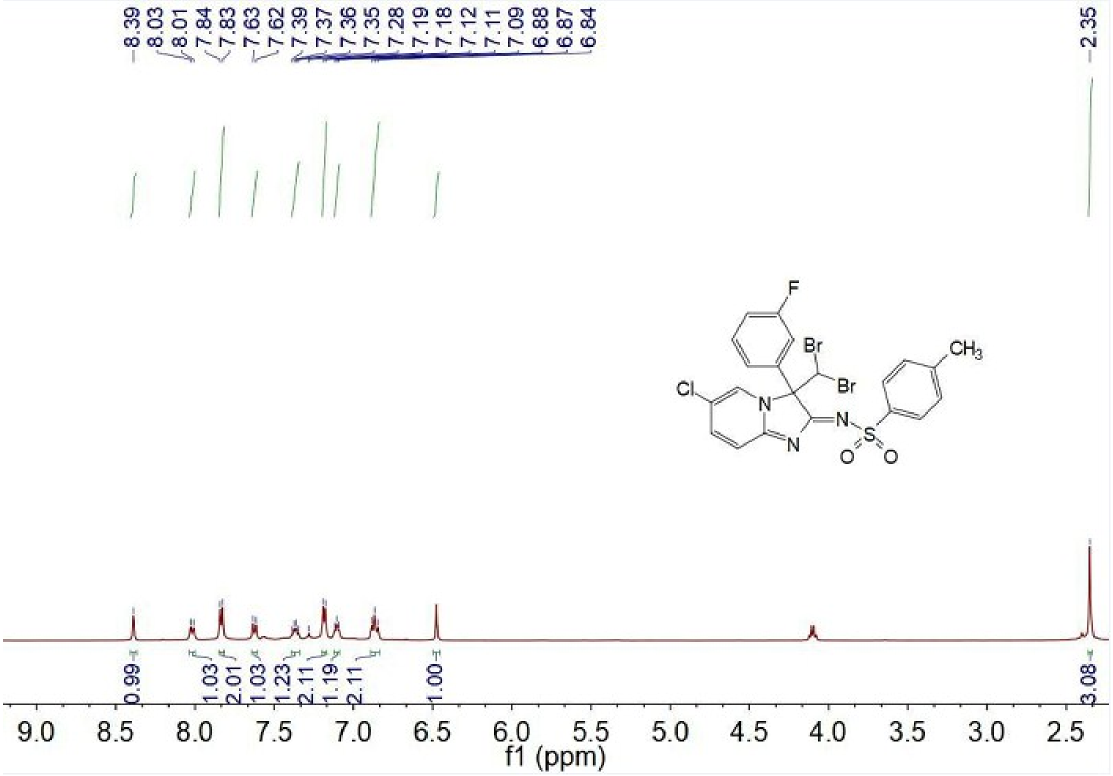

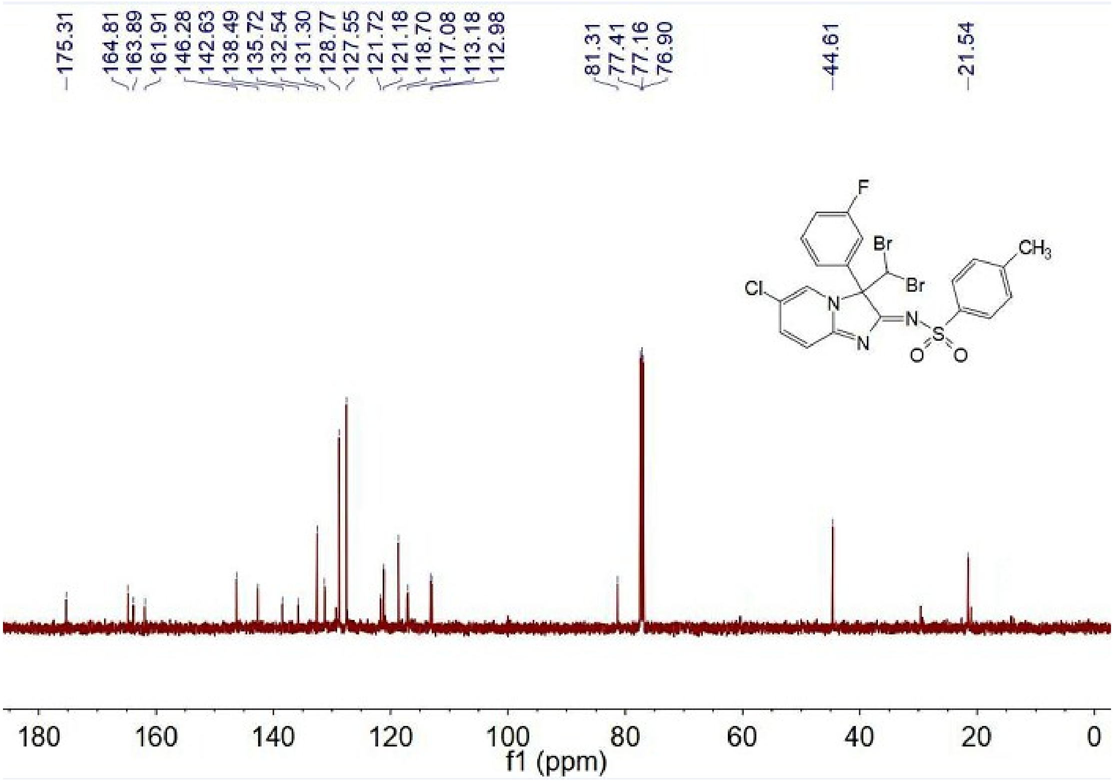

(Z)-N-(3-(dibromomethyl)-3-phenylimidazo[1,2-a]pyridin-2(3H)-ylidene)methanesulfonamide(3w)

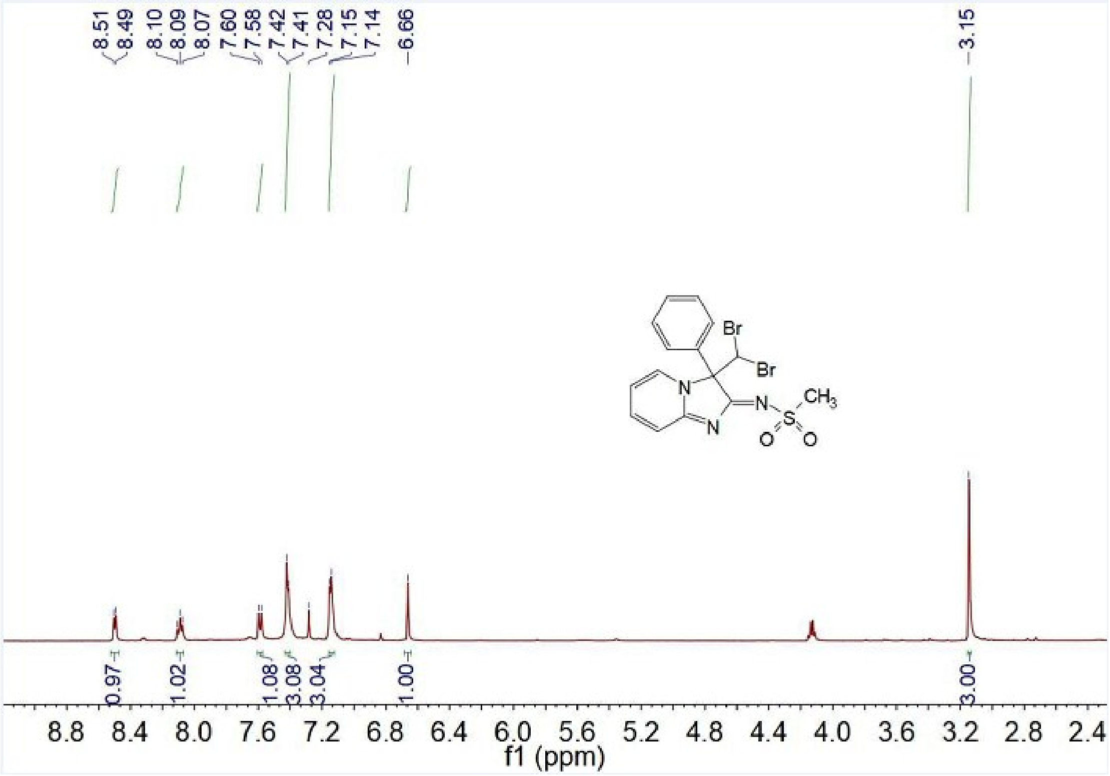

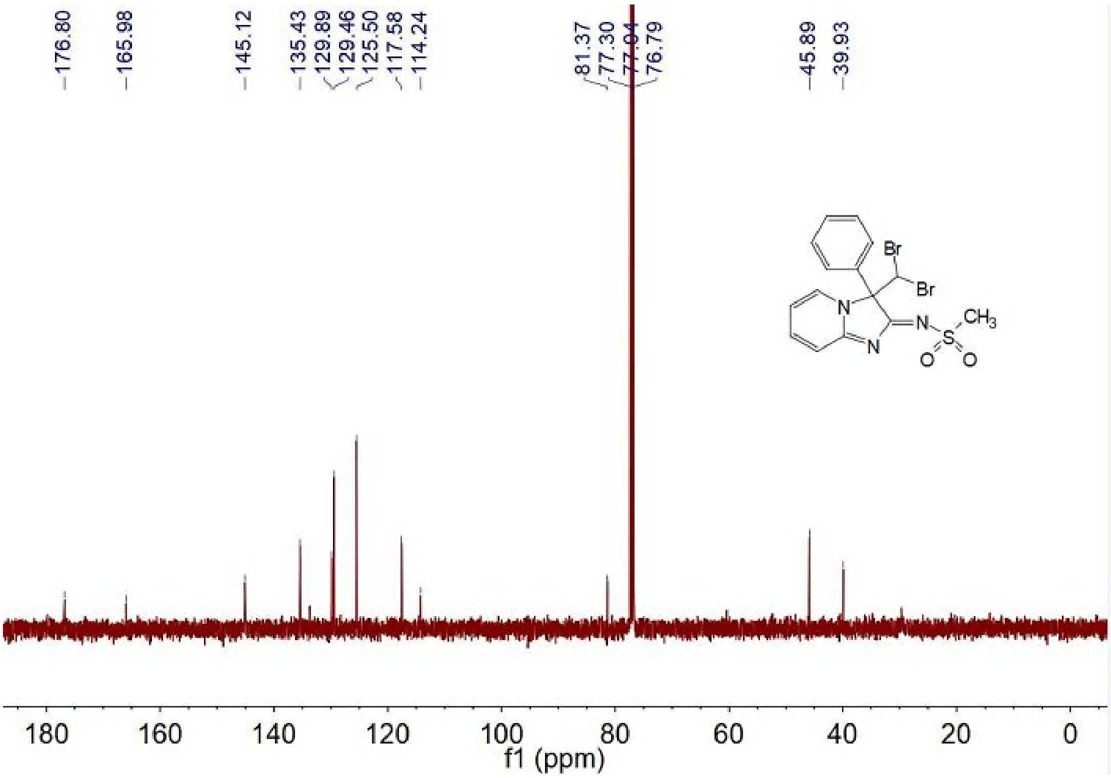

(Z)-N-(3-(dibromomethyl)-3-phenylimidazo[1,2-a]pyridin-2(3H)-ylidene)benzenesulfonamide(3x)

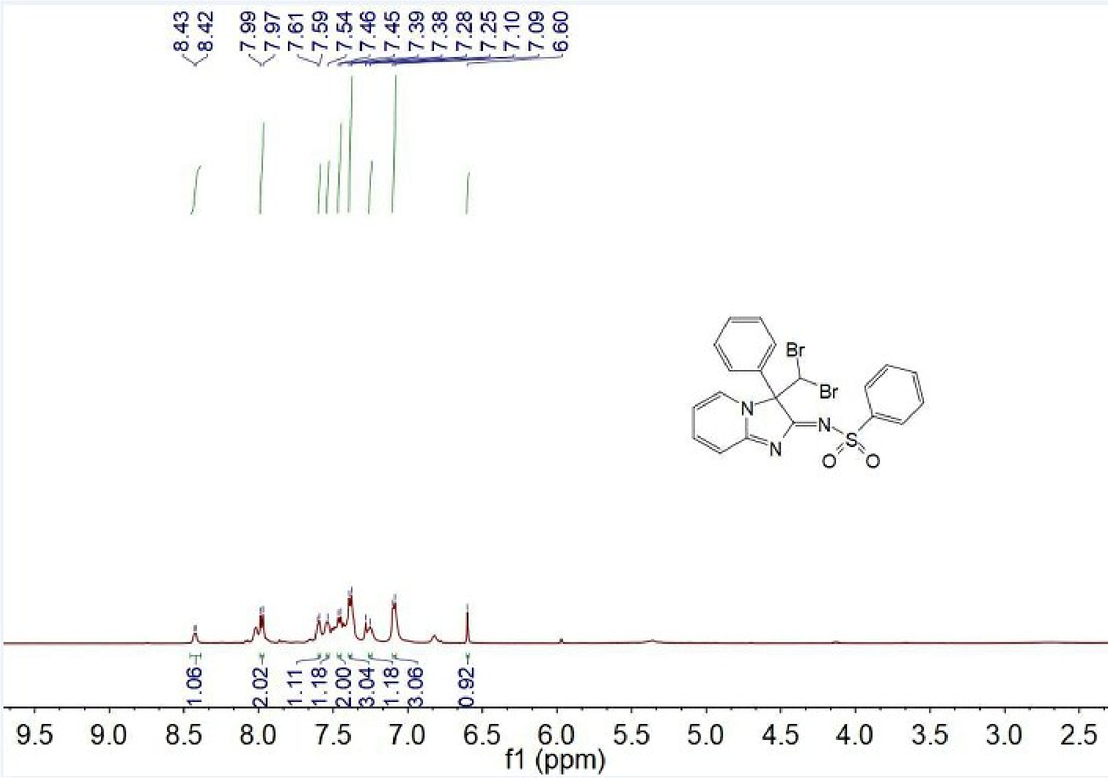

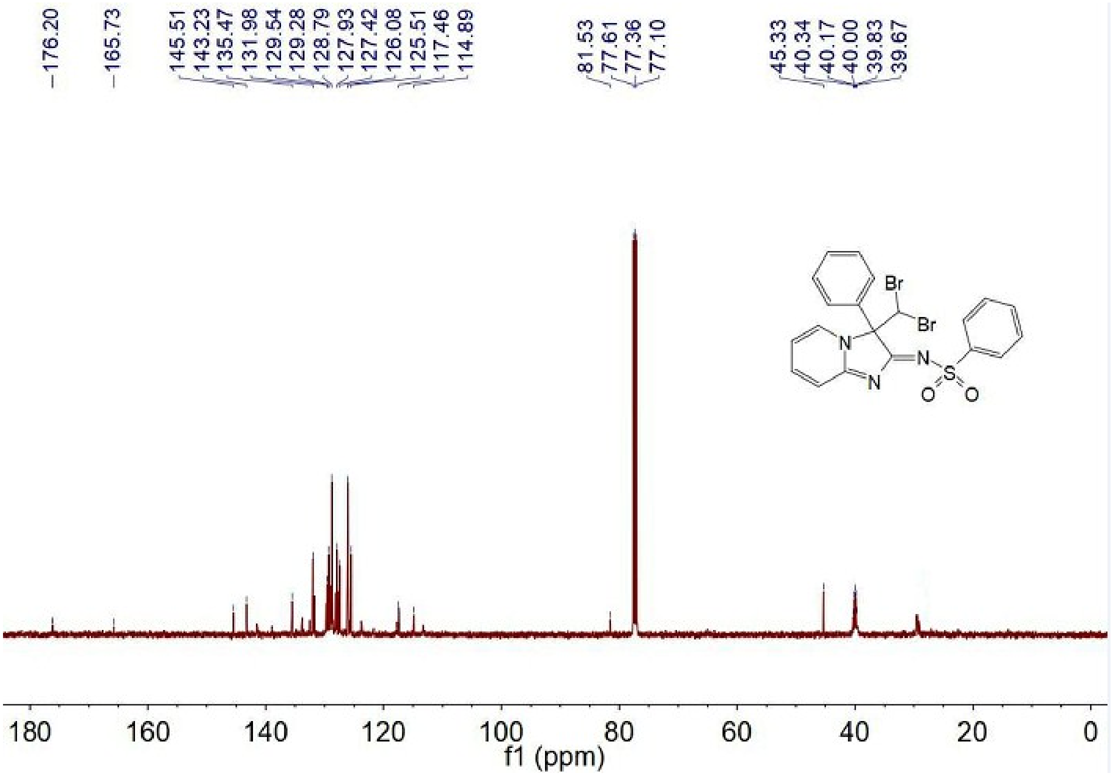

(Z)-N-(3-(dibromomethyl)-3-(4-ethylphenyl)imidazo[1,2-a]pyridin-2(3H)-ylidene)benzenesulfonamide(3y)

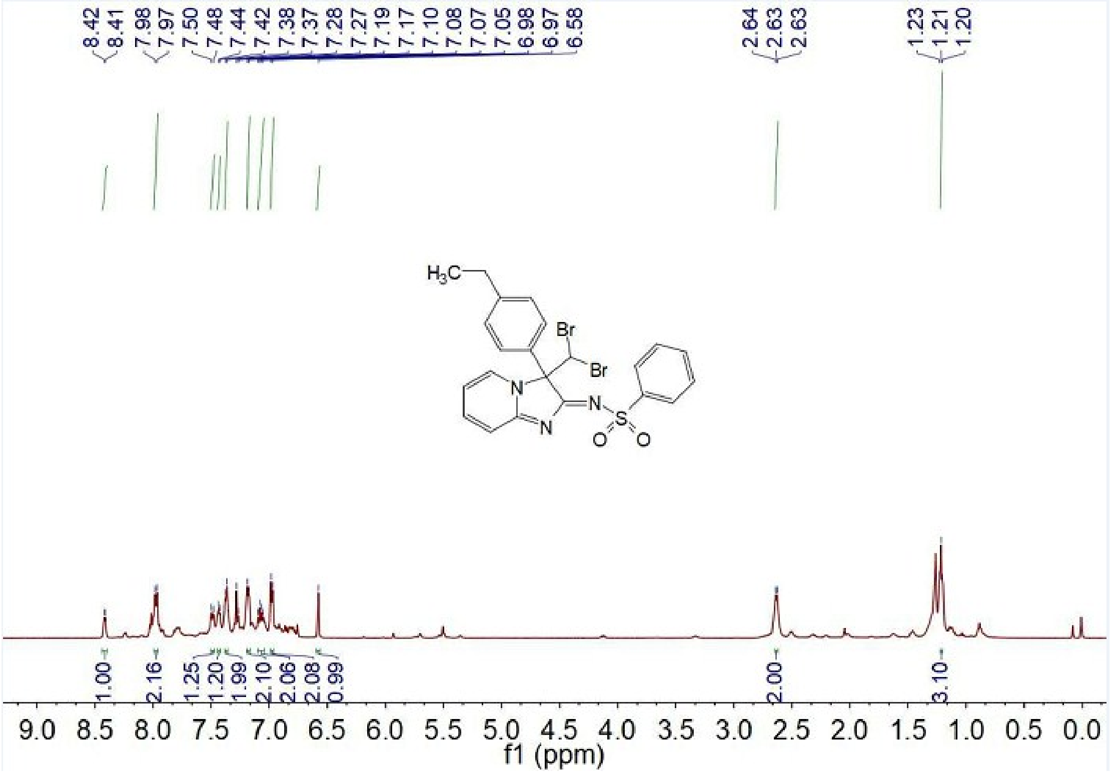

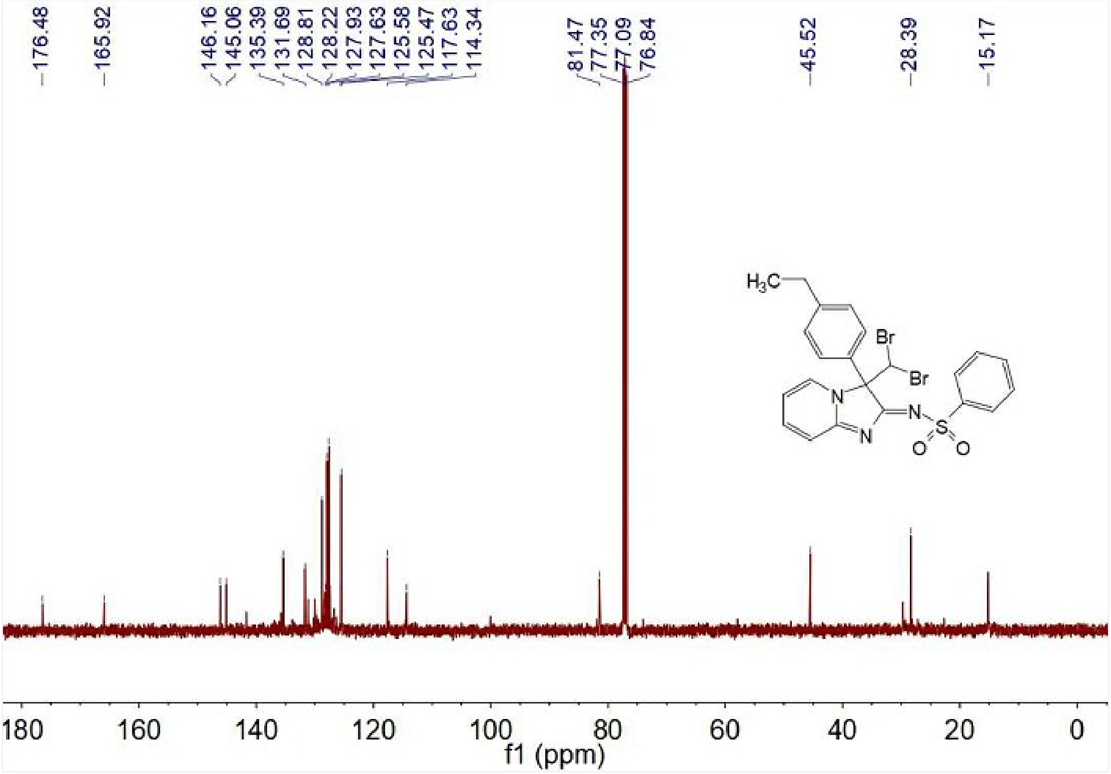

(Z)-N-(3-(dibromomethyl)-6-methyl-3-phenylimidazo[1,2-a]pyridin-2(3H)-ylidene)benzenesulfonamide(3z)

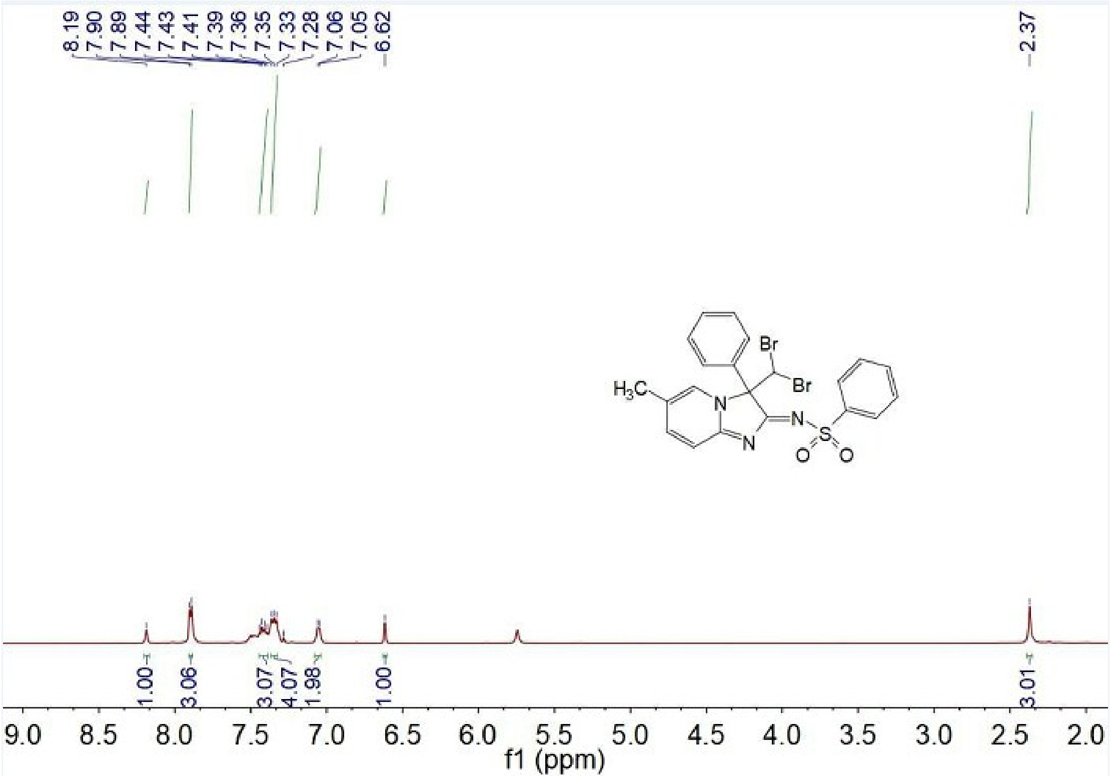

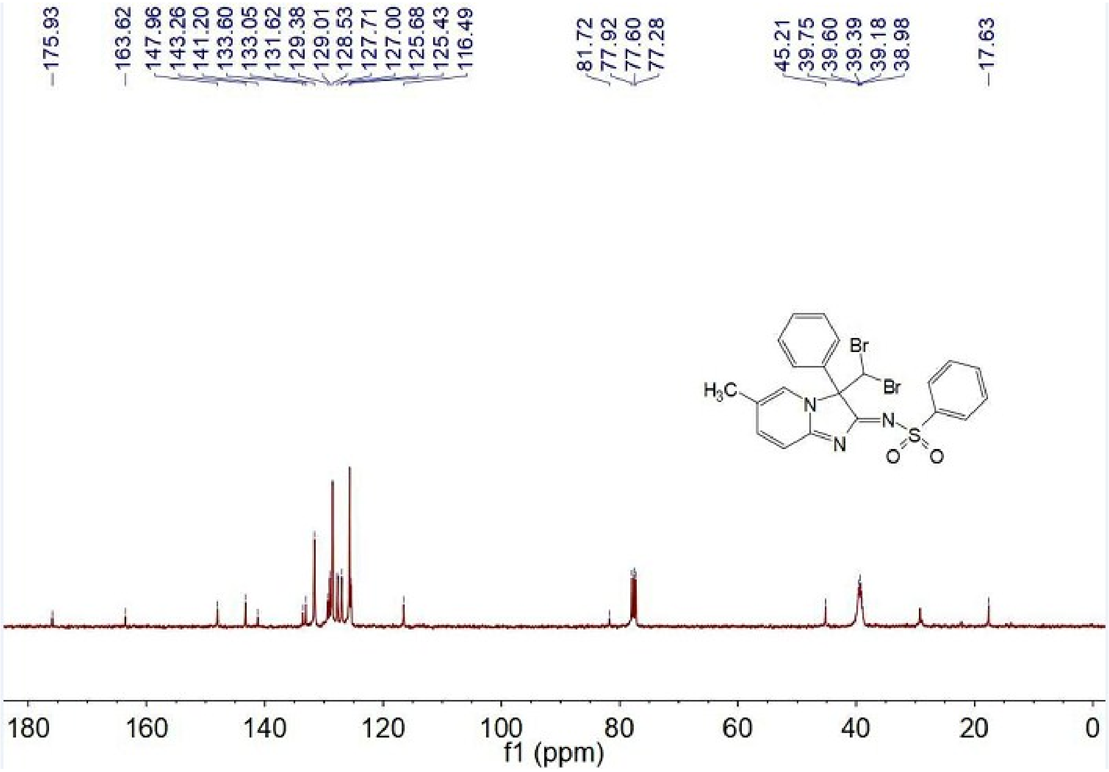

(Z)-N-(6-chloro-3-(dibromomethyl)-3-phenylimidazo[1,2-a]pyridin-2(3H)-ylidene)benzenesulfonamide(3ab)

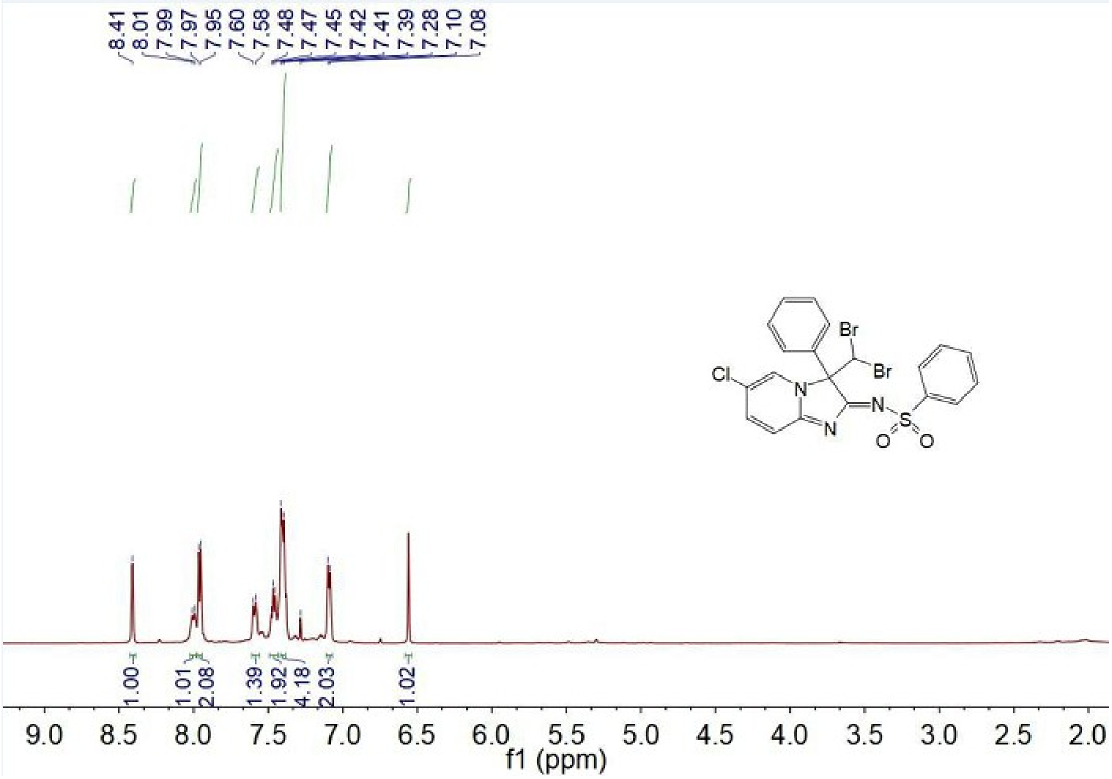

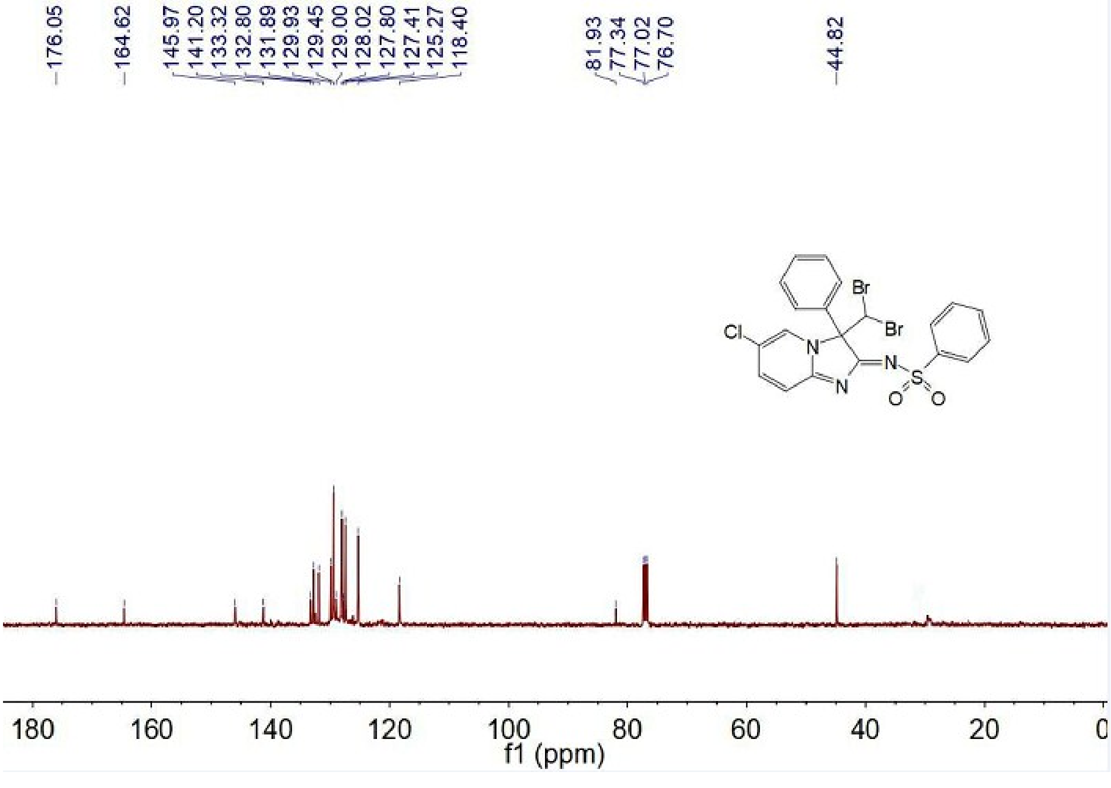

(Z)-N-(6-chloro-3-(dibromomethyl)-3-(4-ethylphenyl)imidazo[1,2-a]pyridin-2(3H)-ylidene)benzenesulfonamide(3ac)

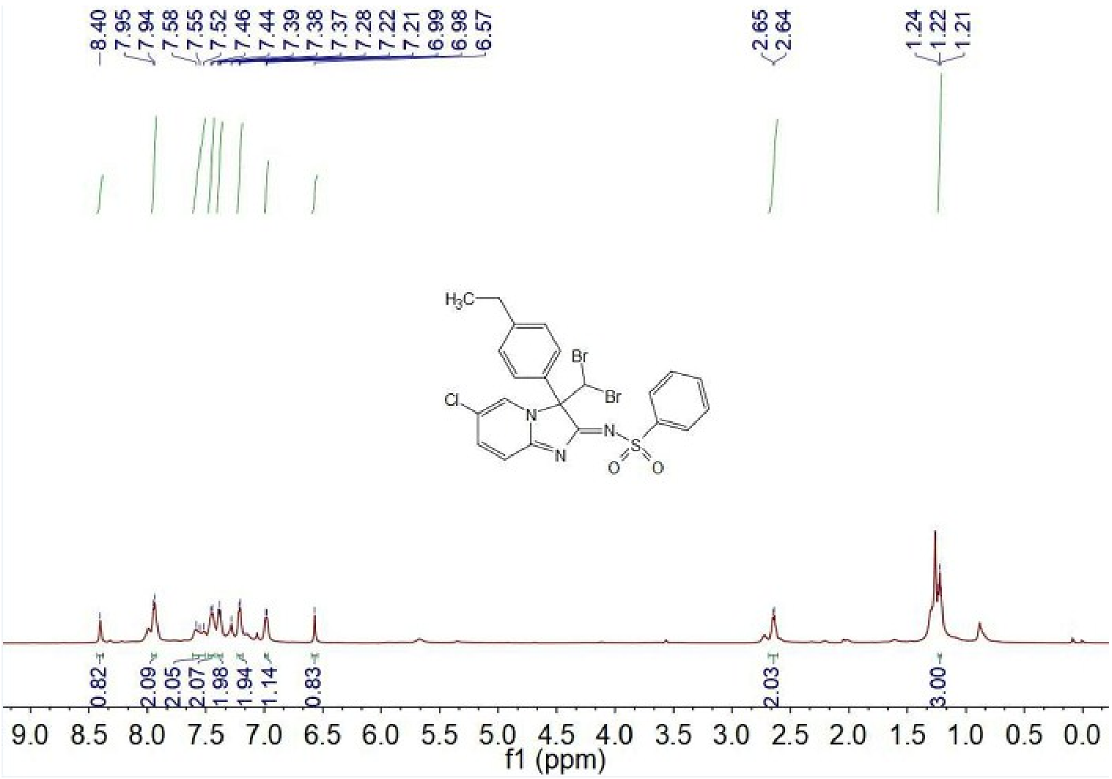

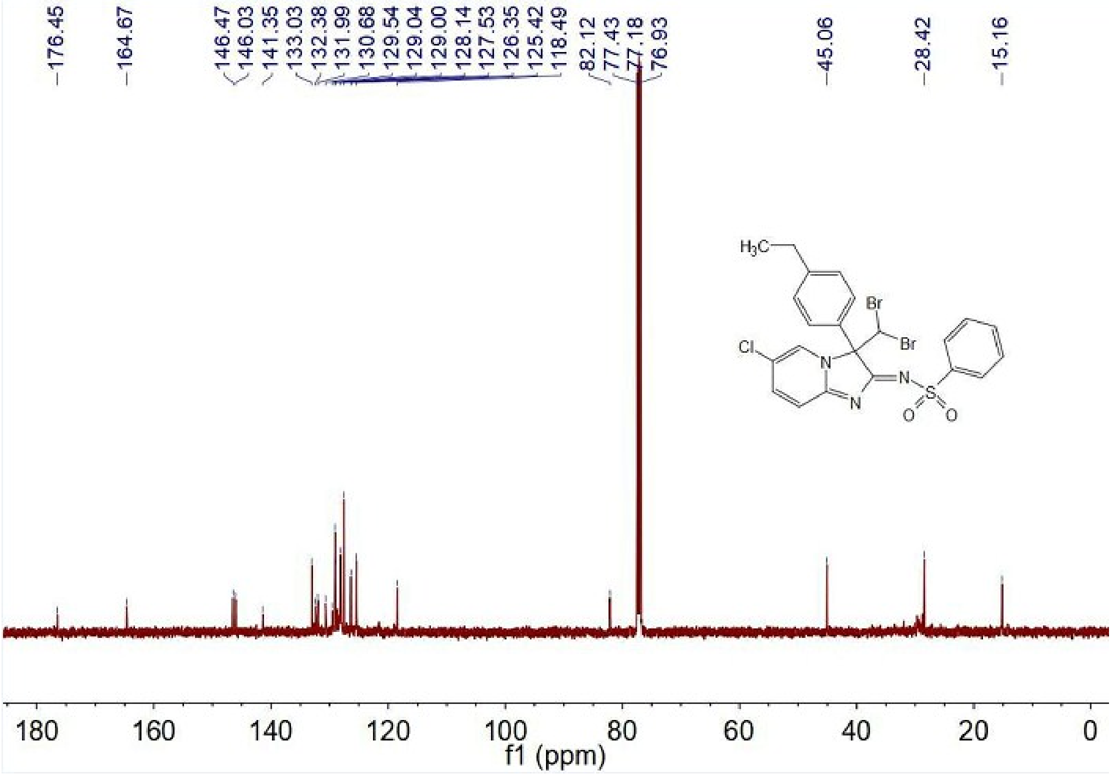

(Z)-N-(6-chloro-3-(4-chlorophenyl)-3-(dibromomethyl)imidazo[1,2-a]pyridin-2(3H)-ylidene)benzenesulfonamide(3ad)

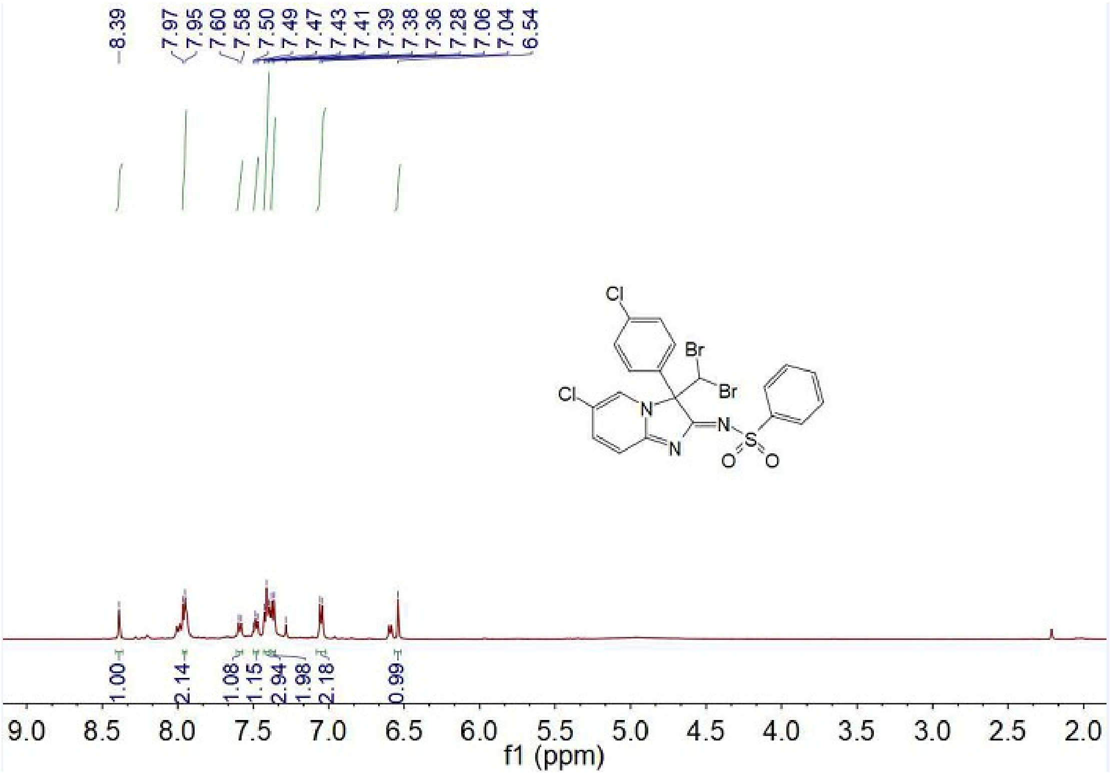

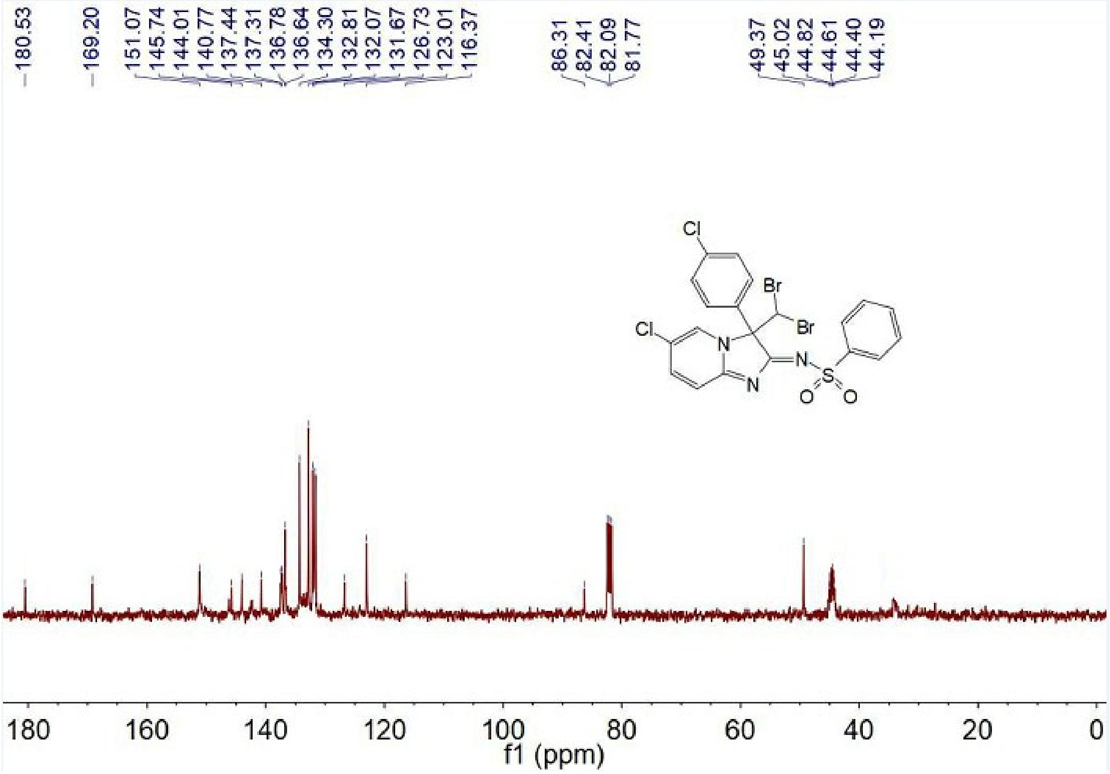

Impurity in eluent or silica gel

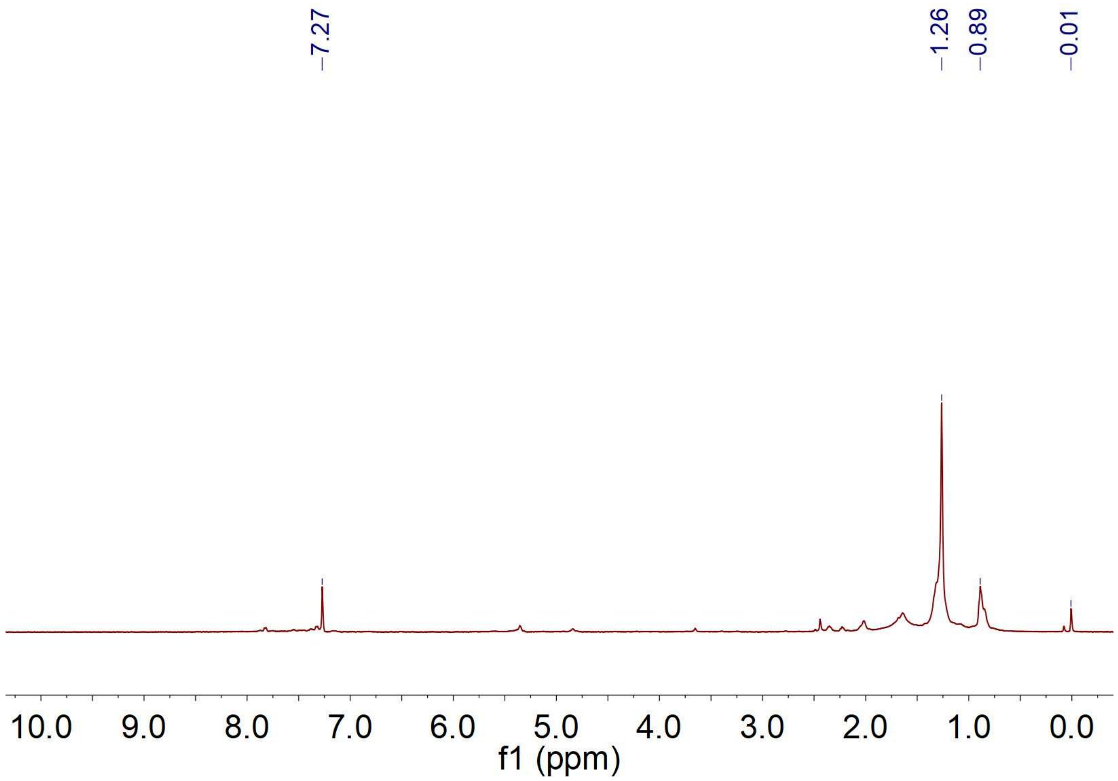

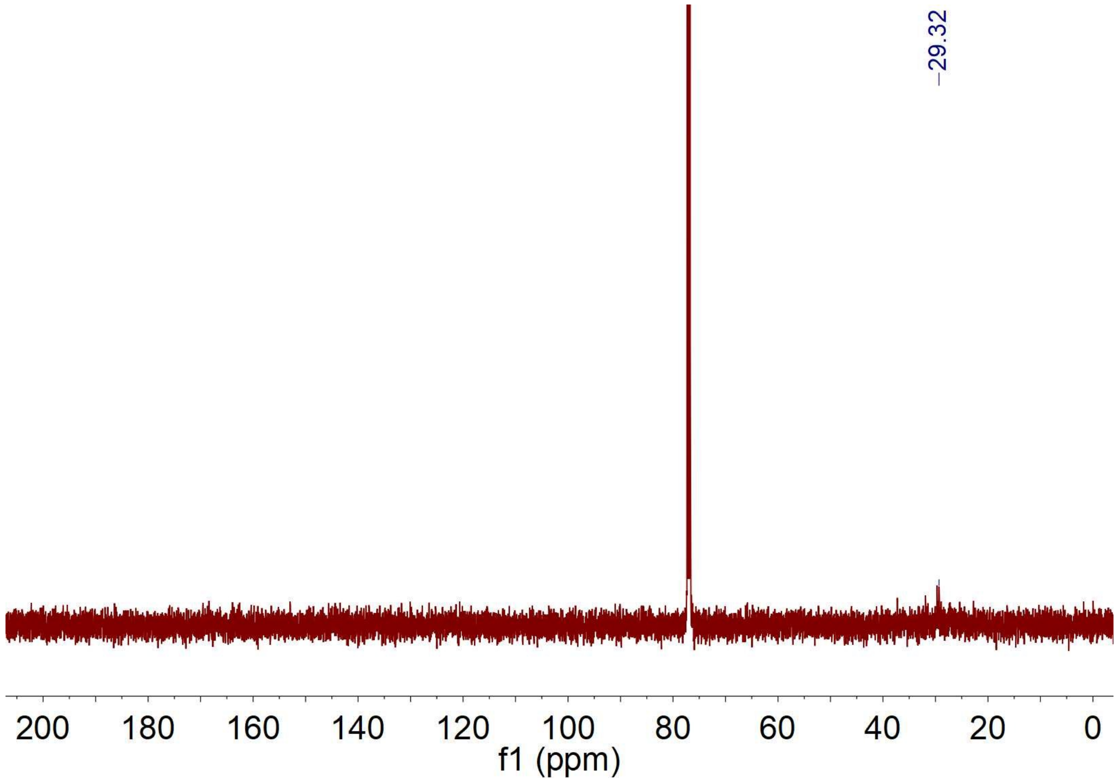

Supplement: RA-009-C9RA09265J-s001 [file RA-009-C9RA09265J-s001.pdf]
